# Preclinical Characterization of GLS-010 (Zimberelimab), a Novel Fully Human Anti-PD-1 Therapeutic Monoclonal Antibody for Cancer

**DOI:** 10.3389/fonc.2021.736955

**Published:** 2021-09-15

**Authors:** Beilei Lou, Hua Wei, Fang Yang, Shicong Wang, Baotian Yang, Yong Zheng, Jiman Zhu, Shaoyu Yan

**Affiliations:** ^1^R&D Department, Guangzhou Gloria Biosciences Co. Ltd., Beijing, China; ^2^Medical Affairs Department, Guangzhou Gloria Biosciences Co. Ltd., Beijing, China; ^3^Biologics Innovation & Discovery Department, WuXi Biologics, Wuxi, China; ^4^Board of Directors, Guangzhou Gloria Biosciences Co. Ltd., Beijing, China

**Keywords:** GLS-010, zimberelimab, immunotherapy, preclinical study, immune checkpoint inhibitor

## Abstract

**Background:**

Zimberelimab (GLS-010) is a novel fully human monoclonal immunoglobulin G4 (IgG4) against the programmed cell death-1 (PD-1) receptor.

**Aim:**

To evaluate the affinity, competitive blocking capability, T cell activation effect, cytotoxic effector functions by Fc, preliminary anti-tumor activity, and pharmacokinetics of GLS-010.

**Methods:**

The affinity of GLS-010 to PD-1 and the ability of GLS-010 to block the PD-L1/2 to PD-1 interaction on the cell surface were measured. An allogeneic mixed lymphocyte reaction was conducted to evaluate the inhibitory effect of GLS-010 on Tregs and stimulatory effect on T cell proliferation and activation. Pharmacodynamics and pharmacokinetics were evaluated in tumor-bearing mice and cynomolgus monkeys, respectively.

**Results:**

The equilibrium dissociation constant (KD) for the association between GLS-010 and PD-1 was 1.75×10^-10^ M. GLS-010 could effectively block the binding of PD-L1/2 to PD-1. GLS-010 showed statistically significant anti-tumor effects in the MC38 model in human PD-1 knock-in mice. The RO rate on in the low-, moderate-, and high-dose groups were 64.50%-48.53% in CD3^+^T, 58.87%-40.12% in CD8^+^T, and 66.26%-49.07% in CD4^+^T, respectively. With the increasing dose from 2 mg/kg to 18 mg/kg, the systemic exposure level of GLS-010 (AUC_0-last_) and C_0_ increased proportionally, while the proportion of AUC_0-last_ was higher than the proportion of the increase in the dose.

**Conclusions:**

As a fully human anti-PD-1 monoclonal antibody, GLS-010 has a high affinity to PD-1 and shows potent anti-tumor effects *in vivo* and *in vitro*. The results support that GLS-010 could be investigated in clinical trials in tumor patients.

## Introduction

Innate immunity can recognize cancer cells as non-self and destroy them, but the inflammatory cytokines secreted by immune cells can also activate cancer cells (1–4). In addition, the cancer cells can evade the immune attacks by selecting mutated clones that are resistant to immune effectors and/or by the induction of an immunosuppressive microenvironment that allows the tumor cells to evade immune surveillance (1, 3, 4). In the process of immune escape, immunosuppressive cells (Treg cells, myeloid-derived suppressor cells, and tumor-associated macrophages and neutrophils) accumulate within the tumor and inactivate the cytotoxic T cells (5–10). Immune escape also involves the activation of immune checkpoints that inhibit the activation of effector T cells, including programmed cell death (PD)-1/PD ligand (PD-L1), galectin-9/TIM-3, IDO1, LAG-3, and CTLA-4 (11–13).

Blocking those checkpoints is considered a breakthrough to inhibit tumor immune escape and enhance the immune system to destroy the cancer cells (11–13). Several checkpoint inhibitors were designed against PD-1 or PD-L1 to block the activation of PD-1 (11–13). The PD-1/PD-L1 interactions inhibit T cell receptor signal transduction and attenuate cytokine production, T-cell survival, and proliferation (14). Molecular mechanisms involve the suppression of T cell activation and function through the recruitment of the phosphatases SH2 domain-containing tyrosine phosphatase 1 (SHP1), SHP2 and serine/threonine protein phosphatase 2A. These phosphatases dephosphorylate several major signaling nodes essential for co-stimulation of T cells (14). Therefore, therapeutic antibodies blocking PD-1 restore anti-tumor immunity and lead to durable tumor regression and prolonged survival in some patients (15, 16). Seven PD-1 antibodies have been approved so far with indications in melanoma, lung cancer, renal cell carcinoma, urothelial cancer, Hodgkin lymphoma, head and neck cancer, colorectal cancer, esophageal cancer, malignant mesothelioma, and liver cancer (17–22). The first therapeutic antibodies produced were of murine origin, followed by chimeric antibodies (i.e., containing the murine variable domain and human constant domain), humanized antibodies (i.e., human antibodies with a murine hypervariable loop), and then fully human antibodies. Although antibody drugs are generally well-tolerated, fully human antibodies could be associated with fewer adverse events and immunogenicity than chimeric and humanized antibodies (23–25).

GLS-010 (zimberelimab) is a novel fully human anti-PD-1 monoclonal immunoglobulin G4 (IgG4) developed from the OmniRat transgenic platform (26–28). This paper reports the preclinical data of GLS-010, including affinity, competitive blocking capability, T cell activation effect, Fc-mediated effector functions, preliminary anti-tumor activity, receptor occupancy (RO), and pharmacokinetics (PK). These promising results provide a steppingstone for ongoing clinical development in new drugs in oncology.

## Materials and Methods

### Characteristics of GLS-010

GLS-010 is protected by patent #WO 2017/025051 A1 (29). It is manufactured at a concentration of 30 mg/mL and >99% purity. It is stable for 3 years at a storage temperature of 2-8°C.

### Kinetic Affinity of the GLS-010 to Human PD-1 Protein by Surface Plasmon Resonance

The dynamic affinity of the anti-PD-1 antibody to human PD-1 was assessed by the SPR technique. The antibodies, including GLS-010 and nivolumab, were diluted to 2 μg/mL with 1× PBS/0.01% Tween 20 and injected (at 30 μL/min) to the channel of a sensor chip pre-coated with 10 μg/mL of protein A. The chip was rotated by 90° and rinsed by running buffer until the baseline was stable. The analyte WBP305.hPro1.ECD.His (containing the His-labeled extracellular fusion domain of human PD-1 protein) was diluted by the same buffer to different concentrations (0.625, 1.25, 2.5, 5, and 10 nmol/L). The buffer and the five concentrations of analytes were eluted through the chip channel successively, at a flow rate of 100 μL/min. The association phase for the samples was 240 s, followed by the dissociation phase of 600 s. The data were fitted by the Langmuir analysis 1:1 model to obtain the association rate constant (k_a_), dissociation rate constant (k_d_), and affinity constant (KD).

### Binding of the GLS-010 to Cell Surface PD-1 by FACS

FACS was used to measure the affinity of the anti-PD-1 antibody to the cellular surface PD-1 protein. The WBP305.CHO-S.hPro1.C6 cells (an engineered cell line expressing the human PD-1 protein) or activated CD4^+^ T cells were incubated with PD-1 antibodies (2-fold serially diluted from 66.7 nmol/L to 0.01 nmol/L) at 4°C for 1 h. After the cells were washed, a PE-labeled goat-anti-human antibody was used to measure the binding of the anti-PD-1 antibodies to the cells. Nivolumab was used as a positive control, and non-transfected cells were used as a negative control. The mean fluorescence intensity (MFI) of the cells was measured by flow cytometer and quantitatively analyzed by the FlowJo software (BD Biosciences, Franklin Lake, NJ, USA).

### Affinity of GLS-010 to Human, Cynomolgus Monkeys, and Mouse PD-1 by ELISA

The affinity of the antibody to human- (human PD-1.ECD.His), cynomolgus monkeys- (cynomolgus PD-1.ECD.His), and mouse- (mouse PD-1.ECD.His) derived PD-1 was assessed by ELISA. The ELISA plates were pre-coated with human PD-1.ECD.His, cynomolgus PD-1.ECD.His, and mouse PD-1.ECD.His. The plates were blocked with 2% BSA-containing PBS, and 100 μL of testing antibody (GLS-010, nivolumab, isotype control, and negative control) at 6.67 nmol/L was added. After incubation and rinsing, HRP-labeled goat anti-human IgG antibody was added, incubated at room temperature, rinsed, and developed with TMB substrate for 10 min. The reaction was stopped with 2 M HCl, and the absorbance was read at 450 nm using a microplate spectrophotometer within 15 min.

### Binding of GLS-010 to Human PD-1 Family Proteins by ELISA

Plates were pre-coated with 1 μg/mL of human PD-1.ECD.mFc, CD28.ECD.mFc, CTLA-4.ECD.His, or ICOS.ECD.mFc at 4°C overnight. After 1 h of blocking, testing antibodies were added to the plates at 66.7 nmol/L. The plate was incubated at ambient temperature for 2 h. The binding of the antibodies to the immobilized protein was detected by the HRP-labeled goat anti-human IgG antibody. The color was developed by dispensing 100 μL of TMB substrate and then stopped using 100 μL of 2 N HCl. The absorbance was read at 450 nmol/L and 540 nmol/L using a microplate spectrophotometer.

### Evaluating Human PD-1/PD-L1 Blocking Capability of GLS-010 by FACS

FACS was used to evaluate the competitive blocking capability of the testing antibody to PD-L1 against PD-1 on the cell surface. WBP305.CHO-S.hPro1.C6 was incubated with anti-PD-1 antibodies (2-fold serially diluted from 66.7 to 0.01 nmol/L) at 4°C for 1 h. A constant concentration of PD-L1.ECD.mFc (containing mouse mFc-labeled extracellular fusion domain of human PD-L1) was added. The MFI of the cells was measured by flow cytometry and quantitatively analyzed by the FlowJo software (BD Biosciences, Franklin Lake, NJ, USA).

### Evaluating Human PD-1/PD-L2 Blocking Capability of GLS-010 by ELISA

ELISA was used to evaluate the competitive blocking capability of the testing antibody to PD-L2 against PD-1. The ELISA plates were pre-coated with human PD-1-mFc (containing mouse Fc-labeled extracellular fusion domain of human PD-1 protein), incubated at 4°C overnight. After 1 h of blocking by PBS containing 2% BSA the next day, constant concentration of human PD-L2.ECD.His (5 μg/mL, containing His-labeled extracellular fusion domain of human PD-L2) and testing antibodies (3-fold serial dilution from 66.7 nmol/L to 0.001 nmol/L) were pre-mixed and added to the plates and incubated for 1 h at room temperature. HRP-labeled goat anti-His antibody was added after rinsing, incubated at room temperature, rinsed, and developed with TMB substrate for 10 min. The reaction was stopped using 2 M HCl, and the absorbance was read at 450 nm using a microplate spectrophotometer within 15 min.

### Activation Effect of GLS-010 on the Proliferation of CD4^+^ T Cells by Mixed Lymphocyte Reaction

The experiments were performed according to the “Methods for treating cancer using anti-PD-1 antibodies” (patent #US-9084776-B2). For allogeneic MLR, DCs and CD4^+^ T cells from different donors were mixed at a ratio of 1:10 DC:T cells in the presence or absence of the study antibodies. For autologous MLR, PBMCs were treated with CMV pp65 peptide and a low dose of rhIL-2 (20 U/mL) for 5 days before CD4^+^ T cell isolation. In the meanwhile, DCs were generated by inducing monocytes from the same donor’s PBMC. After 5 days’ culture, the DCs were harvested and pulsed with CMV pp65 peptide for one hour, and then co-cultured with CMV pre-treated CD4^+^ T cells at a ratio of 1:10 DC:T in the presence or absence of the study antibodies. MLR was set up in 96-well round-bottom plates using a complete RPMI-1640 medium. CD4^+^ T cells, various concentrations of antibodies (166.75 nM, 66.7 nM, 6.67 nM, 0.667 nM, 0.0667 nM and 0.00667 nM), and DCs were incubated at 37°C, 5% CO_2_ for five days. The supernatant was harvested for IFN-γ production detection, and the cells were harvested to measure proliferation by ^3^H-TDR.

### Antibody-Dependent Cell-Mediated Cytotoxicity of Anti-PD-1 Antibody

Antibody-dependent cell-mediated cytotoxicity (ADCC) refers to the killing of target cells by natural killer (NK) cells mediated by the binding of the Fab segment of specific antibody to target cells and the binding of the Fc segment to CD16 on the surface of NK cells. The activated CD4^+^ T cells(2×10^4^ cells/well) and effector cells (PBMC,1×10^6^ cells/well) were mixed at a ratio of 1:50 and incubated with various concentrations of testing anti-PD-1 antibodies at 37°C with 5% CO_2_ for 5 h. Then, the lysis of the target cells was assessed by the LDH cytotoxicity kit. The BT474 cells (American Type Culture Collection (ATCC), Manassas, VA, USA) treated with trastuzumab were used as a positive control. The percentage of target cell lysis was calculated using the formula: % of lysis = (OD_sample_ – OD_PBMC_ – OD_target background_)/(OD_target lysis_ – OD_target background_) * 100%.

### Complement-Dependent Cytotoxicity of Anti-PD-1 Antibody

Complement-dependent cytotoxicity (CDC) refers to the cell lysis effects induced by the membrane attack complex formed by the binding of specific antibodies to the corresponding cell surface antigens, which triggers the canonical complement activation pathway. The activated CD4^+^ T cells (2×10^4^ cells/well) were used as the target cells, mixed with various concentrations of anti-PD-1 antibodies. Then normal human serum complements (A112, Quidel Corp., San Diego, CA, USA) diluted to certain concentrations were added. The cells were cultured at 37°C with 5% CO_2_ for 3 h. The cell lysis was assessed by the CellTiter-Glo cell viability kit. The target Ramos cells (American Type Culture Collection (ATCC), Manassas, VA, USA) were treated with rituximab (synthesized according to the C2B8 sequences described by the Genentech company in the patent US5736137) were used as the positive control. The percentage of target lysis was calculated as (1 – RFU_sample_/RFU_total cells_) * 100%.

### Efficacy Study in MC38 Tumor Mouse Models

The animal experiments were approved by the animal use committee of Crown Bioscience. During the experiments, the raising and using of the animals strictly abided by the AAALAC guidelines. The murine colon carcinoma MC38 cells were subcutaneously inoculated to the right flank of the hPD-1 knock-in mice (1×10^6^/mouse). When the tumor size reached amount 150 mm^3^ (which was considered day 0), the mice were randomly divided into four groups: IgG isotype control group, 10 mg/kg GLS-010 group, 20 mg/kg GLS-010 group, and positive control group (treated with 20 mg/kg pembrolizumab), with eight mice in each group. After treatment, the efficacy assessment was performed by evaluating the tumor volume and tumor growth inhibitory rate (T/C% and TGI%). Safety assessment was performed by evaluating the bodyweight change and death of the mice.

### Receptor Occupancy Assay

Forty-eight cynomolgus monkeys were selected and randomly divided into four groups according to the doses of GLS-010: control group (0 mg/kg), low dose group (5 mg/kg), moderate dose group (25 mg/kg), and high dose group (100 mg/kg). GLS-010 was administered once per week for 26 continuous weeks, followed by a recovery phase of 4 weeks.

The blood samples were collected at 1 ± 0.5 h after the last dosing on D50 for all the animals. In addition, the blood samples of the recovery phase were collected in the last week in the recovery phase (on D206 and D207). The blood samples were stained, washed, fixed, and measured about the occupancy of the receptors on the surface of the activated T cells by flow cytometry.

### Pharmacokinetics

Nine male and nine female cynomolgus monkeys were selected and randomly divided into the low, moderate, and high dose groups and received an intravenous injection of 2, 6, and 18 mg/kg GLS-010, respectively. The samples for PK assessment were collected before (0) and at 0.25, 0.5, 1, 2, 4, 8, 24, 48, 72, 144, 312, 480, 648, and 816 h after dosing, respectively. The samples for ADA assessment were collected at -168, 0 (before dosing), 312, 648, and 984 h. WinNonlin Version 6.2.1 software (Certara, Princeton, NJ, USA) was used to process the serum levels of GLS-010 with the non-compartment model. The PK parameters were calculated by the linear and logarithmic trapezoidal methods.

### Statistical Analysis

Statistical differences between groups were evaluated using one-way analysis of variances (ANOVA) followed by the Dunnett’s test. All the data were analyzed using SPSS 19.0 (IBM, Armonk, NY, USA).

## Results

### GLS-010 Has High Affinity and Binding Specificity to PD-1

**Figure 1:**
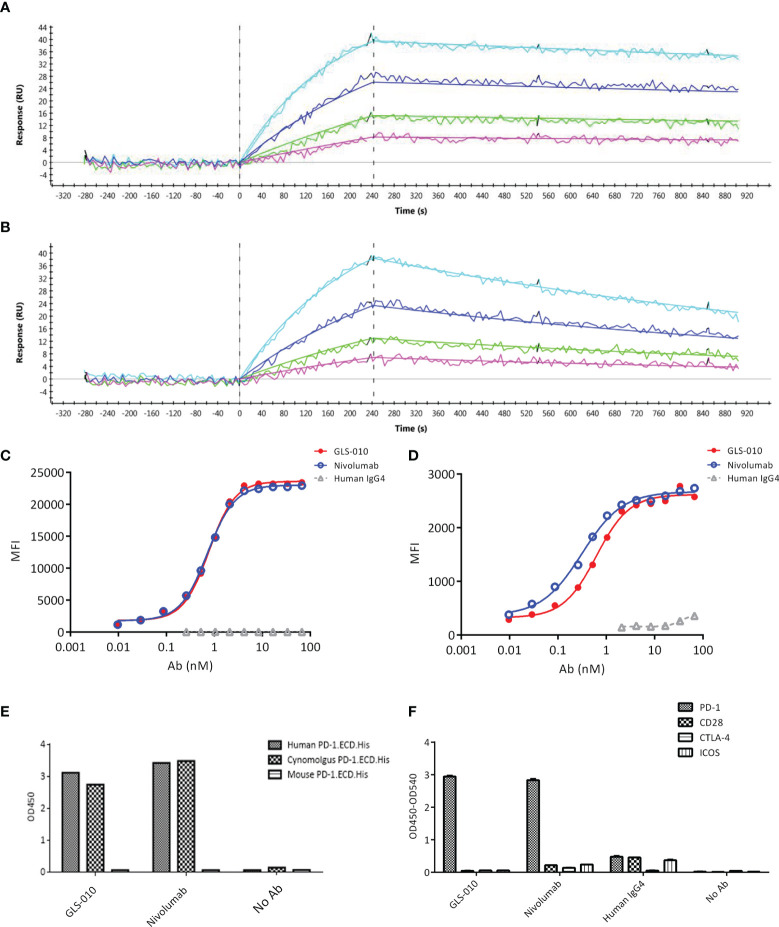
Affinity and binding specificity of GLS-010 to PD-1 by surface plasmon resonance (SPR) and cell-based assays. Association and dissociation of GLS-010 **(A)** and nivolumab **(B)** to hPD-1 by SPR. The concentration, from low to high, was 1.25, 2.5, 5, and 10 nM. The binding affinity of GLS-010 and nivolumab to CHO cell **(C)** and primary CD4^+^T cell **(D)** expressing hPD-1. **(E)** Affinity of GLS-010 and nivolumab to human-, cynomolgus monkeys-, and mouse-derived PD-1 by ELISA. **(F)** GLS-010 and nivolumab bind to hPD-1 but not to other CD28 homologous proteins, including CD28, CTLA-4, and ICOS.

The association and dissociation diagrams of GLS-010 to the human PD-1 protein were analyzed; it was found that GLS-010 had a high affinity to the PD-1 protein, and the KD was 1.75×10^-10^ M, while the KD of nivolumab was 1.16×10^-9^ M (**Table 1** and **Figures 1A, B**). GLS-010 had a relatively high concentration-dependent binding ability to both human PD-1-engineered CHO-S cells and activated human CD4^+^ T cells, for which the EC_50_ was 0.77 nmol/L and 0.62 nmol/L, respectively (**Figures 1C, D**). Thus, GLS-010 could bind to human PD-1.ECD.His and cynomolgus PD-1.ECD.His, but not to mouse PD-1.ECD.His (**Figure 1E**). GLS-010 and nivolumab could specifically bind to the human PD-1 protein but not to the other proteins of the family (such as CD28, CTLA-4, and ICOS) (**Figure 1F**). Therefore, the results showed that GLS-010 had a high affinity and binding specificity to PD-1 without affecting the other members of the same family, indicating the potential of GLS-010 as a targeted therapy against PD-1.

**Table 1:** Binding affinity of GLS-010 to hPD-1 protein by SPR.

Analyte	Ligand	ka (1/Ms)	kd (1/s)	KD (M)	R_max_ (RU)	Chi^2^ (RU)
**Human PD-1.ECD.His**	GLS-010	1.12E+06	1.96E-04	1.75E-10	54.14	2.58
	Nivolumab	7.76E+05	8.97E-04	1.16E-09	68.9	2.2

SPR, surface plasmon resonance; k_a_, association rate constant; k_d_, dissociation rate constant; KD, affinity constant; R_max_, maximum observed response; Chi^2^, goodness of fit indicator.

### GLS-10 Blocks the Binding of PD-L1/2 to PD-1

**Figure 2:**
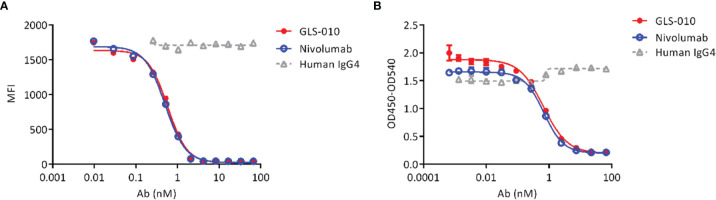
GLS-010 blocks the binding of PD-1 ligands to PD-1. **(A)** Blocking the binding of PD-L1 with PD-1 by GLS-010 and nivolumab in CHO-S cells by FACS. **(B)** Blocking the binding of PD-L2 with PD-1 by GLS-010 and nivolumab by ELISA.

Both GLS-010 and the positive control nivolumab effectively blocked the binding of PD-L1 to cell-surface PD-1 in CHO-S cells. The IC_50_ of GLS-010 was 0.58 nmol/L (**Figure 2A**). In addition, both GLS-010 and the positive control nivolumab could effectively block the binding of PD-L2 to PD-1. The IC_50_ of GLS-010 was 0.67 nmol/L (**Figure 2B**).

### GLS-010 Promotes CD4^+^ T Cell Proliferation

**Figure 3:**
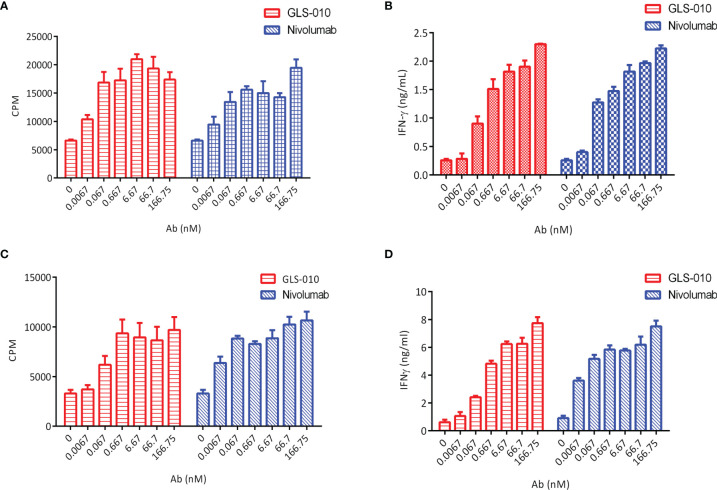
Functional activity of GLS-010 in cell-based bioassays. **(A)** Induction of Human CD4^+^ T lymphocytes using allogeneic mixed lymphocyte reaction (MLR). **(B)** Induction of IFN-γ activation of human CD4^+^ T cells using allogeneic MLR. **(C)** Induction of Human CD4^+^ T lymphocytes using autologous MLR. **(D)** Induction of IFN-γ activation of human CD4^+^ T cells using autologous MLR.

The findings of the allogeneic and autologous MLR showed that GLS-010 could enhance the secretion of IFN-γ by CD4^+^ T cells and promote CD4^+^ T cells proliferation. In addition, the activation of CD4^+^ T cells by the anti-PD-1 antibody was concentration-dependent (**Figures 3A–D**).

**Figure 4:**
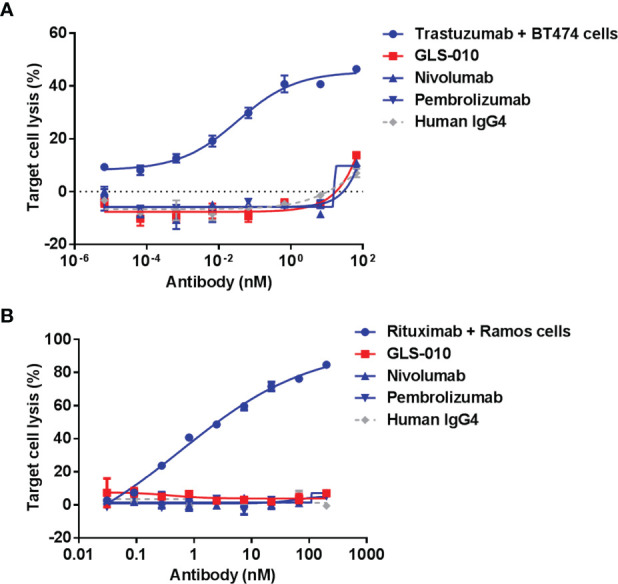
Antibody-dependent cell-mediated cytotoxicity (ADCC) and complement-dependent cytotoxicity (CDC) of anti-PD-1 antibodies. **(A)** ADCC of GLS-010 and anti-PD-1 control antibodies. **(B)** CDC of GLS-010 and anti-PD-1 control antibodies.

The effector cells (PBMC) showed no cytotoxic effects on activated CD4^+^ T cells in the presence of GLS-010 or positive control antibodies, and thus GLS-010 showed no evident ADCC effects, suggesting that GLS-010 could not mediate lysis of PD-1-positive lymphocytes by NK cells (**Figure 4A**). In addition, no evident lysis of activated CD4^+^ T cells was found in the presence of GLS-010 or positive control antibodies, and normal human serum complements, suggesting that GLS-010 had no evident CDC effects (**Figure 4B**).

### GLS-010 Displays Anti-Tumor Effects in Mouse Xenograft Models

**Figure 5:**
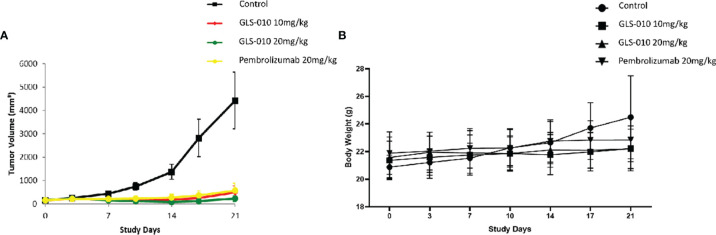
The efficacy of GLS-010 was evaluated in hPD-1 knock-in model with subcutaneous MC38 mouse colon adenocarcinoma. The dosing schedule was BIW*3. **(A)** Tumor growth inhibition (MC38) in hPD-1 knock-in mice treated with GLS-010. **(B)** Effect of GLS-010 on mouse body weight.

In this study, the GLS-010 injection (10 and 20 mg/kg) showed statistically significant anti-tumor effects in the human PD-1 knock-in mouse model of MC38 tumors (**Figure 5A**). In addition, the effects of lower and equal concentrations of GLS-010 injection were comparable to the positive control (pembrolizumab) group, while the TGI% of these two concentrations of GLS-010 injection were both higher, numerically than pembrolizumab (**Table 2** and **Figures 5A, B**).

**Table 2:** Tumor-suppressing effects of GLS-010 in hPD-1KI MC38 tumor-bearing mice.

Treatment	Tumor volume on D0^a^ (mm^3^)	Tumor volume on D10^a^ (mm^3^)	Tumor volume on D14^a^ (mm^3^)	TGI^b^ (%)	T/C^b^ (%)	T-C (days)	P^bc^
Isotype control (20 mg/kg)	149.23 ± 10.65	741.36 ± 164.62	1367.12 ± 316.52	–	–	–	–
GLS-010 (10 mg/kg)	150.14 ± 10.33	137.02 ± 54.08	172.71 ± 96.99	98 (102)	13 (18)	>8	<0.05 (<0.05)
GLS-010 (20 mg/kg)	149.56 ± 10.24	112.55 ± 21.72	76.56 ± 31.76	106 (106)	6 (15)	>8	<0.05 (<0.05)
Pembrolizumab (20 mg/kg)	149.41 ± 9.84	232.37 ± 80.05	273.95 ± 130.64	90 (86)	20 (31)	>8	>0.05 (>0.05)

a: Data are described as means ± SD;

b: The P values for the TGI and T/C on D10 are shown in the brackets;

c: The tumor volumes among different treatment groups are analyzed by one-way analysis of variances (one-way ANOVA); the volumes in the groups 2-3 were significantly different from group 1 (P < 0.05), but the differences among the groups 2-4 were not statistically significant (P > 0.05).

TGI, tumor growth inhibition rate; T/C, tumor volume.

### GLS-010 Displays Long-Term Effects in Cynomolgus Monkeys, Without Differences Between Males and Females

**Figure 6:**
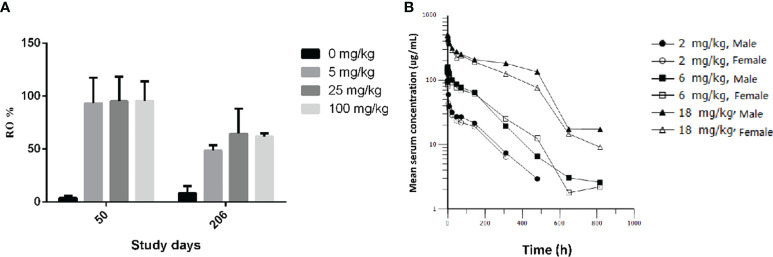
PD-1 receptor occupancy and pharmacokinetic profiles of GLS-010 in cynomolgus macaques. **(A)** PD-1 receptor occupancy rate of activated CD3^+^ T cells of different doses of GLS-010 in cynomolgus macaques on day 50 and day 206. **(B)** Pharmacokinetic profiles of different doses of GLS-010 divided by sex in cynomolgus macaques.

After an intravenous injection of GLS-010, the RO rate on the D50 were saturated in the cynomolgus monkeys in the low-, moderate-, and high-dose groups, compared with the control group (CD3^+^T: 95.54% to 93.25%; CD8^+^T: 106.03% to 92.96%; and CD4^+^T: 119.72% to 77.98%). The RO rate on D206 (the last week of the recovery phase) in the low-, moderate-, and high-dose groups were lower than the D50 (CD3^+^T: 64.50% to 48.53%; CD8^+^T: 58.87% to 40.12%; CD4^+^T: 66.26% to 49.07%) but were higher than in the control group. In the recovery phase, GLS-010 was detected in the low-, moderate-, and high-dose groups on D190, D197, D204, and D211 (**Table 3** and **Figure 6A**), suggesting that GLS-010 could exert long-term effects.

**Table 3:** The average RO rate of CD3^+^,CD4^+^, and CD8^+^ T cells in monkeys following Iv of GLS-010 at different doses (%, Mean ± SD).

Time (Day)	T cell	n	*Iv* GLS-010	*Iv* GLS-010	*Iv* GLS-010	*Iv* GLS-010
			0 mg/kg	5 mg/kg	25 mg/kg	100 mg/kg
50	CD3^+^	12	3.99 ± 1.83	93.25 ± 24.66	95.19 ± 23.20	95.54 ± 18.53
206		4	8.25 ± 6.75	48.53 ± 5.29	64.50 ± 23.73	61.87 ± 2.92
50	CD8^+^	12	5.81 ± 3.55	77.98 ± 24.20^c^	119.72 ± 64.93	114.70 ± 49.70^b^
206		4	4.39 ± 2.33	49.07 ± 13.99	66.26 ± 10.14	60.99 ± 6.81
50	CD4^+^	12	4.03 ± 2.49	106.03 ± 34.68	97.76 ± 17.13	92.96 ± 16.43
206		4	3.10 ± 1.30	41.48 ± 6.08	40.12 ± 2.63^d^	58.87 ± 5.28^b^

b,bb, bbb: P < 0.05, 0.01, 0.001 vs 5 mg/kg ;c,cc, ccc:P < 0.05, 0.01, 0.001 vs 25 mg/kg;d,dd, ddd:P < 0.05, 0.01, 0.001 vs 100 mg/kg.

After a single intravenous injection of 2, 6, and 18 mg/kg GLS-010, the curves of the individual and average serum GLS-010 concentrations were not different between male and female animals (**Table 4** and **Figure 6B**). With the increase of dose from 2 to 18 mg/kg, the systemic exposure level of GLS-010 (AUC_0-last_) and C_0_ increased proportionally. In contrast, the proportion of the increase in the exposure to GLS-010 (AUC_0-last_ and C_0_) was higher than the proportion of the increase in the dose (**Table 3**).

**Table 4:** PK parameters of GLS-010 after single vd administrations of 2, 6, and 18 mg/kg in cynomolgus macaques.

PK parameters	2 mg/kg	6 mg/kg	18 mg/kg
**C_0_ (μg/mL)**	103 ± 23.7 (23.0%)	157 ± 18.7 (11.9%)	508 ± 48.0 (9.46%)
**T_1/2_ (h)**	111 ± 23.7 (30.5%)	115 ± 32.8 (28.5%)	129 ± 17.0 (13.1%)
**Vss (mL/kg)**	48.4 ± 7.48 (15.5%)	49.4 ± 6.49 (13.1%)	46.3 ± 5.49 (11.8%)
**Cl (mL/h/kg)**	0.288 ± 0.0373 (13.0%)	0.278 ± 0.0308 (11.1%)	0.183 ± 0.0293 (16.0%)
**T_last_ (h)**	396 ± 141 (35.5%)	704 ± 203 (28.9%)	816 ± 0.00
AUC_0-last_ (h*μg/mL)	6300 ± 1320 (21.0%)	21300 ± 2570 (12.1%)	98100 ± 16300 (16.6%)
AUC_0-inf_ (h*μg/mL)	7060 ± 1020 (14.5%)	21800 ± 2310 (10.6%)	101000 ± 16700 (16.6%)
**MRT_0-last_ (h)**	126 ± 23.3 (18.4%)	164 ± 31.2 (19.0%)	236 ± 14.8 (6.27%)
**MRT_0-inf_ (h)**	170 ± 29.7 (17.5%)	180 ± 29.4 (16.4%)	255 ± 17.4 (6.82%)
**AUC_0-inf_/AUC_0-last_ (%)**	113 ± 11.8 (10.4%)	103 ± 2.11 (2.06%)	103 ± 0.940 (0.916%)

C_0_, initial drug concentration; T_1/2_, half-life; Vss, apparent volume of distribution in the steady-state; Cl, clearance; T_last_, the last time; AUC, area under the curve; MRT, mean residence time.

In the 2 mg/kg group, the positive rate of ADA was 33.3%, of which the ADA-positive rate in female animals was 40%, and the antibody titer was higher in female animals than in males. In the 6 mg/kg group, the positive rate of ADA was 20%, and the antibody titer was slightly higher in male animals. In the 18 mg/kg group, the positive rate of ADA was 10%.

GLS-010 was administered by single intravenous infusion to cynomolgus monkeys at doses of 0, 100, 300 and 1000 mg/kg, respectively. A slight decrease in body weight, leukocytes, and neutrophils were observed only in 1000 mg/kg group for 14 days (**Supplemental Data 1**). Therefore, it is considered that the maximum tolerated dose of GLS-010 injection administered by single intravenous infusion is not reached.

No abnormal manifestation was observed in the animals in the 26 continuous weeks dosing study, including clinical manifestations, local irritation, body weight, food intake, body temperature, ophthalmic examination, blood routine, coagulation, serum biochemical parameters, urine examination, blood pressure, electrocardiogram, respiratory monitoring, immune analysis (serum IL-2, 4, 5, 6, 10, and 13; TNF-α; IgA and IgM; complement C3c and C4. **Supplemental Data 2**).

## Discussion

This study aimed to report the affinity, competitive blocking capability, T cell activation effect, Fc-mediated effector functions, preliminary anti-tumor activity, RO, and PK of GLS-010, a novel fully human anti-PD-1 monoclonal IgG4 (26, 27). GLS-010 uses the human IgG4 isoform as the constant region backbone, with the S228P mutation preventing Fab arm exchange, and has the same heavy chain constant region sequence as Nivolumab and Pembrolizumab. Our results strongly suggest that GLS-010 has a high affinity to PD-1 with a stimulatory effect on CD4^+^ T cells *in vitro* and promising *in vivo* anti-tumor effects in mouse xenograft models. The findings of this study indicate the promising preclinical evaluation of GLS-010 as a checkpoint inhibitor and support that GLS-010 can be investigated in clinical trials.

GLS-010 has high affinity and specificity to PD-1, similar to nivolumab, the first approved anti-PD-1 antibody for cancer treatment (30–32). Like nivolumab and pembrolizumab, GLS-010 is also an IgG4 monoclonal antibody. As nivolumab, GLS-010 is a fully human antibody, while pembrolizumab is a humanized one (30–32). The possible advantage of GLS-010 compared with the existing anti-PD-1 antibodies (pembrolizumab) is its fully human nature (24, 25). In addition, GLS-010 had a higher binding affinity than nivolumab (KD: 1.75E-10 *vs.* 1.16E-09). Still, GLS-010 displayed high RO over time, suggesting that it could have a long-lasting effect. In addition, a recent dose-escalation and expansion (phase Ia/Ib) study of GLS-010 showed its acceptable safety profile and favorable clinical response, and the dose of 240 mg Q2W was an optimal recommended dose as monotherapy (16).

A functional assessment of GLS-010 on T cell responses was performed. The results showed that GLS-010 enhanced IFN-γ production by CD4^+^ T cells and promoted the proliferation of CD4^+^ T cells. In addition, the ADCC and CDC experiment results showed that GLS-010, like Nivolumab and Pembrolizumab, did not show ADCC and CDC activity for PD-1-positive CD4^+^ T cells.

After a single injection of different doses, the PK results also showed that GLS-010 could be used in clinical trials. With the increasing doses, the increase in the exposure to GLS-010 (AUC_0-last_ and C_0_) was higher than the proportion of the increase in the dose, as observed in a phase Ia study (27). Nevertheless, more studies are still needed to investigate the dose-response relationship of GLS-010 further. After intravenous infusion, the RO rate could reach saturation within a short time. A phase Ia study showed a RO of >80% in humans (27). GLS-010 could still be detected in the all dosing groups in the recovery period, i.e., after 26 weeks, suggesting that GLS-010 could have long-term effects, but it needs to be validated in humans. In addition, how these long-term RO would affect the dosage still have to be determined.

The preliminary clinical efficacy results showed that GLS-010 had significant anti-tumor effects in mice models of MC38 tumors, comparable with pembrolizumab (20–22, 30, 32). These findings demonstrate that GLS-010 is a promising agent for the treatment of cancer patients. *In vitro*, the blockade of PD-1 by GLS-010 results in the potent activation of T cell activity. Besides its promising preclinical results, GLS-010, as a novel fully human anti-PD-1 monoclonal IgG4 antibody (26, 27), should address immune reaction issues found with non-fully human antibodies. The efficacy and safety of GLS-010 will be further confirmed in clinical trials.

Anti-PD-1 antibodies target PD-1 with high affinity and specificity. The binding of anti-PD-1 antibodies to PD-1 prevents the interaction of PD-1 with PD-L1/2, preventing the activation of the receptor. Normally, PD-1 blocks signaling in T cells through the recruitment of SHP-2, which dephosphorylated the antigen receptor expressed by the T cells, leading to immune tolerance (30, 33). GLS-010 is a novel antibody that has the same target as other anti-PD-1 antibody-based therapies. Therefore, we hypothesize that the mechanisms of GLS-010 are the same as other anti-PD-1 antibodies, and more studies about the mechanisms are needed in the future.

In conclusion, as a fully human anti-PD-1 monoclonal antibody [from the OmniRat™ platform (28)], GLS-010 has a high affinity to PD-1 and shows potent *in vitro* and *in vivo* anti-tumor effects. Our results support that GLS-010 is a good candidate for clinical trials in tumor patients. Furthermore, it is being studied in clinical trials (ClinicalTrials.gov #NCT03655483, NCT03972722, and NCT03713905) and might display promising clinical results.

## Data Availability Statement

The original contributions presented in the study are included in the article/**Supplementary Material**. Further inquiries can be directed to the corresponding author.

## Ethics Statement

The animal study was reviewed and approved by Institutional Animal Care and Use Committee (IACUC) of WuXi AppTec (Suzhou) Co., Ltd.

## Author Contributions

All authors contributed to the study conception and design. Data collection and analysis were performed by BL, BY, and YZ. The first draft of the manuscript was written by BL. HW, FY, SW, and JZ helped draft and critically revised the manuscript. All authors contributed to the article and approved the submitted version.

## Conflict of Interest

BL, HW, FY, SW, and SY: Employment by Guangzhou Gloria Biosciences Co., Ltd. JZ: President of Guangzhou Gloria Biosciences Co., Ltd. BY and YZ: Employment by WuXi Biologics.

## Publisher’s Note

All claims expressed in this article are solely those of the authors and do not necessarily represent those of their affiliated organizations, or those of the publisher, the editors and the reviewers. Any product that may be evaluated in this article, or claim that may be made by its manufacturer, is not guaranteed or endorsed by the publisher.
