# Supplementary material for: Preclinical Characterization of GLS-010 (Zimberelimab), a Novel Fully Human Anti-PD-1 Therapeutic Monoclonal Antibody for Cancer
Source: Front Oncol. 2021 Sep 15;11:736955. doi: 10.3389/fonc.2021.736955 (PMC8479189; doi:10.3389/fonc.2021.736955)
Supplement: Supplementary file 1 [file DataSheet_1.pdf]

Appendix 7 Individual Hematology

Appendix 7 Individual Hematology

Key Page

Measurement Descriptions

Headings Used	Description
WBC	Leukocyte count
RBC	Erythrocyte count
HGB	Hemoglobin
HCT	Hematocrit
MCV	Mean Corpuscular Volume
MCH	Mean Corpuscular Hemoglobin
MCHC	Mean Corpuscular Hemoglobin Conc.
RDW	RBC Distribution Width
#RET	Reticulocytes, absolute
#NEUT	Neutrophils, absolute
%NEUT	Neutrophils, percent
#LYMP	Lymphocytes, absolute
%LYMP	Lymphocytes, percent
#MONO	Monocytes, absolute
%MONO	Monocytes, percent
#EOS	Eosinophils, absolute
%EOS	Eosinophils, percent
#BASO	Basophils, absolute
%BASO	Basophils, percent
PLT	Platelet Count
MPV	Mean Platelet Volume

Appendix 7 Individual Hematology (Cont'd)

Sex: Male

10 mg/kg	WBC (x10^3/μL)	WBC (x10^3/μL)	WBC (x10^3/μL)	RBC (x10^6 /μL)	RBC (x10^6 /μL)	RBC (x10^6 /μL)	HGB (g/dL)	HGB (g/dL)	HGB (g/dL)	HCT (%)	HCT (%)	HCT (%)
	-7	2	15	-7	2	15	-7	2	15	-7	2	15
1001	6.29	9.88	3.94	5.85	6.11	5.80	12.2	12.5	11.6	42.1	42.9	41.0

Appendix 7 Individual Hematology (Cont'd)

Sex: Male

1<br>0<br>mg/kg	MCV<br>(fL)<br>-7	MCV<br>(fL)<br>2	MCV<br>(fL)<br>15	MCH<br>(pg)<br>-7	MCH<br>(pg)<br>2	MCH<br>(pg)<br>15	MCHC<br>(g/dL)<br>-7	MCHC<br>(g/dL)<br>2	MCHC<br>(g/dL)<br>15	RDW<br>(%)<br>-7	RDW<br>(%)<br>2	RDW<br>(%)<br>15
1001	71.8	70.2	70.7	20.8	20.5	20.0	29.0	29.2	28.3	13.8	13.8	13.7

Appendix 7 Individual Hematology (Cont'd)

Sex: Male

1 0 mg/kg	#RET	#RET	#RET	#NEUT	#NEUT	#NEUT	%NEUT	%NEUT	%NEUT	#LYMP	#LYMP	#LYMP
	(x10⁹ /L)	(x10⁹ /L)	(x10⁹ /L)	(x10³/µL)	(x10³/µL)	(x10³/µL)	(%)	(%)	(%)	(x10³/µL)	(x10³/µL)	(x10³/µL)
	-7	2	15	-7	2	15	-7	2	15	-7	2	15
1001	69.4	54.7	78.5	3.11	6.27	1.06	49.5	63.5	26.9	2.76	3.31	2.57

Appendix 7 Individual Hematology (Cont'd)

Sex: Male

1<br>0<br>mg/kg	%LYMP	%LYMP	%LYMP	#MONO	#MONO	#MONO	%MONO	%MONO	%MONO	#EOS	#EOS	#EOS
	(%)	(%)	(%)	(x10³/μL)	(x10³/μL)	(x10³/μL)	(%)	(%)	(%)	(x10³/μL)	(x10³/μL)	(x10³/μL)
	-7	2	15	-7	2	15	-7	2	15	-7	2	15
1001	43.8	33.4	65.1	0.35	0.22	0.24	5.6	2.3	6.1	0.01	0.02	0.01

Appendix 7 Individual Hematology (Cont'd)

Sex: Male

10 mg/kg	%EOS	%EOS	%EOS	#BASO	#BASO	#BASO	%BASO	%BASO	%BASO	PLT	PLT	PLT
	(%)	(%)	(%)	(x10³/μL)	(x10³/μL)	(x10³/μL)	(%)	(%)	(%)	(x10³/μL)	(x10³/μL)	(x10³/μL)
	-7	2	15	-7	2	15	-7	2	15	-7	2	15
1001	0.1	0.2	0.3	0.01	0.01	0.01	0.1	0.1	0.3	301	356	334

Appendix 7 Individual Hematology (Cont'd)

Sex: Male

1<br>0<br>mg/kg	MPV<br>(fL)<br>-7	MPV<br>(fL)<br>2	MPV<br>(fL)<br>15
1001	8.4	8.1	7.8

Appendix 7 Individual Hematology (Cont'd)

Sex: Male

2<br>100<br>mg/kg	WBC	WBC	WBC	RBC	RBC	RBC	HGB	HGB	HGB	HCT	HCT	HCT
	($x10^3/\mu L$)	($x10^3/\mu L$)	($x10^3/\mu L$)	($x10^6/\mu L$)	($x10^6/\mu L$)	($x10^6/\mu L$)	(g/dL)	(g/dL)	(g/dL)	(%)	(%)	(%)
	-7	2	15	-7	2	15	-7	2	15	-7	2	15
2001	7.16	9.41	4.29	5.66	5.52	5.24	13.8	13.4	12.6	42.6	41.9	39.3

Appendix 7 Individual Hematology (Cont'd)

Sex: Male

2<br>100<br>mg/kg	MCV	MCV	MCV	MCH	MCH	MCH	MCHC	MCHC	MCHC	RDW	RDW	RDW
	(fL)	(fL)	(fL)	(pg)	(pg)	(pg)	(g/dL)	(g/dL)	(g/dL)	(%)	(%)	(%)
	-7	2	15	-7	2	15	-7	2	15	-7	2	15
2001	75.3	76.0	75.0	24.4	24.3	24.1	32.4	32.0	32.1	12.6	12.6	12.5

Appendix 7 Individual Hematology (Cont'd)

Sex: Male

2<br>100<br>mg/kg	#RET	#RET	#RET	#NEUT	#NEUT	#NEUT	%NEUT	%NEUT	%NEUT	#LYMP	#LYMP	#LYMP
	($x10^9$/L)	($x10^9$/L)	($x10^9$/L)	($x10^3$/µL)	($x10^3$/µL)	($x10^3$/µL)	(%)	(%)	(%)	($x10^3$/µL)	($x10^3$/µL)	($x10^3$/µL)
	-7	2	15	-7	2	15	-7	2	15	-7	2	15
2001	62.8	114.2	81.3	4.30	7.22	1.76	60.0	76.7	40.9	2.53	1.96	2.32

Appendix 7 Individual Hematology (Cont'd)

Sex: Male

2<br>100<br>mg/kg	%LYMP	%LYMP	%LYMP	#MONO	#MONO	#MONO	%MONO	%MONO	%MONO	#EOS	#EOS	#EOS
	(%)	(%)	(%)	(x10³/μL)	(x10³/μL)	(x10³/μL)	(%)	(%)	(%)	(x10³/μL)	(x10³/μL)	(x10³/μL)
	-7	2	15	-7	2	15	-7	2	15	-7	2	15
2001	35.3	20.9	53.9	0.25	0.17	0.15	3.5	1.8	3.6	0.03	0.04	0.02

Appendix 7 Individual Hematology (Cont'd)

Sex: Male

2<br>100<br>mg/kg	%EOS	%EOS	%EOS	#BASO	#BASO	#BASO	%BASO	%BASO	%BASO	PLT	PLT	PLT
	(%)	(%)	(%)	(x10³/µL)	(x10³/µL)	(x10³/µL)	(%)	(%)	(%)	(x10³/µL)	(x10³/µL)	(x10³/µL)
	-7	2	15	-7	2	15	-7	2	15	-7	2	15
2001	0.4	0.4	0.6	0.02	0.00	0.01	0.2	0.0	0.3	246	271	268

Appendix 7 Individual Hematology (Cont'd)

Sex: Male

2<br>100<br>mg/kg	MPV	MPV	MPV
	(fL)	(fL)	(fL)
	-7	2	15
2001	9.1	8.5	8.4

Appendix 7 Individual Hematology (Cont'd)

Sex: Male

3<br>300<br>mg/kg	WBC	WBC	WBC	RBC	RBC	RBC	HGB	HGB	HGB	HCT	HCT	HCT
	($x10^3/\mu L$)	($x10^3/\mu L$)	($x10^3/\mu L$)	($x10^6/\mu L$)	($x10^6/\mu L$)	($x10^6/\mu L$)	(g/dL)	(g/dL)	(g/dL)	(%)	(%)	(%)
	-7	2	15	-7	2	15	-7	2	15	-7	2	15
3001	12.25	5.93	4.88	5.24	5.22	5.19	12.7	12.5	12.4	39.8	39.6	38.6

Appendix 7 Individual Hematology (Cont'd)

Sex: Male

3<br>300<br>mg/kg	MCV	MCV	MCV	MCH	MCH	MCH	MCHC	MCHC	MCHC	RDW	RDW	RDW
	(fL)	(fL)	(fL)	(pg)	(pg)	(pg)	(g/dL)	(g/dL)	(g/dL)	(%)	(%)	(%)
	-7	2	15	-7	2	15	-7	2	15	-7	2	15
3001	76.0	75.9	74.5	24.2	24.0	24.0	31.8	31.7	32.2	13.8	13.8	13.6

Appendix 7 Individual Hematology (Cont'd)

Sex: Male

3<br>300<br>mg/kg	#RET	#RET	#RET	#NEUT	#NEUT	#NEUT	%NEUT	%NEUT	%NEUT	#LYMP	#LYMP	#LYMP
	(x10⁹ /L)	(x10⁹ /L)	(x10⁹ /L)	($x10^3/\mu L$)	($x10^3/\mu L$)	($x10^3/\mu L$)	(%)	(%)	(%)	($x10^3/\mu L$)	($x10^3/\mu L$)	($x10^3/\mu L$)
	-7	2	15	-7	2	15	-7	2	15	-7	2	15
3001	52.6	55.9	62.6	9.89	3.94	1.77	80.7	66.5	36.4	2.13	1.72	2.90

Appendix 7 Individual Hematology (Cont'd)

Sex: Male

3<br>300<br>mg/kg	%LYMP	%LYMP	%LYMP	#MONO	#MONO	#MONO	%MONO	%MONO	%MONO	#EOS	#EOS	#EOS
	(%)	(%)	(%)	(x10³/μL)	(x10³/μL)	(x10³/μL)	(%)	(%)	(%)	(x10³/μL)	(x10³/μL)	(x10³/μL)
	-7	2	15	-7	2	15	-7	2	15	-7	2	15
3001	17.4	29.1	59.4	0.20	0.21	0.15	1.6	3.5	3.1	0.00	0.01	0.02

Appendix 7 Individual Hematology (Cont'd)

Sex: Male

3<br>300<br>mg/kg	%EOS	%EOS	%EOS	#BASO	#BASO	#BASO	%BASO	%BASO	%BASO	PLT	PLT	PLT
	(%)	(%)	(%)	(x10³/μL)	(x10³/μL)	(x10³/μL)	(%)	(%)	(%)	(x10³/μL)	(x10³/μL)	(x10³/μL)
	-7	2	15	-7	2	15	-7	2	15	-7	2	15
3001	0.0	0.2	0.3	0.01	0.01	0.01	0.1	0.1	0.2	404	445	437

Appendix 7 Individual Hematology (Cont'd)

Sex: Male

3<br>300<br>mg/kg	MPV<br>(fL)<br>-7	MPV<br>(fL)<br>2	MPV<br>(fL)<br>15
3001	7.2	7.3	7.0

Appendix 7 Individual Hematology (Cont'd)

Sex: Male

4<br>1000<br>mg/kg	WBC	WBC	WBC	RBC	RBC	RBC	HGB	HGB	HGB	HCT	HCT	HCT
	($x10^3/\mu L$)	($x10^3/\mu L$)	($x10^3/\mu L$)	($x10^6/\mu L$)	($x10^6/\mu L$)	($x10^6/\mu L$)	(g/dL)	(g/dL)	(g/dL)	(%)	(%)	(%)
	-7	2	15	-7	2	15	-7	2	15	-7	2	15
4001	8.23	3.55	3.03	5.57	5.33	5.40	13.0	12.5	12.6	41.7	41.0	42.1

Appendix 7 Individual Hematology (Cont'd)

Sex: Male

4<br>1000<br>mg/kg	MCV	MCV	MCV	MCH	MCH	MCH	MCHC	MCHC	MCHC	RDW	RDW	RDW
	(fL)	(fL)	(fL)	(pg)	(pg)	(pg)	(g/dL)	(g/dL)	(g/dL)	(%)	(%)	(%)
	-7	2	15	-7	2	15	-7	2	15	-7	2	15
4001	74.9	76.9	78.1	23.4	23.5	23.3	31.2	30.5	29.9	14.4	15.2	15.2

Appendix 7 Individual Hematology (Cont'd)

Sex: Male

4<br>1000<br>mg/kg	#RET	#RET	#RET	#NEUT	#NEUT	#NEUT	%NEUT	%NEUT	%NEUT	#LYMP	#LYMP	#LYMP
	($x10^9$ /L)	($x10^9$ /L)	($x10^9$ /L)	($x10^3/\mu L$)	($x10^3/\mu L$)	($x10^3/\mu L$)	(%)	(%)	(%)	($x10^3/\mu L$)	($x10^3/\mu L$)	($x10^3/\mu L$)
	-7	2	15	-7	2	15	-7	2	15	-7	2	15
4001	32.8	38.8	53.8	6.20	1.51	0.54	75.3	42.5	17.9	1.89	1.90	2.35

Appendix 7 Individual Hematology (Cont'd)

Sex: Male

4<br>1000<br>mg/kg	%LYMP	%LYMP	%LYMP	#MONO	#MONO	#MONO	%MONO	%MONO	%MONO	#EOS	#EOS	#EOS
	(%)	(%)	(%)	(x10³/µL)	(x10³/µL)	(x10³/µL)	(%)	(%)	(%)	(x10³/µL)	(x10³/µL)	(x10³/µL)
	-7	2	15	-7	2	15	-7	2	15	-7	2	15
4001	22.9	53.5	77.7	0.10	0.07	0.05	1.3	2.1	1.6	0.00	0.01	0.01

Appendix 7 Individual Hematology (Cont'd)

Sex: Male

4<br>1000<br>mg/kg	%EOS	%EOS	%EOS	#BASO	#BASO	#BASO	%BASO	%BASO	%BASO	PLT	PLT	PLT
	(%)	(%)	(%)	(x10³/μL)	(x10³/μL)	(x10³/μL)	(%)	(%)	(%)	(x10³/μL)	(x10³/μL)	(x10³/μL)
	-7	2	15	-7	2	15	-7	2	15	-7	2	15
4001	0.0	0.4	0.3	0.00	0.01	0.02	0.0	0.2	0.5	465	500	499

Appendix 7 Individual Hematology (Cont'd)

Sex: Male

4<br>1000<br>mg/kg	MPV	MPV	MPV
	(fL)	(fL)	(fL)
	-7	2	15
4001	7.5	8.0	7.5

Appendix 7 Individual Hematology (Cont'd)

Sex: Female

1<br>0<br>mg/kg	WBC	WBC	WBC	RBC	RBC	RBC	HGB	HGB	HGB	HCT	HCT	HCT
	(x10³/μL)	(x10³/μL)	(x10³/μL)	(x10⁶ /μL)	(x10⁶ /μL)	(x10⁶ /μL)	(g/dL)	(g/dL)	(g/dL)	(%)	(%)	(%)
	-7	2	15	-7	2	15	-7	2	15	-7	2	15
1501	7.05	4.34	5.43	5.62	5.42	5.75	12.0	11.4	12.0	41.7	40.1	43.1

Appendix 7 Individual Hematology (Cont'd)

Sex: Female

1 0 mg/kg	MCV	MCV	MCV	MCH	MCH	MCH	MCHC	MCHC	MCHC	RDW	RDW	RDW
	(fL)	(fL)	(fL)	(pg)	(pg)	(pg)	(g/dL)	(g/dL)	(g/dL)	(%)	(%)	(%)
	-7	2	15	-7	2	15	-7	2	15	-7	2	15
1501	74.1	74.0	74.9	21.3	21.0	20.8	28.7	28.3	27.8	12.7	12.7	13.2

Appendix 7 Individual Hematology (Cont'd)

Sex: Female

1<br>0<br>mg/kg	#RET	#RET	#RET	#NEUT	#NEUT	#NEUT	%NEUT	%NEUT	%NEUT	#LYMP	#LYMP	#LYMP
	($x10^9$ /L)	($x10^9$ /L)	($x10^9$ /L)	($x10^3/\mu L$)	($x10^3/\mu L$)	($x10^3/\mu L$)	(%)	(%)	(%)	($x10^3/\mu L$)	($x10^3/\mu L$)	($x10^3/\mu L$)
	-7	2	15	-7	2	15	-7	2	15	-7	2	15
1501	55.7	30.4	79.5	3.83	1.89	1.18	54.3	43.6	21.7	2.70	2.21	3.68

Appendix 7 Individual Hematology (Cont'd)

Sex: Female

1<br>0<br>mg/kg	%LYMP	%LYMP	%LYMP	#MONO	#MONO	#MONO	%MONO	%MONO	%MONO	#EOS	#EOS	#EOS
	(%)	(%)	(%)	(x10³/μL)	(x10³/μL)	(x10³/μL)	(%)	(%)	(%)	(x10³/μL)	(x10³/μL)	(x10³/μL)
	-7	2	15	-7	2	15	-7	2	15	-7	2	15
1501	38.3	51.0	67.8	0.37	0.17	0.46	5.2	4.0	8.5	0.10	0.03	0.05

Appendix 7 Individual Hematology (Cont'd)

Sex: Female

1<br>0<br>mg/kg	%EOS	%EOS	%EOS	#BASO	#BASO	#BASO	%BASO	%BASO	%BASO	PLT	PLT	PLT
	(%)	(%)	(%)	(x10³/μL)	(x10³/μL)	(x10³/μL)	(%)	(%)	(%)	(x10³/μL)	(x10³/μL)	(x10³/μL)
	-7	2	15	-7	2	15	-7	2	15	-7	2	15
1501	1.4	0.8	0.9	0.02	0.01	0.01	0.3	0.1	0.3	338	334	416

Appendix 7 Individual Hematology (Cont'd)

Sex: Female

1<br>0<br>mg/kg	MPV<br>(fL)<br>-7	MPV<br>(fL)<br>2	MPV<br>(fL)<br>15
1501	8.6	8.0	8.6

Appendix 7 Individual Hematology (Cont'd)

Sex: Female

2<br>100<br>mg/kg	WBC	WBC	WBC	RBC	RBC	RBC	HGB	HGB	HGB	HCT	HCT	HCT
	($x10^3/\mu L$)	($x10^3/\mu L$)	($x10^3/\mu L$)	($x10^6$ /μL)	($x10^6$ /μL)	($x10^6$ /μL)	(g/dL)	(g/dL)	(g/dL)	(%)	(%)	(%)
	-7	2	15	-7	2	15	-7	2	15	-7	2	15
2501	8.58	7.71	8.19	5.28	5.25	5.07	13.5	13.4	13.1	41.2	42.3	41.5

Appendix 7 Individual Hematology (Cont'd)

Sex: Female

2<br>100<br>mg/kg	MCV	MCV	MCV	MCH	MCH	MCH	MCHC	MCHC	MCHC	RDW	RDW	RDW
	(fL)	(fL)	(fL)	(pg)	(pg)	(pg)	(g/dL)	(g/dL)	(g/dL)	(%)	(%)	(%)
	-7	2	15	-7	2	15	-7	2	15	-7	2	15
2501	77.9	80.6	81.8	25.6	25.5	25.9	32.8	31.7	31.6	12.5	12.6	12.9

Appendix 7 Individual Hematology (Cont'd)

Sex: Female

2<br>100<br>mg/kg	#RET	#RET	#RET	#NEUT	#NEUT	#NEUT	%NEUT	%NEUT	%NEUT	#LYMP	#LYMP	#LYMP
	(x10⁹ /L)	(x10⁹ /L)	(x10⁹ /L)	(x10³/μL)	(x10³/μL)	(x10³/μL)	(%)	(%)	(%)	(x10³/μL)	(x10³/μL)	(x10³/μL)
	-7	2	15	-7	2	15	-7	2	15	-7	2	15
2501	52.9	68.0	122.4	5.45	4.79	3.76	63.5	62.2	45.8	2.78	2.49	3.93

Appendix 7 Individual Hematology (Cont'd)

Sex: Female

2<br>100<br>mg/kg	%LYMP	%LYMP	%LYMP	#MONO	#MONO	#MONO	%MONO	%MONO	%MONO	#EOS	#EOS	#EOS
	(%)	(%)	(%)	(x10³/μL)	(x10³/μL)	(x10³/μL)	(%)	(%)	(%)	(x10³/μL)	(x10³/μL)	(x10³/μL)
	-7	2	15	-7	2	15	-7	2	15	-7	2	15
2501	32.4	32.4	47.9	0.20	0.28	0.30	2.4	3.6	3.7	0.09	0.11	0.14

Appendix 7 Individual Hematology (Cont'd)

Sex: Female

2<br>100<br>mg/kg	%EOS	%EOS	%EOS	#BASO	#BASO	#BASO	%BASO	%BASO	%BASO	PLT	PLT	PLT
	(%)	(%)	(%)	(x10³/μL)	(x10³/μL)	(x10³/μL)	(%)	(%)	(%)	(x10³/μL)	(x10³/μL)	(x10³/μL)
	-7	2	15	-7	2	15	-7	2	15	-7	2	15
2501	1.1	1.4	1.7	0.02	0.01	0.02	0.2	0.1	0.3	368	370	394

Appendix 7 Individual Hematology (Cont'd)

Sex: Female

2<br>100<br>mg/kg	MPV<br><br>(fL)<br>-7	MPV<br><br>(fL)<br>2	MPV<br><br>(fL)<br>15
2501	10.0	9.9	10.1

Appendix 7 Individual Hematology (Cont'd)

Sex: Female

3<br>300<br>mg/kg	WBC	WBC	WBC	RBC	RBC	RBC	HGB	HGB	HGB	HCT	HCT	HCT
	($x10^3/\mu L$)	($x10^3/\mu L$)	($x10^3/\mu L$)	($x10^6$ /μL)	($x10^6$ /μL)	($x10^6$ /μL)	(g/dL)	(g/dL)	(g/dL)	(%)	(%)	(%)
	-7	2	15	-7	2	15	-7	2	15	-7	2	15
3501	5.87	6.32	7.06	4.99	4.94	4.97	13.0	12.8	12.8	40.2	40.7	39.9

Appendix 7 Individual Hematology (Cont'd)

Sex: Female

3<br>300<br>mg/kg	MCV	MCV	MCV	MCH	MCH	MCH	MCHC	MCHC	MCHC	RDW	RDW	RDW
	(fL)	(fL)	(fL)	(pg)	(pg)	(pg)	(g/dL)	(g/dL)	(g/dL)	(%)	(%)	(%)
	-7	2	15	-7	2	15	-7	2	15	-7	2	15
3501	80.5	82.4	80.3	26.0	25.9	25.7	32.3	31.5	32.1	11.6	11.8	11.7

Appendix 7 Individual Hematology (Cont'd)

Sex: Female

3<br>300<br>mg/kg	#RET	#RET	#RET	#NEUT	#NEUT	#NEUT	%NEUT	%NEUT	%NEUT	#LYMP	#LYMP	#LYMP
	(x10⁹ /L)	(x10⁹ /L)	(x10⁹ /L)	($x10^3/\mu L$)	($x10^3/\mu L$)	($x10^3/\mu L$)	(%)	(%)	(%)	($x10^3/\mu L$)	($x10^3/\mu L$)	($x10^3/\mu L$)
	-7	2	15	-7	2	15	-7	2	15	-7	2	15
3501	50.8	64.8	55.0	3.19	3.70	3.78	54.3	58.5	53.5	2.43	2.26	2.84

Appendix 7 Individual Hematology (Cont'd)

Sex: Female

3<br>300<br>mg/kg	%LYMP	%LYMP	%LYMP	#MONO	#MONO	#MONO	%MONO	%MONO	%MONO	#EOS	#EOS	#EOS
	(%)	(%)	(%)	(x10³/µL)	(x10³/µL)	(x10³/µL)	(%)	(%)	(%)	(x10³/µL)	(x10³/µL)	(x10³/µL)
	-7	2	15	-7	2	15	-7	2	15	-7	2	15
3501	41.4	35.8	40.2	0.17	0.24	0.22	2.9	3.7	3.1	0.03	0.06	0.09

Appendix 7 Individual Hematology (Cont'd)

Sex: Female

3<br>300<br>mg/kg	%EOS	%EOS	%EOS	#BASO	#BASO	#BASO	%BASO	%BASO	%BASO	PLT	PLT	PLT
	(%)	(%)	(%)	(x10³/μL)	(x10³/μL)	(x10³/μL)	(%)	(%)	(%)	(x10³/μL)	(x10³/μL)	(x10³/μL)
	-7	2	15	-7	2	15	-7	2	15	-7	2	15
3501	0.5	0.9	1.3	0.01	0.01	0.02	0.2	0.1	0.2	304	294	283

Appendix 7 Individual Hematology (Cont'd)

Sex: Female

3<br>300<br>mg/kg	MPV	MPV	MPV
	(fL)	(fL)	(fL)
	-7	2	15
3501	10.9	11.0	10.8

Appendix 7 Individual Hematology (Cont'd)

Sex: Female

4<br>1000<br>mg/kg	WBC	WBC	WBC	RBC	RBC	RBC	HGB	HGB	HGB	HCT	HCT	HCT
	($x10^3/\mu L$)	($x10^3/\mu L$)	($x10^3/\mu L$)	($x10^6$ /μL)	($x10^6$ /μL)	($x10^6$ /μL)	(g/dL)	(g/dL)	(g/dL)	(%)	(%)	(%)
	-7	2	15	-7	2	15	-7	2	15	-7	2	15
4501	10.39	6.52	8.05	5.29	5.13	5.81	12.6	12.1	13.5	40.7	40.1	44.3

Appendix 7 Individual Hematology (Cont'd)

Sex: Female

4<br>1000<br>mg/kg	MCV	MCV	MCV	MCH	MCH	MCH	MCHC	MCHC	MCHC	RDW	RDW	RDW
	(fL)	(fL)	(fL)	(pg)	(pg)	(pg)	(g/dL)	(g/dL)	(g/dL)	(%)	(%)	(%)
	-7	2	15	-7	2	15	-7	2	15	-7	2	15
4501	77.0	78.2	76.3	23.8	23.6	23.2	30.9	30.1	30.4	12.5	12.8	12.9

Appendix 7 Individual Hematology (Cont'd)

Sex: Female

4<br>1000<br>mg/kg	#RET	#RET	#RET	#NEUT	#NEUT	#NEUT	%NEUT	%NEUT	%NEUT	#LYMP	#LYMP	#LYMP
	(x10⁹ /L)	(x10⁹ /L)	(x10⁹ /L)	(x10³/µL)	(x10³/µL)	(x10³/µL)	(%)	(%)	(%)	(x10³/µL)	(x10³/µL)	(x10³/µL)
	-7	2	15	-7	2	15	-7	2	15	-7	2	15
4501	47.9	53.6	86.2	7.02	3.91	3.66	67.6	60.1	45.4	2.98	2.18	4.06

Appendix 7 Individual Hematology (Cont'd)

Sex: Female

4<br>1000<br>mg/kg	%LYMP	%LYMP	%LYMP	#MONO	#MONO	#MONO	%MONO	%MONO	%MONO	#EOS	#EOS	#EOS
	(%)	(%)	(%)	($x10^3/\mu L$)	($x10^3/\mu L$)	($x10^3/\mu L$)	(%)	(%)	(%)	($x10^3/\mu L$)	($x10^3/\mu L$)	($x10^3/\mu L$)
	-7	2	15	-7	2	15	-7	2	15	-7	2	15
4501	28.7	33.5	50.4	0.29	0.32	0.18	2.8	4.8	2.2	0.04	0.05	0.08

Appendix 7 Individual Hematology (Cont'd)

Sex: Female

4<br>1000<br>mg/kg	%EOS	%EOS	%EOS	#BASO	#BASO	#BASO	%BASO	%BASO	%BASO	PLT	PLT	PLT
	(%)	(%)	(%)	(x10³/µL)	(x10³/µL)	(x10³/µL)	(%)	(%)	(%)	(x10³/µL)	(x10³/µL)	(x10³/µL)
	-7	2	15	-7	2	15	-7	2	15	-7	2	15
4501	0.4	0.8	1.0	0.01	0.01	0.03	0.1	0.1	0.3	404	399	385

Appendix 7 Individual Hematology (Cont'd)

Sex: Female

4<br>1000<br>mg/kg	MPV<br><br>(fL)<br>-7	MPV<br><br>(fL)<br>2	MPV<br><br>(fL)<br>15
4501	7.6	7.7	7.7

Appendix 8 Individual Coagulation

Appendix 8 Individual Coagulation

Key Page

Measurement Descriptions

Headings Used	Description
PT	Prothrombin Time
APTT	Activated Partial Thromboplastin Time
FIB	Fibrinogen

Appendix 8 Individual Coagulation (Cont'd)

Sex: Male

1<br>0<br>mg/kg	PT	PT	PT	APTT	APTT	APTT	FIB	FIB	FIB
	(Sec)	(Sec)	(Sec)	(Sec)	(Sec)	(Sec)	(g/L)	(g/L)	(g/L)
	-7	2	15	-7	2	15	-7	2	15
1001	11.0	9.2	10.2	19.3	18.8	18.4	3.19	4.36	2.87

Appendix 8 Individual Coagulation (Cont'd)

Sex: Male

2<br>100<br>mg/kg	PT	PT	PT	APTT	APTT	APTT	FIB	FIB	FIB
	(Sec)	(Sec)	(Sec)	(Sec)	(Sec)	(Sec)	(g/L)	(g/L)	(g/L)
	-7	2	15	-7	2	15	-7	2	15
2001	10.3	10.2	10.3	19.4	18.3	18.8	2.93	3.04	2.64

Appendix 8 Individual Coagulation (Cont'd)

Sex: Male

3<br>300<br>mg/kg	PT<br>(Sec)<br>-7	PT<br>(Sec)<br>2	PT<br>(Sec)<br>15	APTT<br>(Sec)<br>-7	APTT<br>(Sec)<br>2	APTT<br>(Sec)<br>15	FIB<br>(g/L)<br>-7	FIB<br>(g/L)<br>2	FIB<br>(g/L)<br>15
3001	10.5	9.9	9.6	19.1	18.0	18.5	3.00	3.66	2.69

Appendix 8 Individual Coagulation (Cont'd)

Sex: Male

4 1000 mg/kg	PT (Sec)	PT (Sec)	PT (Sec)	APTT (Sec)	APTT (Sec)	APTT (Sec)	FIB (g/L)	FIB (g/L)	FIB (g/L)
	-7	2	15	-7	2	15	-7	2	15
4001	11.2	10.5	10.4	16.7	16.5	17.2	2.89	3.37	2.54

Appendix 8 Individual Coagulation (Cont'd)

Sex: Female

1<br>0<br>mg/kg	PT	PT	PT	APTT	APTT	APTT	FIB	FIB	FIB
	(Sec)	(Sec)	(Sec)	(Sec)	(Sec)	(Sec)	(g/L)	(g/L)	(g/L)
	-7	2	15	-7	2	15	-7	2	15
1501	10.5	9.5	10.3	18.6	17.7	19.9	2.37	2.99	2.35

Appendix 8 Individual Coagulation (Cont'd)

Sex: Female

2<br>100<br>mg/kg	PT	PT	PT	APTT	APTT	APTT	FIB	FIB	FIB
	(Sec)	(Sec)	(Sec)	(Sec)	(Sec)	(Sec)	(g/L)	(g/L)	(g/L)
	-7	2	15	-7	2	15	-7	2	15
2501	10.7	10.2	9.5	17.5	16.6	17.6	2.56	3.22	2.86

Appendix 8 Individual Coagulation (Cont'd)

Sex: Female

3<br>300<br>mg/kg	PT<br>(Sec)<br>-7	PT<br>(Sec)<br>2	PT<br>(Sec)<br>15	APTT<br>(Sec)<br>-7	APTT<br>(Sec)<br>2	APTT<br>(Sec)<br>15	FIB<br>(g/L)<br>-7	FIB<br>(g/L)<br>2	FIB<br>(g/L)<br>15
3501	10.1	9.2	9.2	17.5	18.2	19.3	3.29	3.76	3.18

Appendix 8 Individual Coagulation (Cont'd)

Sex: Female

4<br>1000<br>mg/kg	PT<br>(Sec)<br>-7	PT<br>(Sec)<br>2	PT<br>(Sec)<br>15	APTT<br>(Sec)<br>-7	APTT<br>(Sec)<br>2	APTT<br>(Sec)<br>15	FIB<br>(g/L)<br>-7	FIB<br>(g/L)<br>2	FIB<br>(g/L)<br>15
4501	10.5	9.9	9.8	19.5	18.3	19.3	2.69	3.26	2.37

Appendix 9 Individual Chemistry

Appendix 9 Individual Chemistry

Key Page

Measurement Descriptions

Headings Used	Description
ALT	Alanine Aminotransferase
AST	Aspartate Aminotransferase
TP	Total Protein
ALB	Albumin
TBIL	Total Bilirubin
ALP	Alkaline Phosphatase
GGT	Gamma-Glutamyltransferase
sGLU	Glucose
UREA	Urea
CRE	Creatinine
Ca	Calcium
P	Inorganic Phosphorus
TCHO	Total Cholesterol
TG	Triglyceride
K	Potassium
Na	Sodium
Cl	Chloride
GLB	Globulin
A/G	A/G Ratio
CK	Creatine Kinase

SC = Sample Comment

Appendix 9 Individual Chemistry (Cont'd)

Sex: Male

1<br>0<br>mg/kg	ALT<br>(U/L)<br>-7	ALT<br>(U/L)<br>2	ALT<br>(U/L)<br>15	AST<br>(U/L)<br>-7	AST<br>(U/L)<br>2	AST<br>(U/L)<br>15	TP<br>(g/L)<br>-7	TP<br>(g/L)<br>2	TP<br>(g/L)<br>15	ALB<br>(g/L)<br>-7	ALB<br>(g/L)<br>2	ALB<br>(g/L)<br>15
1001	30	229	42	38	926	44	78.2	70.7	74.4	49.5	44.6	47.2

Appendix 9 Individual Chemistry (Cont'd)

Sex: Male

1<br>0<br>mg/kg	TBIL	TBIL	TBIL	ALP	ALP	ALP	GGT	GGT	GGT	sGLU	sGLU	sGLU
	(μmol/L)	(μmol/L)	(μmol/L)	(U/L)	(U/L)	(U/L)	(U/L)	(U/L)	(U/L)	(mmol/L)	(mmol/L)	(mmol/L)
	-7	2	15	-7	2	15	-7	2	15	-7	2	15
1001	6.72	15.52	10.69	451	471	399	96	86	84	3.24	3.58	2.89

Appendix 9 Individual Chemistry (Cont'd)

Sex: Male

10 mg/kg	UREA	UREA	UREA	CRE	CRE	CRE	Ca	Ca	Ca	P	P	P
	(mmol/L)	(mmol/L)	(mmol/L)	(μmol/L)	(μmol/L)	(μmol/L)	(mmol/L)	(mmol/L)	(mmol/L)	(mmol/L)	(mmol/L)	(mmol/L)
	-7	2	15	-7	2	15	-7	2	15	-7	2	15
1001	6.25	4.58	7.94	55	57	55	2.68	2.53	2.59	2.66	1.65	2.43

Appendix 9 Individual Chemistry (Cont'd)

Sex: Male

1<br>0<br>mg/kg	TCHO	TCHO	TCHO	TG	TG	TG	K	K	K	Na	Na	Na
	(mmol/L)	(mmol/L)	(mmol/L)	(mmol/L)	(mmol/L)	(mmol/L)	(mmol/L)	(mmol/L)	(mmol/L)	(mmol/L)	(mmol/L)	(mmol/L)
	-7	2	15	-7	2	15	-7	2	15	-7	2	15
1001	3.48	2.82	3.30	0.28	0.34	0.40	6.0	4.8	5.5	157	148	150

Appendix 9 Individual Chemistry (Cont'd)

Sex: Male

1 0 mg/kg	Cl (mmol/L) -7	Cl (mmol/L) 2	Cl (mmol/L) 15	GLB (g/L) -7	GLB (g/L) 2	GLB (g/L) 15	A/G -7	A/G 2	A/G 15	CK (U/L) -7	CK (U/L) 2	CK (U/L) 15
1001	109	107	107	28.7	26.1	27.2	1.72	1.71	1.74	152	74800	163

Appendix 9 Individual Chemistry (Cont'd)

Sex: Male

2<br>100<br>mg/kg	ALT	ALT	ALT	AST	AST	AST	TP	TP	TP	ALB	ALB	ALB
	(U/L)	(U/L)	(U/L)	(U/L)	(U/L)	(U/L)	(g/L)	(g/L)	(g/L)	(g/L)	(g/L)	(g/L)
	-7	2	15	-7	2	15	-7	2	15	-7	2	15
2001	39	54	50	40	80	41	73.3	76.2	72.7	43.7	44.3	44.6

Appendix 9 Individual Chemistry (Cont'd)

Sex: Male

2<br>100<br>mg/kg	TBIL	TBIL	TBIL	ALP	ALP	ALP	GGT	GGT	GGT	sGLU	sGLU	sGLU
	(μmol/L)	(μmol/L)	(μmol/L)	(U/L)	(U/L)	(U/L)	(U/L)	(U/L)	(U/L)	(mmol/L)	(mmol/L)	(mmol/L)
	-7	2	15	-7	2	15	-7	2	15	-7	2	15
2001	3.90	4.94	6.29	471	479	394	62	65	63	3.91	3.51	2.82

Appendix 9 Individual Chemistry (Cont'd)

Sex: Male

2<br>100<br>mg/kg	UREA	UREA	UREA	CRE	CRE	CRE	Ca	Ca	Ca	P	P	P
	(mmol/L)	(mmol/L)	(mmol/L)	(μmol/L)	(μmol/L)	(μmol/L)	(mmol/L)	(mmol/L)	(mmol/L)	(mmol/L)	(mmol/L)	(mmol/L)
	-7	2	15	-7	2	15	-7	2	15	-7	2	15
2001	4.66	4.10	5.32	57	56	55	2.63	2.60	2.51	1.95	1.73	1.89

Appendix 9 Individual Chemistry (Cont'd)

Sex: Male

2<br>100<br>mg/kg	TCHO	TCHO	TCHO	TG	TG	TG	K	K	K	Na	Na	Na
	(mmol/L)	(mmol/L)	(mmol/L)	(mmol/L)	(mmol/L)	(mmol/L)	(mmol/L)	(mmol/L)	(mmol/L)	(mmol/L)	(mmol/L)	(mmol/L)
	-7	2	15	-7	2	15	-7	2	15	-7	2	15
2001	2.65	2.50	2.54	0.51	0.52	0.47	5.3	4.9	4.5	148	147	145

Appendix 9 Individual Chemistry (Cont'd)

Sex: Male

2<br>100<br>mg/kg	Cl	Cl	Cl	GLB	GLB	GLB	A/G	A/G	A/G	CK	CK	CK
	(mmol/L)	(mmol/L)	(mmol/L)	(g/L)	(g/L)	(g/L)				(U/L)	(U/L)	(U/L)
	-7	2	15	-7	2	15	-7	2	15	-7	2	15
2001	105	105	103	29.6	31.9	28.1	1.48	1.39	1.59	152	876	140

Appendix 9 Individual Chemistry (Cont'd)

Sex: Male

3<br>300<br>mg/kg	ALT	ALT	ALT	AST	AST	AST	TP	TP	TP	ALB	ALB	ALB
	(U/L)	(U/L)	(U/L)	(U/L)	(U/L)	(U/L)	(g/L)	(g/L)	(g/L)	(g/L)	(g/L)	(g/L)
	-7	2	15	-7	2	15	-7	2	15	-7	2	15
3001	65	77	70	54	208	56	79.3	82.3	79.4	47.2	45.4	46.4

Appendix 9 Individual Chemistry (Cont'd)

Sex: Male

3<br>300<br>mg/kg	TBIL	TBIL	TBIL	ALP	ALP	ALP	GGT	GGT	GGT	sGLU	sGLU	sGLU
	(µmol/L)	(µmol/L)	(µmol/L)	(U/L)	(U/L)	(U/L)	(U/L)	(U/L)	(U/L)	(mmol/L)	(mmol/L)	(mmol/L)
	-7	2	15	-7	2	15	-7	2	15	-7	2	15
3001	4.11	6.74	5.37	426	393	354	65	57	60	3.34	2.85	3.44

Appendix 9 Individual Chemistry (Cont'd)

Sex: Male

3<br>300<br>mg/kg	UREA	UREA	UREA	CRE	CRE	CRE	Ca	Ca	Ca	P	P	P
	(mmol/L)	(mmol/L)	(mmol/L)	(μmol/L)	(μmol/L)	(μmol/L)	(mmol/L)	(mmol/L)	(mmol/L)	(mmol/L)	(mmol/L)	(mmol/L)
	-7	2	15	-7	2	15	-7	2	15	-7	2	15
3001	4.54	3.84	5.71	53	49	50	2.73	2.64	2.73	1.56	1.23	1.73

Appendix 9 Individual Chemistry (Cont'd)

Sex: Male

3<br>300<br>mg/kg	TCHO	TCHO	TCHO	TG	TG	TG	K	K	K	Na	Na	Na
	(mmol/L)	(mmol/L)	(mmol/L)	(mmol/L)	(mmol/L)	(mmol/L)	(mmol/L)	(mmol/L)	(mmol/L)	(mmol/L)	(mmol/L)	(mmol/L)
	-7	2	15	-7	2	15	-7	2	15	-7	2	15
3001	4.63	3.30	4.16	0.57	0.34	0.48	5.7	5.0	5.2	152	148	145

Appendix 9 Individual Chemistry (Cont'd)

Sex: Male

3<br>300<br>mg/kg	Cl	Cl	Cl	GLB	GLB	GLB	A/G	A/G	A/G	CK	CK	CK
	(mmol/L)	(mmol/L)	(mmol/L)	(g/L)	(g/L)	(g/L)				(U/L)	(U/L)	(U/L)
	-7	2	15	-7	2	15	-7	2	15	-7	2	15
3001	106	105	101	32.1	36.9	33.0	1.47	1.23	1.41	300	12300	242

Appendix 9 Individual Chemistry (Cont'd)

Sex: Male

4<br>1000<br>mg/kg	ALT	ALT	ALT	AST	AST	AST	TP	TP	TP	ALB	ALB	ALB
	(U/L)	(U/L)	(U/L)	(U/L)	(U/L)	(U/L)	(g/L)	(g/L)	(g/L)	(g/L)	(g/L)	(g/L)
	-7	2	15	-7	2	15	-7	2	15	-7	2	15
4001	45 ![1]	97	38	69 ![1]	208	42	74.7 ![1]	88.5	80.6	44.9 ![1]	41.5	46.4

[1][SC:Slight Hemolysis]

Appendix 9 Individual Chemistry (Cont'd)

Sex: Male

4 1000 mg/kg	TBIL	TBIL	TBIL	ALP	ALP	ALP	GGT	GGT	GGT	sGLU	sGLU	sGLU
	(µmol/L)	(µmol/L)	(µmol/L)	(U/L)	(U/L)	(U/L)	(U/L)	(U/L)	(U/L)	(mmol/L)	(mmol/L)	(mmol/L)
	-7	2	15	-7	2	15	-7	2	15	-7	2	15
4001	3.58 ![1]	3.70	4.46	561 ![1]	454	419	79 ![1]	67	74	4.66 ![1]	3.64	3.71

[1][SC:Slight Hemolysis]

Appendix 9 Individual Chemistry (Cont'd)

Sex: Male

4<br>1000<br>mg/kg	UREA	UREA	UREA	CRE	CRE	CRE	Ca	Ca	Ca	P	P	P
	(mmol/L)	(mmol/L)	(mmol/L)	(µmol/L)	(µmol/L)	(µmol/L)	(mmol/L)	(mmol/L)	(mmol/L)	(mmol/L)	(mmol/L)	(mmol/L)
	-7	2	15	-7	2	15	-7	2	15	-7	2	15
4001	4.52 ![1]	3.82	6.43	58 ![1]	59	67	2.53 ![1]	2.53	2.63	1.90 ![1]	1.80	1.96

[1][SC:Slight Hemolysis]

Appendix 9 Individual Chemistry (Cont'd)

Sex: Male

4<br>1000<br>mg/kg	TCHO<br>(mmol/L)<br>-7	TCHO<br>(mmol/L)<br>2	TCHO<br>(mmol/L)<br>15	TG<br>(mmol/L)<br>-7	TG<br>(mmol/L)<br>2	TG<br>(mmol/L)<br>15	K<br>(mmol/L)<br>-7	K<br>(mmol/L)<br>2	K<br>(mmol/L)<br>15	Na<br>(mmol/L)<br>-7	Na<br>(mmol/L)<br>2	Na<br>(mmol/L)<br>15
4001	3.29 ![1]	2.52	3.23	0.37 ![1]	0.22	0.39	5.2 ![1]	5.6	4.7	144 ![1]	145	147

[1][SC:Slight Hemolysis]

Appendix 9 Individual Chemistry (Cont'd)

Sex: Male

4<br>1000<br>mg/kg	Cl<br>(mmol/L)<br>-7	Cl<br>(mmol/L)<br>2	Cl<br>(mmol/L)<br>15	GLB<br>(g/L)<br>-7	GLB<br>(g/L)<br>2	GLB<br>(g/L)<br>15	A/G<br><br>-7	A/G<br><br>2	A/G<br><br>15	CK<br>(U/L)<br>-7	CK<br>(U/L)<br>2	CK<br>(U/L)<br>15
4001	103 ![1]	106	103	29.8	47.0	34.2	1.51	0.88	1.36	2220 ![2]	2390	264

[1][SC:Slight Hemolysis]
[2][SC:Slight Hemolysis]

Appendix 9 Individual Chemistry (Cont'd)

Sex: Female

10 mg/kg	ALT	ALT	ALT	AST	AST	AST	TP	TP	TP	ALB	ALB	ALB
	(U/L)	(U/L)	(U/L)	(U/L)	(U/L)	(U/L)	(g/L)	(g/L)	(g/L)	(g/L)	(g/L)	(g/L)
	-7	2	15	-7	2	15	-7	2	15	-7	2	15
1501	33	83 ![1]	39	52	255 ![2]	40	71.1	66.8 ![3]	71.6	39.1	36.0 ![3]	39.2

[1][SC:Slight Hemolysis]
[2][SC:Slight Hemolysis]
[3][SC:Slight Hemolysis]

Appendix 9 Individual Chemistry (Cont'd)

Sex: Female

10 mg/kg	TBIL (µmol/L) -7	TBIL (µmol/L) 2	TBIL (µmol/L) 15	ALP (U/L) -7	ALP (U/L) 2	ALP (U/L) 15	GGT (U/L) -7	GGT (U/L) 2	GGT (U/L) 15	sGLU (mmol/L) -7	sGLU (mmol/L) 2	sGLU (mmol/L) 15
1501	7.69	8.10 ![1]	6.30	461	340 ![1]	344	101	85 ![1]	93	3.17	3.73 ![1]	3.98

[1][SC:Slight Hemolysis]

Appendix 9 Individual Chemistry (Cont'd)

Sex: Female

1<br>0<br>mg/kg	UREA	UREA	UREA	CRE	CRE	CRE	Ca	Ca	Ca	P	P	P
	(mmol/L)	(mmol/L)	(mmol/L)	(µmol/L)	(µmol/L)	(µmol/L)	(mmol/L)	(mmol/L)	(mmol/L)	(mmol/L)	(mmol/L)	(mmol/L)
	-7	2	15	-7	2	15	-7	2	15	-7	2	15
1501	7.23	3.32 ![1]	5.21	56	45 ![2]	48	2.51	2.37 ![2]	2.44	1.67	1.46 ![2]	1.57

[1][SC:Slight Hemolysis]
[2][SC:Slight Hemolysis]

Appendix 9 Individual Chemistry (Cont'd)

Sex: Female

10 mg/kg	TCHO (mmol/L) -7	TCHO (mmol/L) 2	TCHO (mmol/L) 15	TG (mmol/L) -7	TG (mmol/L) 2	TG (mmol/L) 15	K (mmol/L) -7	K (mmol/L) 2	K (mmol/L) 15	Na (mmol/L) -7	Na (mmol/L) 2	Na (mmol/L) 15
1501	2.92	2.27 ![1]	2.63	0.47	0.39 ![1]	0.54	5.7	5.7 ![1]	5.4	149	145 ![1]	146

[1][SC:Slight Hemolysis]

Appendix 9 Individual Chemistry (Cont'd)

Sex: Female

1 0 mg/kg	Cl (mmol/L)	Cl (mmol/L)	Cl (mmol/L)	GLB (g/L)	GLB (g/L)	GLB (g/L)	A/G	A/G	A/G	CK (U/L)	CK (U/L)	CK (U/L)
	-7	2	15	-7	2	15	-7	2	15	-7	2	15
1501	107	107 ![1]	105	32.0	30.8	32.4	1.22	1.17	1.21	550	1280 ![2]	178

[1][SC:Slight Hemolysis]
[2][SC:Slight Hemolysis]

Appendix 9 Individual Chemistry (Cont'd)

Sex: Female

2<br>100<br>mg/kg	ALT	ALT	ALT	AST	AST	AST	TP	TP	TP	ALB	ALB	ALB
	(U/L)	(U/L)	(U/L)	(U/L)	(U/L)	(U/L)	(g/L)	(g/L)	(g/L)	(g/L)	(g/L)	(g/L)
	-7	2	15	-7	2	15	-7	2	15	-7	2	15
2501	38	59	35	29	73	30	74.5	75.0	76.9	44.1	41.7	44.2

Appendix 9 Individual Chemistry (Cont'd)

Sex: Female

2<br>100<br>mg/kg	TBIL	TBIL	TBIL	ALP	ALP	ALP	GGT	GGT	GGT	sGLU	sGLU	sGLU
	(μmol/L)	(μmol/L)	(μmol/L)	(U/L)	(U/L)	(U/L)	(U/L)	(U/L)	(U/L)	(mmol/L)	(mmol/L)	(mmol/L)
	-7	2	15	-7	2	15	-7	2	15	-7	2	15
2501	4.52	4.76	3.33	223	212	194	50	47	51	2.58	3.29	3.13

Appendix 9 Individual Chemistry (Cont'd)

Sex: Female

2<br>100<br>mg/kg	UREA<br>(mmol/L)<br>-7	UREA<br>(mmol/L)<br>2	UREA<br>(mmol/L)<br>15	CRE<br>(µmol/L)<br>-7	CRE<br>(µmol/L)<br>2	CRE<br>(µmol/L)<br>15	Ca<br>(mmol/L)<br>-7	Ca<br>(mmol/L)<br>2	Ca<br>(mmol/L)<br>15	P<br>(mmol/L)<br>-7	P<br>(mmol/L)<br>2	P<br>(mmol/L)<br>15
2501	7.42	5.37	6.21	71	69	68	2.54	2.52	2.63	1.93	1.38	1.72

Appendix 9 Individual Chemistry (Cont'd)

Sex: Female

2<br>100<br>mg/kg	TCHO	TCHO	TCHO	TG	TG	TG	K	K	K	Na	Na	Na
	(mmol/L)	(mmol/L)	(mmol/L)	(mmol/L)	(mmol/L)	(mmol/L)	(mmol/L)	(mmol/L)	(mmol/L)	(mmol/L)	(mmol/L)	(mmol/L)
	-7	2	15	-7	2	15	-7	2	15	-7	2	15
2501	4.45	3.94	4.39	0.38	0.32	0.51	5.1	5.5	4.8	147	146	149

Appendix 9 Individual Chemistry (Cont'd)

Sex: Female

2<br>100<br>mg/kg	Cl	Cl	Cl	GLB	GLB	GLB	A/G	A/G	A/G	CK	CK	CK
	(mmol/L)	(mmol/L)	(mmol/L)	(g/L)	(g/L)	(g/L)				(U/L)	(U/L)	(U/L)
	-7	2	15	-7	2	15	-7	2	15	-7	2	15
2501	108	107	105	30.4	33.3	32.7	1.45	1.25	1.35	182	1390	192

Appendix 9 Individual Chemistry (Cont'd)

Sex: Female

3<br>300<br>mg/kg	ALT	ALT	ALT	AST	AST	AST	TP	TP	TP	ALB	ALB	ALB
	(U/L)	(U/L)	(U/L)	(U/L)	(U/L)	(U/L)	(g/L)	(g/L)	(g/L)	(g/L)	(g/L)	(g/L)
	-7	2	15	-7	2	15	-7	2	15	-7	2	15
3501	80	88	52	47	131	36	74.2	79.0	74.6	42.7	42.9	41.2

Appendix 9 Individual Chemistry (Cont'd)

Sex: Female

3<br>300<br>mg/kg	TBIL	TBIL	TBIL	ALP	ALP	ALP	GGT	GGT	GGT	sGLU	sGLU	sGLU
	(μmol/L)	(μmol/L)	(μmol/L)	(U/L)	(U/L)	(U/L)	(U/L)	(U/L)	(U/L)	(mmol/L)	(mmol/L)	(mmol/L)
	-7	2	15	-7	2	15	-7	2	15	-7	2	15
3501	3.86	6.25	3.63	264	289	258	50	50	50	3.10	1.98	3.34

Appendix 9 Individual Chemistry (Cont'd)

Sex: Female

3<br>300<br>mg/kg	UREA	UREA	UREA	CRE	CRE	CRE	Ca	Ca	Ca	P	P	P
	(mmol/L)	(mmol/L)	(mmol/L)	(μmol/L)	(μmol/L)	(μmol/L)	(mmol/L)	(mmol/L)	(mmol/L)	(mmol/L)	(mmol/L)	(mmol/L)
	-7	2	15	-7	2	15	-7	2	15	-7	2	15
3501	5.65	5.30	6.78	64	62	63	2.61	2.56	2.52	1.37	1.18	1.06

Appendix 9 Individual Chemistry (Cont'd)

Sex: Female

3<br>300<br>mg/kg	TCHO	TCHO	TCHO	TG	TG	TG	K	K	K	Na	Na	Na
	(mmol/L)	(mmol/L)	(mmol/L)	(mmol/L)	(mmol/L)	(mmol/L)	(mmol/L)	(mmol/L)	(mmol/L)	(mmol/L)	(mmol/L)	(mmol/L)
	-7	2	15	-7	2	15	-7	2	15	-7	2	15
3501	4.53	4.03	4.61	0.30	0.36	0.50	5.0	4.6	4.1	149	148	145

Appendix 9 Individual Chemistry (Cont'd)

Sex: Female

3<br>300<br>mg/kg	Cl	Cl	Cl	GLB	GLB	GLB	A/G	A/G	A/G	CK	CK	CK
	(mmol/L)	(mmol/L)	(mmol/L)	(g/L)	(g/L)	(g/L)				(U/L)	(U/L)	(U/L)
	-7	2	15	-7	2	15	-7	2	15	-7	2	15
3501	107	106	105	31.5	36.1	33.4	1.36	1.19	1.23	214	1250	249

Appendix 9 Individual Chemistry (Cont'd)

Sex: Female

4<br>1000<br>mg/kg	ALT	ALT	ALT	AST	AST	AST	TP	TP	TP	ALB	ALB	ALB
	(U/L)	(U/L)	(U/L)	(U/L)	(U/L)	(U/L)	(g/L)	(g/L)	(g/L)	(g/L)	(g/L)	(g/L)
	-7	2	15	-7	2	15	-7	2	15	-7	2	15
4501	35	54	64	37	150	36	70.0	86.4	77.1	39.9	36.6	40.2

Appendix 9 Individual Chemistry (Cont'd)

Sex: Female

4<br>1000<br>mg/kg	TBIL	TBIL	TBIL	ALP	ALP	ALP	GGT	GGT	GGT	sGLU	sGLU	sGLU
	(μmol/L)	(μmol/L)	(μmol/L)	(U/L)	(U/L)	(U/L)	(U/L)	(U/L)	(U/L)	(mmol/L)	(mmol/L)	(mmol/L)
	-7	2	15	-7	2	15	-7	2	15	-7	2	15
4501	5.55	6.35	5.25	239	187	208	54	45	53	2.96	3.26	3.44

Appendix 9 Individual Chemistry (Cont'd)

Sex: Female

4<br>1000<br>mg/kg	UREA	UREA	UREA	CRE	CRE	CRE	Ca	Ca	Ca	P	P	P
	(mmol/L)	(mmol/L)	(mmol/L)	(µmol/L)	(µmol/L)	(µmol/L)	(mmol/L)	(mmol/L)	(mmol/L)	(mmol/L)	(mmol/L)	(mmol/L)
	-7	2	15	-7	2	15	-7	2	15	-7	2	15
4501	4.81	3.25	5.15	63	57	61	2.55	2.43	2.54	1.38	1.05	1.24

Appendix 9 Individual Chemistry (Cont'd)

Sex: Female

4<br>1000<br>mg/kg	TCHO	TCHO	TCHO	TG	TG	TG	K	K	K	Na	Na	Na
	(mmol/L)	(mmol/L)	(mmol/L)	(mmol/L)	(mmol/L)	(mmol/L)	(mmol/L)	(mmol/L)	(mmol/L)	(mmol/L)	(mmol/L)	(mmol/L)
	-7	2	15	-7	2	15	-7	2	15	-7	2	15
4501	3.63	2.46	3.92	0.53	0.37	0.81	4.8	4.8	4.2	145	143	142

Appendix 9 Individual Chemistry (Cont'd)

Sex: Female

4<br>1000<br>mg/kg	Cl	Cl	Cl	GLB	GLB	GLB	A/G	A/G	A/G	CK	CK	CK
	(mmol/L)	(mmol/L)	(mmol/L)	(g/L)	(g/L)	(g/L)				(U/L)	(U/L)	(U/L)
	-7	2	15	-7	2	15	-7	2	15	-7	2	15
4501	106	106	102	30.1	49.8	36.9	1.33	0.73	1.09	144	799	182
