# Supplementary material for: Preclinical Characterization of GLS-010 (Zimberelimab), a Novel Fully Human Anti-PD-1 Therapeutic Monoclonal Antibody for Cancer
Source: Front Oncol. 2021 Sep 15;11:736955. doi: 10.3389/fonc.2021.736955 (PMC8479189; doi:10.3389/fonc.2021.736955)
Supplement: Supplementary file 2 [file DataSheet_2.pdf]

Table 5 Summary of Hematology

Table 5 Summary of Hematology

Key Page

Measurement Descriptions

Headings Used	Description
WBC	Leukocyte count
RBC	Erythrocyte count
HGB	Hemoglobin
HCT	Hematocrit
MCV	Mean Corpuscular Volume
MCH	Mean Corpuscular Hemoglobin
MCHC	Mean Corpuscular Hemoglobin Conc.
RDW	RBC Distribution Width
#RET	Reticulocytes, absolute
#NEUT	Neutrophils, absolute
%NEUT	Neutrophils, percent
#LYMP	Lymphocytes, absolute
%LYMP	Lymphocytes, percent
#MONO	Monocytes, absolute
%MONO	Monocytes, percent
#EOS	Eosinophils, absolute
%EOS	Eosinophils, percent
#BASO	Basophils, absolute
%BASO	Basophils, percent
PLT	Platelet Count
MPV	Mean Platelet Volume

Table 5 Summary of Hematology-Pretest and Dosing Phase

Day(s) Relative to Start Date

Sex: Male		WBC (x10^3/µL) [a] -11	WBC (x10^3/µL) [a1] -6	WBC (x10^3/µL) [a1] 53	WBC (x10^3/µL) [a1] 87	WBC (x10^3/µL) [a1] 123	WBC (x10^3/µL) [a1] 179	RBC (x10^6/µL) [a1] -11	RBC (x10^6/µL) [a1] -6	RBC (x10^6/µL) [a1] 53	RBC (x10^6/µL) [a1] 87	RBC (x10^6/µL) [a1] 123	RBC (x10^6/µL) [a1] 179
1	Mean	10.80	11.39	10.28	9.64	7.88	9.89	5.71	5.50	5.85	5.75	5.80	5.65
0	SD	4.25	2.95	2.73	4.09	0.87	4.00	0.30	0.27	0.23	0.24	0.34	0.29
mg/kg/dose	N	6	6	6	6	6	6	6	6	6	6	6	6
2	Mean	9.55	10.87	9.58	11.18	8.64	8.35	5.63	5.49	5.98	5.75	5.85	5.63
5	SD	3.10	4.29	2.77	4.12	3.67	2.31	0.62	0.57	0.36	0.42	0.35	0.45
mg/kg/dose	N	6	6	6	6	6	6	6	6	6	6	6	6
	%Diff	-11.52	-4.57	-6.84	15.99	9.53	-15.55	-1.40	-0.21	2.28	-0.14	0.83	-0.47
3	Mean	8.70	9.89	9.32	7.92	9.29	7.37	5.75	5.43	5.95	5.59	5.69	5.79
25	SD	1.32	1.90	1.15	1.49	1.45	1.76	0.47	0.41	0.40	0.70	0.44	0.30
mg/kg/dose	N	6	6	6	6	6	6	6	6	6	6	6	6
	%Diff	-19.45	-13.16	-9.31	-17.87	17.89	-25.51	0.64	-1.36	1.74	-2.87	-1.81	2.45
4	Mean	8.51	11.24	11.71	10.16	10.07	7.59	5.79	5.70	5.99	5.57	5.85	5.64
100	SD	1.99	3.30	5.44	5.82	4.73	1.97	0.11	0.26	0.33	0.75	0.79	0.64
mg/kg/dose	N	6	6	6	6	6	6	6	6	6	6	6	6
	%Diff	-21.21	-1.27	13.89	5.38	27.74	-23.31	1.34	3.58	2.40	-3.16	0.80	-0.18

[a] - Anova & Dunnett(Log)
[a1] - Anova & Dunnett

Table 5 Summary of Hematology-Pretest and Dosing Phase (Cont'd)

Day(s) Relative to Start Date

Sex: Male		HGB (g/dL) [a] -11	HGB (g/dL) [a1] -6	HGB (g/dL) [a] 53	HGB (g/dL) [a] 87	HGB (g/dL) [a] 123	HGB (g/dL) [a] 179	HCT (%) [a] -11	HCT (%) [a] -6	HCT (%) [a] 53	HCT (%) [a] 87	HCT (%) [a] 123	HCT (%) [a] 179
1	Mean	13.2	12.6	13.5	13.2	13.6	13.3	43.3	42.4	44.6	43.5	43.3	43.0
0	SD	0.5	0.2	0.7	0.5	0.4	0.6	2.1	1.5	3.9	1.5	1.7	2.3
mg/kg/dose	N	6	6	6	6	6	6	6	6	6	6	6	6
2	Mean	12.6	12.3	13.2	12.7	13.2	12.7	41.5	41.1	42.9	41.5	42.2	41.2
5	SD	1.4	1.2	0.6	0.8	0.9	0.9	4.2	3.3	1.6	2.7	2.4	3.0
mg/kg/dose	N	6	6	6	6	6	6	6	6	6	6	6	6
	%Diff	-4.2	-2.5	-2.1	-4.0	-2.6	-4.5	-4.1	-3.0	-3.7	-4.6	-2.6	-4.3
3	Mean	13.7	12.9	14.0	13.1	13.7	13.8	43.9	42.1	44.8	42.2	43.3	44.2
25	SD	0.5	0.5	0.3	1.4	0.6	0.5	1.1	1.8	2.1	4.9	2.1	1.4
mg/kg/dose	N	6	6	6	6	6	6	6	6	6	6	6	6
	%Diff	3.8	1.8	4.2	-0.4	1.2	4.3	1.5	-0.7	0.4	-2.9	0.1	2.7
4	Mean	13.3	13.1	13.6	12.6	13.5	13.0	42.8	42.9	43.8	41.5	43.8	42.6
100	SD	0.5	0.6	0.4	1.5	1.7	1.3	2.1	2.9	1.3	2.7	3.8	2.4
mg/kg/dose	N	6	6	6	6	6	6	6	6	6	6	6	6
	%Diff	1.0	3.4	0.9	-4.6	-0.1	-2.1	-1.3	1.3	-1.8	-4.4	1.1	-0.9

[a] - Anova & Dunnett
[a1] - Kruskal-Wallis & Dunnett on Ranks

Table 5 Summary of Hematology-Pretest and Dosing Phase (Cont'd)

Day(s) Relative to Start Date

Sex: Male		MCV (fL) [a] -11	MCV (fL) [a] -6	MCV (fL) [a] 53	MCV (fL) [a] 87	MCV (fL) [a] 123	MCV (fL) [a] 179	MCH (pg) [a] -11	MCH (pg) [a] -6	MCH (pg) [a] 53	MCH (pg) [a] 87	MCH (pg) [a] 123	MCH (pg) [a] 179
1	Mean	76.0	77.1	76.2	75.6	74.9	76.1	23.0	23.0	23.0	23.0	23.4	23.5
0	SD	3.9	3.6	4.5	3.7	3.7	3.4	0.7	0.9	0.6	0.8	0.7	0.9
mg/kg/dose	N	6	6	6	6	6	6	6	6	6	6	6	6
2	Mean	74.0	75.1	72.0	72.3	72.3	73.3	22.4	22.5	22.1	22.1	22.6	22.6
5	SD	5.2	4.3	4.3	4.7	4.6	5.0	1.6	1.7	1.5	1.6	1.5	1.7
mg/kg/dose	N	6	6	6	6	6	6	6	6	6	6	6	6
	%Diff	-2.7	-2.6	-5.6	-4.3	-3.4	-3.7	-2.6	-2.2	-4.0	-3.8	-3.6	-3.9
3	Mean	76.8	77.7	75.4	75.6	76.3	76.3	23.9	23.8	23.7	23.6	24.2	23.9
25	SD	4.9	4.5	3.1	3.1	2.9	2.7	1.3	1.3	1.1	0.9	1.0	0.7
mg/kg/dose	N	6	6	6	6	6	6	6	6	6	6	6	6
	%Diff	1.1	0.8	-1.1	0.1	1.9	0.3	3.6	3.4	2.7	2.8	3.1	1.6
4	Mean	73.9	75.3	73.3	75.3	75.4	76.0	23.0	23.0	22.7	22.6	23.2	23.1
100	SD	3.0	3.4	3.3	7.3	5.8	5.4	0.8	0.8	0.8	0.9	0.9	0.8
mg/kg/dose	N	6	6	6	6	6	6	6	6	6	6	6	6
	%Diff	-2.8	-2.4	-3.9	-0.4	0.7	-0.2	-0.3	-0.1	-1.3	-1.5	-0.9	-1.8

[a] - Anova & Dunnett

Table 5 Summary of Hematology-Pretest and Dosing Phase (Cont'd)

Day(s) Relative to Start Date

Sex: Male		MCHC (g/dL) [a] -11	MCHC (g/dL) [a] -6	MCHC (g/dL) [a] 53	MCHC (g/dL) [a] 87	MCHC (g/dL) [a] 123	MCHC (g/dL) [a] 179	RDW (%) [a] -11	RDW (%) [a] -6	RDW (%) [a] 53	RDW (%) [a] 87	RDW (%) [a] 123	RDW (%) [a] 179
1	Mean	30.4	29.8	30.3	30.4	31.3	30.9	13.5	13.5	13.3	13.5	13.0	13.6
0	SD	0.7	0.7	1.1	0.9	0.9	0.6	0.6	0.5	0.3	0.5	0.7	0.5
mg/kg/dose	N	6	6	6	6	6	6	6	6	6	6	6	6
2	Mean	30.3	29.9	30.7	30.5	31.3	30.8	14.1	13.8	13.3	13.7	12.8	14.0
5	SD	0.5	0.9	0.7	0.6	0.8	1.0	1.4	1.1	0.7	0.8	0.5	0.6
mg/kg/dose	N	6	6	6	6	6	6	6	6	6	6	6	6
	%Diff	-0.2	0.2	1.4	0.4	-0.2	-0.3	4.2	2.2	0.0	1.4	-1.5	2.4
3	Mean	31.1	30.6	31.4	31.2	31.7	31.3	13.8	13.5	12.8	13.7	12.8	13.6
25	SD	0.5	0.6	0.9	0.6	0.5	0.2	0.7	0.5	0.5	0.9	0.7	0.4
mg/kg/dose	N	6	6	6	6	6	6	6	6	6	6	6	6
	%Diff	2.4	2.6	3.7	2.7	1.2	1.3	1.9	0.4	-3.1	1.6	-1.3	-0.5
4	Mean	31.1	30.5	31.1	30.2	30.9	30.4	13.7	13.6	13.1	13.5	12.8	13.6
100	SD	0.7	0.9	1.0	2.2	1.7	1.8	0.4	0.4	0.4	0.4	0.4	0.5
mg/kg/dose	N	6	6	6	6	6	6	6	6	6	6	6	6
	%Diff	2.3	2.3	2.6	-0.6	-1.4	-1.6	1.1	1.2	-1.0	0.2	-1.5	-0.2

[a] - Anova & Dunnett

Table 5 Summary of Hematology-Pretest and Dosing Phase (Cont'd)

Day(s) Relative to Start Date

Sex: Male		#RET (x10^9/L) [a] -11	#RET (x10^9/L) [a1] -6	#RET (x10^9/L) [a1] 53	#RET (x10^9/L) [a1] 87	#RET (x10^9/L) [a] 123	#RET (x10^9/L) [a] 179	#NEUT (x10^3/µL) [a1] -11	#NEUT (x10^3/µL) [a1] -6	#NEUT (x10^3/µL) [a] 53	#NEUT (x10^3/µL) [a1] 87	#NEUT (x10^3/µL) [a] 123	#NEUT (x10^3/µL) [a] 179
1	Mean	42.4	64.1	49.4	55.3	53.9	59.7	5.77	3.76	3.16	3.27	1.99	3.36
0	SD	18.2	26.2	14.0	17.0	21.9	15.5	2.73	1.63	2.45	2.71	0.69	2.67
mg/kg/dose	N	6	6	6	6	6	6	6	6	6	6	6	6
2	Mean	99.0	93.4	44.5	58.5	46.1	57.5	4.19	3.13	2.29	4.60	2.12	1.66
5	SD	98.8	72.6	12.7	23.9	7.1	31.1	2.26	1.56	0.44	3.49	1.58	0.73
mg/kg/dose	N	6	6	6	6	6	6	6	6	6	6	6	6
	%Diff	133.4	45.8	-10.1	5.9	-14.6	-3.7	-27.40	-16.75	-27.58	40.38	6.71	-50.70
3	Mean	58.0	68.3	38.7	76.5	55.9	48.2	4.72	3.66	3.25	2.39	3.31	1.51
25	SD	20.4	25.3	13.2	54.2	15.8	9.9	1.90	2.21	2.35	0.96	2.55	0.50
mg/kg/dose	N	6	6	6	6	6	6	6	6	6	6	6	6
	%Diff	36.8	6.7	-21.7	38.5	3.7	-19.3	-18.31	-2.70	2.96	-27.09	66.78	-54.92
4	Mean	49.3	69.1	60.8	108.5	87.8	93.2	4.39	4.91	6.11	5.65	5.10	2.23
100	SD	17.1	36.8	28.2	126.5	93.6	111.2	2.30	2.90	5.43	5.56	4.81	1.08
mg/kg/dose	N	6	6	6	6	6	6	6	6	6	6	6	6
	%Diff	16.2	7.9	23.0	96.3	62.9	56.1	-23.94	30.48	93.61	72.71	156.88	-33.71

[a] - Anova & Dunnett(Log)
[a1] - Anova & Dunnett

Table 5 Summary of Hematology-Pretest and Dosing Phase (Cont'd)

Day(s) Relative to Start Date

Sex: Male		%NEUT (%) [a] -11	%NEUT (%) [a] -6	%NEUT (%) [a] 53	%NEUT (%) [a] 87	%NEUT (%) [a1] 123	%NEUT (%) [a] 179	#LYMP (x10^3/µL) [a] -11	#LYMP (x10^3/µL) [a] -6	#LYMP (x10^3/µL) [a] 53	#LYMP (x10^3/µL) [a] 87	#LYMP (x10^3/µL) [a] 123	#LYMP (x10^3/µL) [a] 179
1	Mean	52.0	32.8	28.0	30.7	25.2	31.1	4.62	6.90	6.56	5.83	5.39	5.86
0	SD	7.0	8.0	16.1	12.8	8.3	14.6	1.45	2.00	1.43	2.12	0.87	2.10
mg/kg/dose	N	6	6	6	6	6	6	6	6	6	6	6	6
2	Mean	42.6	29.2	24.4	37.5	22.9	20.7	4.89	7.03	6.67	6.02	6.06	6.15
5	SD	15.4	11.6	3.4	16.4	9.0	9.7	2.06	3.27	2.28	1.85	2.13	2.28
mg/kg/dose	N	6	6	6	6	6	6	6	6	6	6	6	6
	%Diff	-18.1	-11.0	-13.0	22.1	-9.0	-33.4	5.92	1.79	1.68	3.20	12.40	4.98
3	Mean	52.7	35.8	33.3	30.4	33.9	20.5	3.73	5.76	5.67	5.15	5.54	5.46
25	SD	18.0	19.3	19.5	12.0	21.2	4.0	1.07	1.87	1.47	1.29	1.88	1.23
mg/kg/dose	N	6	6	6	6	6	6	6	6	6	6	6	6
	%Diff	1.3	9.2	18.8	-1.0	34.9	-34.0	-19.37	-16.59	-13.64	-11.70	2.81	-6.83
4	Mean	50.6	42.7	45.9	48.7	42.7	30.5	3.80	5.80	5.07	4.07	4.56	5.00
100	SD	18.9	15.9	23.3	22.3	24.5	12.8	1.73	2.18	1.60	1.66	1.58	2.03
mg/kg/dose	N	6	6	6	6	6	6	6	6	6	6	6	6
	%Diff	-2.7	30.1	63.7	58.8	69.6	-2.0	-17.71	-15.96	-22.71	-30.11	-15.40	-14.57

[a] - Anova & Dunnett
[a1] - Anova & Dunnett(Log)

Table 5 Summary of Hematology-Pretest and Dosing Phase (Cont'd)

Day(s) Relative to Start Date

Sex: Male		%LYMP (%) [a] -11	%LYMP (%) [a] -6	%LYMP (%) [a] 53	%LYMP (%) [a] 87	%LYMP (%) [a] 123	%LYMP (%) [a1] 179	#MONO (x10^3/µL) [a] -11	#MONO (x10^3/µL) [a] -6	#MONO (x10^3/µL) [a] 53	#MONO (x10^3/µL) [a] 87	#MONO (x10^3/µL) [a] 123	#MONO (x10^3/µL) [a] 179
1	Mean	44.2	60.7	66.3	63.3	68.4	61.6	0.24	0.36	0.28	0.28	0.28	0.34
0	SD	6.3	8.7	14.9	12.4	8.1	14.5	0.16	0.09	0.10	0.12	0.05	0.15
mg/kg/dose	N	6	6	6	6	6	6	6	6	6	6	6	6
2	Mean	52.7	64.6	68.9	57.3	71.7	72.9	0.27	0.37	0.34	0.35	0.28	0.33
5	SD	14.9	11.4	4.3	16.0	8.5	10.1	0.10	0.18	0.12	0.15	0.12	0.11
mg/kg/dose	N	6	6	6	6	6	6	6	6	6	6	6	6
	%Diff	19.3	6.5	4.0	-9.4	4.8	18.2	13.89	1.83	22.16	25.00	1.81	-2.99
3	Mean	44.4	59.4	62.2	64.8	61.4	74.3	0.16	0.29	0.24	0.26	0.29	0.22
25	SD	18.0	19.0	18.6	11.6	21.9	4.1	0.05	0.06	0.08	0.08	0.09	0.05
mg/kg/dose	N	6	6	6	6	6	6	6	6	6	6	6	6
	%Diff	0.6	-2.1	-6.1	2.5	-10.3	20.6	-31.94	-20.18	-13.17	-8.33	4.22	-34.83
4	Mean	45.7	52.4	49.4	46.5	52.8	64.5	0.21	0.28	0.29	0.27	0.26	0.20
100	SD	18.2	15.8	22.5	21.2	23.5	13.0	0.07	0.07	0.11	0.10	0.10	0.05
mg/kg/dose	N	6	6	6	6	6	6	6	6	6	6	6	6
	%Diff	3.5	-13.7	-25.5	-26.6	-22.9	4.7	-14.58	-22.02	5.39	-4.17	-6.63	-40.30

[a] - Anova & Dunnett
[a1] - Kruskal-Wallis & Dunnett on Ranks

Table 5 Summary of Hematology-Pretest and Dosing Phase (Cont'd)

Day(s) Relative to Start Date

Sex: Male		%MONO (%) [a] -11	%MONO (%) [a] -6	%MONO (%) [a] 53	%MONO (%) [a1] 87	%MONO (%) [a] 123	%MONO (%) [a] 179	#EOS (x10^3/µL) [a2] -11	#EOS (x10^3/µL) [a] -6	#EOS (x10^3/µL) [a] 53	#EOS (x10^3/µL) [a] 87	#EOS (x10^3/µL) [a2] 123	#EOS (x10^3/µL) [a] 179
1	Mean	2.2	3.3	2.7	2.9	3.5	3.4	0.08	0.22	0.17	0.18	0.15	0.25
0	SD	0.9	0.6	0.5	0.2	0.4	1.0	0.05	0.20	0.13	0.19	0.16	0.22
mg/kg/dose	N	6	6	6	6	6	6	6	6	6	6	6	6
2	Mean	2.9	3.3	3.6	3.2	3.3	3.9	0.10	0.20	0.16	0.12	0.07	0.14
5	SD	0.8	1.3	1.1	1.2	1.0	1.2	0.12	0.21	0.12	0.06	0.05	0.11
mg/kg/dose	N	6	6	6	6	6	6	6	6	6	6	6	6
	%Diff	28.4	2.6	36.5	9.7	-3.8	14.8	14.00	-12.03	-5.94	-30.48	-51.69	-43.24
3	Mean	1.9	3.0	2.6	3.2	3.1	3.0	0.04	0.10	0.09	0.06	0.05	0.14
25	SD	0.5	0.6	1.0	0.7	0.8	0.6	0.02	0.08	0.08	0.06	0.03	0.20
mg/kg/dose	N	6	6	6	6	6	6	6	6	6	6	6	6
	%Diff	-15.7	-8.2	-0.6	9.7	-9.6	-11.8	-52.00	-55.64	-44.55	-64.76	-67.42	-44.59
4	Mean	2.4	2.7	2.6	2.8	2.7	2.8	0.06	0.16	0.13	0.08	0.08	0.09
100	SD	0.6	0.8	0.3	0.6	0.4	0.7	0.02	0.08	0.09	0.05	0.04	0.04
mg/kg/dose	N	6	6	6	6	6	6	6	6	6	6	6	6
	%Diff	6.7	-18.5	-3.1	-2.9	-23.6	-18.7	-34.00	-26.32	-22.77	-55.24	-46.07	-65.54

[a] - Anova & Dunnett
[a1] - Kruskal-Wallis & Dunnett on Ranks
[a2] - Anova & Dunnett(Log)

Table 5 Summary of Hematology-Pretest and Dosing Phase (Cont'd)

Day(s) Relative to Start Date

Sex: Male		%EOS (%) [a] -11	%EOS (%) [a] -6	%EOS (%) [a] 53	%EOS (%) [a1] 87	%EOS (%) [a] 123	%EOS (%) [a] 179	#BASO (x10^3/μL) [a] -11	#BASO (x10^3/μL) [a2] -6	#BASO (x10^3/μL) [a] 53	#BASO (x10^3/μL) [a] 87	#BASO (x10^3/μL) [a] 123	#BASO (x10^3/μL) [a] 179
1	Mean	0.9	2.1	1.8	2.2	1.9	3.0	0.03	0.04	0.03	0.02	0.02	0.04
0	SD	0.4	1.6	1.6	2.8	2.0	2.9	0.01	0.02	0.01	0.01	0.01	0.02
mg/kg/dose	N	6	6	6	6	6	6	6	6	6	6	6	6
2	Mean	1.0	1.6	1.8	1.1	0.9	1.6	0.03	0.03	0.02	0.02	0.03	0.03
5	SD	0.8	1.0	1.3	0.5	0.6	0.9	0.02	0.03	0.01	0.01	0.02	0.01
mg/kg/dose	N	6	6	6	6	6	6	6	6	6	6	6	6
	%Diff	11.8	-22.0	-4.5	-50.0	-52.6	-47.2	-6.25	-22.73	-26.67	0.00	14.29	-23.81
3	Mean	0.5	1.0	1.0	0.7	0.5	1.6	0.01	0.02	0.02	0.01	0.02	0.02
25	SD	0.2	0.9	1.0	0.6	0.3	2.1	0.01	0.01	0.01	0.01	0.01	0.01
mg/kg/dose	N	6	6	6	6	6	6	6	6	6	6	6	6
	%Diff	-45.1	-50.4	-43.6	-67.2	-71.9	-45.5	-56.25	-40.91	-13.33	-46.15	-21.43	-47.62
4	Mean	0.7	1.6	1.2	1.1	1.2	1.3	0.02	0.02	0.02	0.02	0.02	0.02
100	SD	0.5	0.9	0.9	1.1	1.4	1.0	0.01	0.02	0.01	0.01	0.01	0.01
mg/kg/dose	N	6	6	6	6	6	6	6	6	6	6	6	6
	%Diff	-15.7	-22.8	-33.6	-52.2	-37.7	-56.2	-37.50	-36.36	-6.67	-15.38	-28.57	-38.10

[a] - Anova & Dunnett
[a1] - Anova & Dunnett(Log)
[a2] - Kruskal-Wallis & Dunnett on Ranks

Table 5 Summary of Hematology-Pretest and Dosing Phase (Cont'd)

Day(s) Relative to Start Date

Sex: Male		%BASO (%) [a] -11	%BASO (%) [a] -6	%BASO (%) [a1] 53	%BASO (%) [a1] 87	%BASO (%) [a] 123	%BASO (%) [a] 179	PLT (x10^3/µL) [a] -11	PLT (x10^3/µL) [a] -6	PLT (x10^3/µL) [a] 53	PLT (x10^3/µL) [a] 87	PLT (x10^3/µL) [a] 123	PLT (x10^3/µL) [a] 179
1	Mean	0.3	0.3	0.3	0.3	0.3	0.4	402	410	363	338	330	338
0	SD	0.2	0.1	0.1	0.1	0.1	0.1	57	45	59	36	45	42
mg/kg/dose	N	6	6	6	6	6	6	6	6	6	6	6	6
2	Mean	0.3	0.2	0.2	0.2	0.3	0.3	383	429	358	344	327	311
5	SD	0.1	0.1	0.0	0.0	0.1	0.1	76	74	57	62	52	66
mg/kg/dose	N	6	6	6	6	6	6	6	6	6	6	6	6
	%Diff	0.0	-22.2	-20.0	-20.0	12.5	-14.3	-5	5	-1	2	-1	-8
3	Mean	0.1	0.2	0.2	0.2	0.2	0.2	411	449	371	355	345	359
25	SD	0.1	0.1	0.1	0.1	0.1	0.1	90	100	86	96	95	77
mg/kg/dose	N	6	6	6	6	6	6	6	6	6	6	6	6
	%Diff	-53.3	-27.8	-20.0	-33.3	-25.0	-38.1	2	10	2	5	5	6
4	Mean	0.2	0.2	0.2	0.2	0.2	0.3	396	453	386	352	342	332
100	SD	0.1	0.1	0.1	0.1	0.1	0.1	69	117	80	85	69	78
mg/kg/dose	N	6	6	6	6	6	6	6	6	6	6	6	6
	%Diff	-20.0	-38.9	-20.0	-20.0	-25.0	-9.5	-2	11	6	4	4	-2

[a] - Anova & Dunnett
[a1] - Kruskal-Wallis & Dunnett on Ranks

Table 5 Summary of Hematology-Pretest and Dosing Phase (Cont'd)

Day(s) Relative to Start Date

Sex: Male		MPV (fL) [a]	MPV (fL) [a]	MPV (fL) [a]	MPV (fL) [a]	MPV (fL) [a]	MPV (fL) [a]
		-11	-6	53	87	123	179
1	Mean	8.0	8.0	8.6	8.9	9.5	9.4
0	SD	0.7	1.1	1.5	1.7	1.8	1.9
mg/kg/dose	N	6	6	6	6	6	6
2	Mean	7.8	7.6	8.3	8.3	9.1	9.2
5	SD	1.3	1.2	1.6	1.7	1.8	1.8
mg/kg/dose	N	6	6	6	6	6	6
	%Diff	-2.3	-4.4	-3.5	-6.0	-4.7	-2.5
3	Mean	7.8	7.5	8.1	8.4	9.3	9.2
25	SD	2.0	1.8	2.4	2.4	3.1	2.4
mg/kg/dose	N	6	6	6	6	6	6
	%Diff	-3.1	-5.7	-6.0	-5.8	-2.1	-2.3
4	Mean	7.6	7.6	8.1	8.2	8.9	8.8
100	SD	0.9	0.9	0.9	1.0	1.0	1.0
mg/kg/dose	N	6	6	6	6	6	6
	%Diff	-5.2	-4.4	-6.0	-8.1	-6.1	-6.0

[a] - Anova & Dunnett

Table 5 Summary of Hematology-Pretest and Dosing Phase (Cont'd)

Day(s) Relative to Start Date

Sex: Female		WBC (x10^3/µL) [a] -12	WBC (x10^3/µL) [a1] -7	WBC (x10^3/µL) [a2] 52	WBC (x10^3/µL) [a] 86	WBC (x10^3/µL) [a1] 122	WBC (x10^3/µL) [a1] 178	RBC (x10^6/µL) [a1] -12	RBC (x10^6/µL) [a1] -7	RBC (x10^6/µL) [a1] 52	RBC (x10^6/µL) [a1] 86	RBC (x10^6/µL) [a1] 122	RBC (x10^6/µL) [a1] 178
1	Mean	17.29	13.88	12.96	10.39	10.18	8.66	5.40	5.19	5.37	5.39	5.41	5.31
0	SD	5.12	4.78	2.54	1.97	2.31	2.69	0.17	0.21	0.18	0.37	0.23	0.24
mg/kg/dose	N	6	6	6	6	6	6	6	6	6	6	6	6
2	Mean	16.42	12.45	11.13	11.00	7.33	8.37	5.88	5.61	5.87	5.82	5.11	5.61
5	SD	3.94	1.74	2.92	3.83	3.73	2.07	0.46	0.46	0.56	0.58	2.05	0.47
mg/kg/dose	N	6	6	6	6	6	6	6	6	6	6	6	6
	%Diff	-5.03	-10.31	-14.17	5.92	-27.99	-3.44	8.93	7.96	9.18	7.94	-5.55	5.78
3	Mean	11.73	10.97	7.44	7.51	7.72	7.17	5.57	5.45	5.60	5.38	5.64	5.34
25	SD	2.66	3.03	4.66	3.28	2.53	2.88	0.28	0.30	0.24	0.33	0.38	0.37
mg/kg/dose	N	6	6	6	6	6	6	6	6	6	6	6	6
	%Diff	-32.17	-20.97	-42.63	-27.65	-24.14	-17.22	3.15	4.88	4.25	-0.22	4.35	0.60
4	Mean	13.81	10.30	9.39	11.37	7.02	9.15	5.70	5.58	5.78	5.80	5.92	5.67
100	SD	5.94	4.09	5.30	6.41	3.17	5.00	0.70	0.68	0.31	0.47	0.54	0.60
mg/kg/dose	N	6	6	6	6	6	6	6	6	6	6	6	6
	%Diff	-20.11	-25.83	-27.56	9.52	-31.10	5.64	5.59	7.51	7.51	7.45	9.53	6.85

[a] - Anova & Dunnett(Log)
[a1] - Anova & Dunnett
[a2] - Kruskal-Wallis & Dunnett on Ranks

Table 5 Summary of Hematology-Pretest and Dosing Phase (Cont'd)

Day(s) Relative to Start Date

Sex: Female		HGB (g/dL) [a] -12	HGB (g/dL) [a1] -7	HGB (g/dL) [a2] 52	HGB (g/dL) [a1] 86	HGB (g/dL) [a2] 122	HGB (g/dL) [a1] 178	HCT (%) [a1] -12	HCT (%) [a2] -7	HCT (%) [a2] 52	HCT (%) [a1] 86	HCT (%) [a2] 122	HCT (%) [a1] 178
1	Mean	12.8	12.3	12.8	12.7	13.1	12.7	42.1	40.4	41.7	41.4	41.5	41.6
0	SD	0.9	0.8	0.6	0.9	0.6	0.7	2.4	2.1	1.6	3.2	1.8	2.7
mg/kg/dose	N	6	6	6	6	6	6	6	6	6	6	6	6
2	Mean	13.7	13.0	13.5	13.2	12.0	13.0	44.2	42.5	43.7	43.0	37.7	41.9
5	SD	1.2	1.1	1.4	1.2	4.9	1.1	3.7	3.1	3.8	3.1	14.9	2.8
mg/kg/dose	N	6	6	6	6	6	6	6	6	6	6	6	6
	%Diff	7.2	5.7	5.3	4.5	-8.7	2.4	5.1	5.2	4.8	3.7	-9.2	0.8
3	Mean	13.0	12.9	13.3	12.5	13.4	12.8	43.3	42.9	43.6	41.4	43.5	41.7
25	SD	0.5	0.4	0.4	0.8	0.6	0.8	2.0	1.2	1.3	2.8	2.3	2.7
mg/kg/dose	N	6	6	6	6	6	6	6	6	6	6	6	6
	%Diff	1.8	5.2	3.5	-1.6	2.4	0.7	3.0	6.2	4.5	0.0	4.7	0.2
4	Mean	13.1	12.9	13.4	13.3	13.9	13.3	44.1	43.4	43.9	43.7	44.9	43.7
100	SD	0.8	0.8	0.5	0.4	0.7	0.5	3.3	2.4	1.7	1.9	2.6	3.5
mg/kg/dose	N	6	6	6	6	6	6	6	6	6	6	6	6
	%Diff	3.0	5.2	4.7	4.9	6.0	4.2	4.9	7.4	5.2	5.5	8.2	5.0

[a] - Anova & Dunnett(Log)
[a1] - Anova & Dunnett
[a2] - Kruskal-Wallis & Dunnett on Ranks

Table 5 Summary of Hematology-Pretest and Dosing Phase (Cont'd)

Day(s) Relative to Start Date

Sex: Female		MCV (fL) [a] -12	MCV (fL) [a] -7	MCV (fL) [a] 52	MCV (fL) [a] 86	MCV (fL) [a] 122	MCV (fL) [a1] 178	MCH (pg) [a] -12	MCH (pg) [a] -7	MCH (pg) [a] 52	MCH (pg) [a] 86	MCH (pg) [a] 122	MCH (pg) [a] 178
1	Mean	77.8	77.7	77.7	76.8	76.8	78.4	23.6	23.6	23.9	23.5	24.2	24.0
0	SD	2.9	1.2	2.7	2.3	2.5	2.0	0.9	0.8	0.7	0.8	0.7	0.7
mg/kg/dose	N	6	6	6	6	6	6	6	6	6	6	6	6
2	Mean	75.3	75.9	74.7	74.0	73.9	74.8	23.3	23.2	23.1	22.8	23.2	23.3
5	SD	5.0	4.7	4.6	3.9	4.1	5.4	1.6	1.5	1.5	1.5	2.0	1.8
mg/kg/dose	N	6	6	6	6	6	6	6	6	6	6	6	6
	%Diff	-3.2	-2.3	-3.9	-3.6	-3.8	-4.5	-1.4	-1.6	-3.4	-3.2	-4.4	-2.8
3	Mean	77.9	78.8	77.9	77.0	77.1	78.1	23.4	23.8	23.7	23.2	23.8	24.0
25	SD	3.0	2.8	2.2	2.8	1.7	2.3	0.9	0.9	0.6	1.0	0.7	0.8
mg/kg/dose	N	6	6	6	6	6	6	6	6	6	6	6	6
	%Diff	0.1	1.4	0.3	0.2	0.3	-0.3	-0.8	0.8	-0.9	-1.3	-1.7	0.1
4	Mean	77.9	78.3	76.2	75.7	76.2	77.3	23.2	23.3	23.2	23.0	23.6	23.6
100	SD	5.9	6.6	5.5	5.7	7.1	6.0	1.7	1.8	1.6	1.8	1.9	2.0
mg/kg/dose	N	6	6	6	6	6	6	6	6	6	6	6	6
	%Diff	0.1	0.8	-1.9	-1.4	-0.7	-1.3	-1.7	-1.1	-2.7	-2.1	-2.6	-1.7

[a] - Anova & Dunnett
[a1] - Kruskal-Wallis & Dunnett on Ranks

Table 5 Summary of Hematology-Pretest and Dosing Phase (Cont'd)

Day(s) Relative to Start Date

Sex: Female		MCHC (g/dL) [a] -12	MCHC (g/dL) [a] -7	MCHC (g/dL) [a] 52	MCHC (g/dL) [a1] 86	MCHC (g/dL) [a] 122	MCHC (g/dL) [a] 178	RDW (%) [a] -12	RDW (%) [a] -7	RDW (%) [a] 52	RDW (%) [a] 86	RDW (%) [a1] 122	RDW (%) [a] 178
1	Mean	30.3	30.3	30.7	30.6	31.6	30.6	13.3	13.4	13.0	12.9	12.6	13.3
0	SD	1.0	0.7	1.1	0.6	1.1	0.9	0.7	0.7	0.6	0.6	0.6	0.6
mg/kg/dose	N	6	6	6	6	6	6	6	6	6	6	6	6
2	Mean	30.9	30.6	30.9	30.7	31.3	31.1	13.0	13.0	12.8	12.8	13.7	13.3
5	SD	0.4	0.4	0.7	1.0	1.4	0.9	0.3	0.2	0.3	0.3	3.5	0.6
mg/kg/dose	N	6	6	6	6	6	6	6	6	6	6	6	6
	%Diff	1.9	0.8	0.5	0.4	-0.7	1.6	-1.9	-3.0	-1.4	-0.9	9.0	-0.4
3	Mean	30.0	30.2	30.4	30.2	30.9	30.8	13.1	13.1	12.8	12.9	12.4	13.1
25	SD	0.8	0.8	0.8	1.2	0.9	0.8	0.5	0.4	0.6	0.4	0.4	0.6
mg/kg/dose	N	6	6	6	6	6	6	6	6	6	6	6	6
	%Diff	-0.9	-0.5	-1.0	-1.3	-2.0	0.4	-1.5	-2.2	-1.4	-0.5	-1.2	-2.0
4	Mean	29.8	29.8	30.5	30.4	31.0	30.5	13.8	13.7	13.0	13.1	12.6	13.5
100	SD	0.6	0.6	0.4	0.6	0.7	1.1	0.6	0.5	0.5	0.8	0.6	0.6
mg/kg/dose	N	6	6	6	6	6	6	6	6	6	6	6	6
	%Diff	-1.7	-1.9	-0.8	-0.7	-1.9	-0.3	3.8	2.4	0.1	1.4	0.0	1.0

[a] - Anova & Dunnett
[a1] - Kruskal-Wallis & Dunnett on Ranks

Table 5 Summary of Hematology-Pretest and Dosing Phase (Cont'd)

Day(s) Relative to Start Date

Sex: Female		#RET (x10^9/L) [a] -12	#RET (x10^9/L) [a] -7	#RET (x10^9/L) [a] 52	#RET (x10^9/L) [a] 86	#RET (x10^9/L) [a] 122	#RET (x10^9/L) [a1] 178	#NEUT (x10^3/μL) [a2] -12	#NEUT (x10^3/μL) [a] -7	#NEUT (x10^3/μL) [a] 52	#NEUT (x10^3/μL) [a] 86	#NEUT (x10^3/μL) [a] 122	#NEUT (x10^3/μL) [a] 178
1	Mean	52.9	69.1	47.0	56.0	69.8	55.9	12.19	8.74	7.70	6.41	5.37	4.11
0	SD	6.2	18.5	12.1	17.7	16.7	8.4	4.74	4.48	2.66	1.88	2.78	3.03
mg/kg/dose	N	6	6	6	6	6	6	6	6	6	6	6	6
2	Mean	57.2	81.6	67.3	63.3	77.6	70.1	11.44	7.07	6.33	7.03	3.34	3.66
5	SD	19.9	31.6	14.9	16.6	16.8	14.7	4.41	2.12	2.91	4.13	3.62	1.25
mg/kg/dose	N	6	6	6	6	6	6	6	6	6	6	6	6
	%Diff	8.2	18.0	43.2	13.0	11.1	25.4	-6.22	-19.14	-17.77	9.65	-37.85	-10.98
3	Mean	45.6	66.5	49.3	53.9	64.3	61.6	7.04	5.79	2.92	3.71	3.01	2.24
25	SD	8.4	20.7	16.0	16.9	12.2	17.9	2.04	2.50	2.52	2.27	1.51	1.03
mg/kg/dose	N	6	6	6	6	6	6	6	6	6	6	6	6
	%Diff	-13.8	-3.8	4.8	-3.7	-7.9	10.1	-42.26	-33.78	-62.07	-42.10	-43.93	-45.54
4	Mean	75.9	92.2	57.6	59.4	67.5	64.9	9.59	5.21	4.57	7.18	2.82	4.71
100	SD	38.4	43.7	21.0	23.3	21.6	26.5	5.27	2.37	3.61	4.49	2.23	2.88
mg/kg/dose	N	6	6	6	6	6	6	6	6	6	6	6	6
	%Diff	43.6	33.4	22.5	6.1	-3.3	16.1	-21.36	-40.43	-40.68	11.96	-47.53	14.47

[a] - Anova & Dunnett
[a1] - Anova & Dunnett(Log)
[a2] - Kruskal-Wallis & Dunnett on Ranks

Table 5 Summary of Hematology-Pretest and Dosing Phase (Cont'd)

Day(s) Relative to Start Date

Sex: Female		%NEUT (%) [a] -12	%NEUT (%) [a] -7	%NEUT (%) [a] 52	%NEUT (%) [a] 86	%NEUT (%) [a] 122	%NEUT (%) [a] 178	#LYMP (x10^3/µL) [a] -12	#LYMP (x10^3/µL) [a] -7	#LYMP (x10^3/µL) [a] 52	#LYMP (x10^3/µL) [a] 86	#LYMP (x10^3/µL) [a] 122	#LYMP (x10^3/µL) [a] 178
1	Mean	69.0	60.2	58.3	61.0	50.0	44.4	4.55	4.49	4.68	3.55	4.31	4.05
0	SD	8.6	13.5	10.3	8.5	17.1	18.4	1.02	1.08	1.09	0.62	0.84	1.07
mg/kg/dose	N	6	6	6	6	6	6	6	6	6	6	6	6
2	Mean	67.9	56.2	55.1	61.2	38.9	44.1	4.32	4.70	4.30	3.56	3.65	4.34
5	SD	12.3	12.7	12.1	12.5	19.5	10.2	1.45	1.22	0.90	0.91	1.16	1.39
mg/kg/dose	N	6	6	6	6	6	6	6	6	6	6	6	6
	%Diff	-1.6	-6.7	-5.5	0.2	-22.3	-0.8	-5.02	4.75	-8.08	0.23	-15.33	6.99
3	Mean	59.5	51.0	35.3*	47.3	38.2	31.4	4.17	4.71	4.07	3.42	4.31	4.52
25	SD	8.1	11.8	13.9	15.5	13.5	9.6	1.16	1.15	2.48	1.51	1.82	1.93
mg/kg/dose	N	6	6	6	6	6	6	6	6	6	6	6	6
	%Diff	-13.8	-15.3	-39.4	-22.4	-23.7	-29.4	-8.32	4.79	-13.07	-3.66	0.08	11.56
4	Mean	67.8	50.7	42.9	60.9	37.0	49.4	3.76	4.58	4.30	3.76	3.87	4.04
100	SD	14.7	10.7	15.1	12.6	14.3	12.8	1.86	2.10	1.73	1.87	1.72	2.14
mg/kg/dose	N	6	6	6	6	6	6	6	6	6	6	6	6
	%Diff	-1.8	-15.8	-26.5	-0.2	-26.0	11.1	-17.41	2.08	-8.19	5.82	-10.10	-0.29

[a] - Anova & Dunnett: * = $p \leq 0.05$

Table 5 Summary of Hematology-Pretest and Dosing Phase (Cont'd)

Day(s) Relative to Start Date

Sex: Female		%LYMP (%) [a] -12	%LYMP (%) [a] -7	%LYMP (%) [a] 52	%LYMP (%) [a] 86	%LYMP (%) [a] 122	%LYMP (%) [a] 178	#MONO (x10^3/µL) [a] -12	#MONO (x10^3/µL) [a] -7	#MONO (x10^3/µL) [a] 52	#MONO (x10^3/µL) [a] 86	#MONO (x10^3/µL) [a] 122	#MONO (x10^3/µL) [a] 178
1	Mean	27.8	35.1	37.1	35.0	45.2	49.6	0.27	0.30	0.37	0.28	0.33	0.31
0	SD	8.4	13.3	9.4	7.7	17.0	16.6	0.10	0.17	0.19	0.13	0.26	0.18
mg/kg/dose	N	6	6	6	6	6	6	6	6	6	6	6	6
2	Mean	27.7	38.3	40.1	34.9	56.0	51.4	0.33	0.25	0.33	0.29	0.22	0.26
5	SD	10.6	10.8	10.8	12.0	19.1	9.8	0.11	0.07	0.11	0.07	0.08	0.08
mg/kg/dose	N	6	6	6	6	6	6	6	6	6	6	6	6
	%Diff	-0.5	9.0	8.3	-0.2	23.9	3.5	23.60	-15.73	-10.45	2.99	-35.50	-18.09
3	Mean	35.7	44.1	58.0*	47.3	56.4	62.7	0.27	0.26	0.29	0.26	0.27	0.30
25	SD	6.2	9.1	13.1	14.7	13.2	8.5	0.10	0.11	0.15	0.12	0.07	0.15
mg/kg/dose	N	6	6	6	6	6	6	6	6	6	6	6	6
	%Diff	28.4	25.6	56.5	35.3	25.0	26.4	0.62	-13.48	-21.82	-7.19	-20.00	-5.85
4	Mean	28.8	44.3	51.1	35.1	57.7	45.7	0.24	0.23	0.23	0.25	0.19	0.27
100	SD	13.7	10.2	13.8	11.4	13.0	11.9	0.11	0.07	0.07	0.13	0.06	0.11
mg/kg/dose	N	6	6	6	6	6	6	6	6	6	6	6	6
	%Diff	3.6	26.1	37.8	0.3	27.8	-7.9	-9.94	-23.60	-37.73	-10.18	-42.50	-14.36

[a] - Anova & Dunnett: * = p ≤ 0.05

Table 5 Summary of Hematology-Pretest and Dosing Phase (Cont'd)

Day(s) Relative to Start Date

Sex: Female		%MONO (%) [a] -12	%MONO (%) [a1] -7	%MONO (%) [a] 52	%MONO (%) [a] 86	%MONO (%) [a] 122	%MONO (%) [a] 178	#EOS (x10^3/µL) [a] -12	#EOS (x10^3/µL) [a] -7	#EOS (x10^3/µL) [a1] 52	#EOS (x10^3/µL) [a1] 86	#EOS (x10^3/µL) [a] 122	#EOS (x10^3/µL) [a] 178
1	Mean	1.6	2.1	2.9	2.6	3.1	3.6	0.18	0.27	0.13	0.08	0.10	0.13
0	SD	0.4	0.6	1.4	1.0	1.7	1.9	0.12	0.33	0.05	0.04	0.04	0.13
mg/kg/dose	N	6	6	6	6	6	6	6	6	6	6	6	6
2	Mean	2.1	2.0	3.2	2.7	3.2	3.2	0.23	0.33	0.08	0.06	0.07	0.05
5	SD	0.9	0.6	1.3	0.9	0.9	1.0	0.18	0.32	0.07	0.03	0.08	0.04
mg/kg/dose	N	6	6	6	6	6	6	6	6	6	6	6	6
	%Diff	36.6	-2.4	9.2	3.2	2.7	-11.5	27.78	22.64	-38.46	-19.15	-30.51	-58.97
3	Mean	2.5	2.7	4.2	3.6	3.6	4.2	0.14	0.14	0.09	0.05	0.07	0.05
25	SD	1.3	1.9	1.3	1.4	1.1	1.7	0.13	0.07	0.06	0.04	0.02	0.03
mg/kg/dose	N	6	6	6	6	6	6	6	6	6	6	6	6
	%Diff	58.1	29.0	46.2	38.0	16.8	15.2	-22.22	-49.06	-33.33	-40.43	-33.90	-58.97
4	Mean	1.8	2.3	3.0	2.3	3.2	3.2	0.14	0.18	0.21	0.12	0.06	0.07
100	SD	0.5	0.5	1.7	0.7	1.4	1.2	0.11	0.18	0.28	0.12	0.06	0.06
mg/kg/dose	N	6	6	6	6	6	6	6	6	6	6	6	6
	%Diff	15.1	12.1	4.6	-12.7	2.2	-11.1	-20.37	-32.70	60.26	46.81	-40.68	-47.44

[a] - Anova & Dunnett
[a1] - Anova & Dunnett(Log)

Table 5 Summary of Hematology-Pretest and Dosing Phase (Cont'd)

Day(s) Relative to Start Date

Sex: Female		%EOS (%) [a] -12	%EOS (%) [a] -7	%EOS (%) [a] 52	%EOS (%) [a1] 86	%EOS (%) [a2] 122	%EOS (%) [a] 178	#BASO (x10^3/µL) [a] -12	#BASO (x10^3/µL) [a] -7	#BASO (x10^3/µL) [a] 52	#BASO (x10^3/µL) [a2] 86	#BASO (x10^3/µL) [a] 122	#BASO (x10^3/µL) [a] 178
1	Mean	1.0	1.9	1.1	0.8	1.0	1.7	0.03	0.02	0.02	0.02	0.02	0.02
0	SD	0.5	2.1	0.5	0.3	0.5	1.8	0.01	0.01	0.01	0.01	0.01	0.01
mg/kg/dose	N	6	6	6	6	6	6	6	6	6	6	6	6
2	Mean	1.6	2.7	0.8	0.6	1.0	0.6	0.03	0.02	0.02	0.02	0.02	0.02
5	SD	1.4	2.7	0.8	0.3	1.2	0.5	0.02	0.01	0.01	0.01	0.01	0.02
mg/kg/dose	N	6	6	6	6	6	6	6	6	6	6	6	6
	%Diff	56.5	40.5	-25.0	-17.8	-8.1	-64.7	13.33	18.18	7.69	11.11	-10.00	40.00
3	Mean	1.3	1.4	1.4	0.6	0.8	0.7	0.02	0.02	0.02	0.01	0.02	0.02
25	SD	1.4	1.0	1.2	0.4	0.3	0.2	0.01	0.01	0.02	0.01	0.01	0.01
mg/kg/dose	N	6	6	6	6	6	6	6	6	6	6	6	6
	%Diff	29.0	-27.6	32.8	-24.4	-21.0	-56.9	-13.33	9.09	-7.69	-11.11	10.00	10.00
4	Mean	1.0	1.6	1.9	1.2	0.9	1.0	0.02	0.02	0.02	0.02	0.02	0.02
100	SD	0.7	1.5	1.9	1.4	0.9	1.2	0.01	0.01	0.01	0.02	0.01	0.02
mg/kg/dose	N	6	6	6	6	6	6	6	6	6	6	6	6
	%Diff	-8.1	-16.4	78.1	60.0	-12.9	-43.1	-26.67	0.00	-15.38	22.22	0.00	20.00

[a] - Anova & Dunnett
[a1] - Anova & Dunnett(Log)
[a2] - Kruskal-Wallis & Dunnett on Ranks

Table 5 Summary of Hematology-Pretest and Dosing Phase (Cont'd)

Day(s) Relative to Start Date

Sex: Female		%BASO (%) [a] -12	%BASO (%) [a] -7	%BASO (%) [a] 52	%BASO (%) [a] 86	%BASO (%) [a] 122	%BASO (%) [a] 178	PLT (x10^3/µL) [a1] -12	PLT (x10^3/µL) [a] -7	PLT (x10^3/µL) [a] 52	PLT (x10^3/µL) [a] 86	PLT (x10^3/µL) [a] 122	PLT (x10^3/µL) [a] 178
1	Mean	0.2	0.2	0.2	0.2	0.2	0.2	558	539	459	420	419	428
0	SD	0.1	0.1	0.1	0.1	0.1	0.1	71	118	89	78	91	59
mg/kg/dose	N	6	6	6	6	6	6	6	6	6	6	6	6
2	Mean	0.2	0.2	0.2	0.2	0.2	0.3	421 *	433	362	324	308	345
5	SD	0.1	0.0	0.1	0.1	0.1	0.2	141	97	92	67	124	73
mg/kg/dose	N	6	6	6	6	6	6	6	6	6	6	6	6
	%Diff	22.2	22.2	33.3	-10.0	0.0	14.3	-25	-20	-21	-23	-26	-20
3	Mean	0.2	0.2	0.3	0.2	0.3	0.2	423 *	485	388	358	397	352
25	SD	0.0	0.1	0.1	0.1	0.1	0.1	60	109	57	39	53	52
mg/kg/dose	N	6	6	6	6	6	6	6	6	6	6	6	6
	%Diff	22.2	22.2	66.7	0.0	25.0	0.0	-24	-10	-16	-15	-5	-18
4	Mean	0.2	0.2	0.2	0.2	0.2	0.2	422 *	437	372	355	373	365
100	SD	0.1	0.1	0.0	0.1	0.1	0.1	96	81	58	85	72	70
mg/kg/dose	N	6	6	6	6	6	6	6	6	6	6	6	6
	%Diff	11.1	0.0	44.4	-10.0	8.3	-14.3	-24	-19	-19	-16	-11	-15

[a] - Anova & Dunnett
[a1] - Kruskal-Wallis & Dunnett on Ranks: * = p ≤ 0.05

Table 5 Summary of Hematology-Pretest and Dosing Phase (Cont'd)

Day(s) Relative to Start Date

Sex: Female		MPV (fL) [a] -12	MPV (fL) [a] -7	MPV (fL) [a] 52	MPV (fL) [a] 86	MPV (fL) [a] 122	MPV (fL) [a] 178
1	Mean	7.1	7.1	7.8	7.8	8.7	8.5
0	SD	0.7	0.8	1.1	1.0	1.6	1.0
mg/kg/dose	N	6	6	6	6	6	6
2	Mean	7.8	7.4	8.5	8.3	9.1	9.4
5	SD	0.8	0.7	1.0	0.9	0.6	1.0
mg/kg/dose	N	6	6	6	6	6	6
	%Diff	9.1	4.2	8.3	7.1	4.2	10.4
3	Mean	7.7	7.6	8.8	8.6	9.2	9.6
25	SD	0.6	0.5	1.2	0.7	0.9	0.7
mg/kg/dose	N	6	6	6	6	6	6
	%Diff	8.2	6.8	12.8	10.3	5.5	12.1
4	Mean	8.0	7.8	8.4	8.7	9.5	9.7
100	SD	0.8	0.7	0.8	0.9	1.3	0.9
mg/kg/dose	N	6	6	6	6	6	6
	%Diff	11.7	9.9	7.7	11.1	9.2	13.3

[a] - Anova & Dunnett

Table 5 Summary of Hematology-Recovery Phase

Day: 209 Relative to Start Date

Sex: Female		WBC (x10^3/µL)	RBC (x10^6/µL)	HGB (g/dL)	HCT (%)	MCV (fL)	MCH (pg)	MCHC (g/dL)	RDW (%)	#RET (x10^9/L)	#NEUT (x10^3/µL)	%NEUT (%)	#LYMP (x10^3/µL)
1	Mean	9.77	5.25	12.9	43.0	82.0	24.7	30.1	13.5	68.6	4.69	49.5	4.68
0	SD	1.29	0.04	0.4	0.1	1.1	1.1	0.9	0.7	3.4	1.50	21.8	2.64
mg/kg/dose	N	2	2	2	2	2	2	2	2	2	2	2	2
2	Mean	10.63	6.20	14.2	44.8	72.2	22.8	31.6	14.0	65.9	4.44	41.9	5.64
5	SD	3.19	0.49	1.6	4.2	1.0	0.8	0.6	0.6	10.2	1.24	0.8	1.68
mg/kg/dose	N	2	2	2	2	2	2	2	2	2	2	2	2
	%Diff	8.75	18.00	9.7	4.1	-11.9	-7.5	5.0	3.3	-3.9	-5.33	-15.3	20.64
3	Mean	5.35	5.48	13.3	43.9	80.3	24.3	30.3	12.7	72.2	2.08	39.0	2.84
25	SD	0.25	0.47	1.3	3.1	1.3	0.1	0.6	0.1	7.1	0.16	4.9	0.29
mg/kg/dose	N	2	2	2	2	2	2	2	2	2	2	2	2
	%Diff	-45.29	4.29	3.1	2.1	-2.0	-1.4	0.7	-6.3	5.2	-55.76	-21.2	-39.36
4	Mean	6.25	5.63	13.3	44.1	78.3	23.7	30.2	13.3	60.7	3.13	49.6	2.90
100	SD	1.96	0.21	0.7	3.2	2.8	0.2	0.7	0.3	16.1	1.14	2.7	0.88
mg/kg/dose	N	2	2	2	2	2	2	2	2	2	2	2	2
	%Diff	-36.08	7.14	3.1	2.4	-4.5	-4.1	0.5	-1.5	-11.6	-33.37	0.3	-37.97

Table 5 Summary of Hematology-Recovery Phase (Cont'd)

Day: 209 Relative to Start Date

Sex: Female		%LYMP (%)	#MONO (x10^3/µL)	%MONO (%)	#EOS (x10^3/µL)	%EOS (%)	#BASO (x10^3/µL)	%BASO (%)	PLT (x10^3/µL)	MPV (fL)
1	Mean	46.5	0.25	2.6	0.11	1.0	0.03	0.2	387	8.4
0	SD	20.9	0.04	0.1	0.08	0.7	0.01	0.0	87	0.4
mg/kg/dose	N	2	2	2	2	2	2	2	2	2
2	Mean	53.1	0.42	3.9	0.07	0.6	0.04	0.4	352	9.0
5	SD	0.1	0.16	0.4	0.06	0.4	0.03	0.1	126	0.4
mg/kg/dose	N	2	2	2	2	2	2	2	2	2
	%Diff	14.2	66.00	51.0	-38.10	-40.0	60.00	100.0	-9	6.5
3	Mean	53.0	0.35	6.4	0.05	0.9	0.02	0.3	359	8.4
25	SD	3.1	0.15	2.4	0.03	0.6	0.01	0.1	52	0.4
mg/kg/dose	N	2	2	2	2	2	2	2	2	2
	%Diff	14.0	38.00	151.0	-52.38	-10.0	-40.00	25.0	-7	0.0
4	Mean	46.5	0.15	2.7	0.03	0.5	0.01	0.2	318	9.5
100	SD	0.5	0.01	1.1	0.02	0.6	0.00	0.1	1	0.2
mg/kg/dose	N	2	2	2	2	2	2	2	2	2
	%Diff	-0.1	-40.00	3.9	-76.19	-50.0	-60.00	-25.0	-18	12.5

Table 5 Summary of Hematology-Recovery Phase (Cont'd)

Day: 210 Relative to Start Date

Sex: Male		WBC (x10^3/µL)	RBC (x10^6/µL)	HGB (g/dL)	HCT (%)	MCV (fL)	MCH (pg)	MCHC (g/dL)	RDW (%)	#RET (x10^9/L)	#NEUT (x10^3/µL)	%NEUT (%)	#LYMP (x10^3/µL)
1	Mean	11.16	5.88	13.6	45.8	78.0	23.1	29.6	14.4	74.2	3.94	35.0	6.22
0	SD	1.02	0.11	0.4	2.3	2.5	0.1	0.8	0.3	22.1	1.07	6.4	0.45
mg/kg/dose	N	2	2	2	2	2	2	2	2	2	2	2	2
2	Mean	10.94	5.62	12.9	42.2	75.0	22.9	30.5	13.8	84.4	2.60	23.9	7.72
5	SD	0.74	0.07	0.4	0.8	2.4	1.0	0.3	0.5	13.5	0.42	5.5	1.32
mg/kg/dose	N	2	2	2	2	2	2	2	2	2	2	2	2
	%Diff	-2.02	-4.34	-5.2	-8.0	-3.8	-0.7	3.0	-4.5	13.7	-34.14	-31.6	24.14
3	Mean	7.96	5.60	14.0	45.2	80.7	25.0	30.9	14.3	61.5	2.37	30.3	5.26
25	SD	0.46	0.01	0.3	1.1	1.8	0.5	0.1	0.4	7.9	1.26	17.5	1.55
mg/kg/dose	N	2	2	2	2	2	2	2	2	2	2	2	2
	%Diff	-28.72	-4.68	3.3	-1.4	3.5	8.2	4.4	-0.7	-17.1	-39.85	-13.3	-15.45
4	Mean	9.45	5.90	14.0	45.2	76.9	23.7	30.9	13.9	51.6	3.68	39.1	5.44
100	SD	0.09	0.98	1.6	5.6	3.5	1.3	0.3	0.3	5.1	2.36	25.5	2.46
mg/kg/dose	N	2	2	2	2	2	2	2	2	2	2	2	2
	%Diff	-15.37	0.34	3.0	-1.4	-1.4	2.8	4.4	-3.5	-30.5	-6.60	11.9	-12.47

Table 5 Summary of Hematology-Recovery Phase (Cont'd)

Day: 210 Relative to Start Date

Sex: Male		%LYMP (%)	#MONO (x10^3/μL)	%MONO (%)	#EOS (x10^3/μL)	%EOS (%)	#BASO (x10^3/μL)	%BASO (%)	PLT (x10^3/μL)	MPV (fL)
1	Mean	55.8	0.37	3.3	0.55	5.2	0.03	0.3	356	8.4
0	SD	1.1	0.08	0.4	0.61	5.9	0.00	0.0	35	0.4
mg/kg/dose	N	2	2	2	2	2	2	2	2	2
2	Mean	70.4	0.41	3.8	0.14	1.3	0.04	0.3	338	8.8
5	SD	7.3	0.11	1.2	0.08	0.8	0.01	0.0	78	2.8
mg/kg/dose	N	2	2	2	2	2	2	2	2	2
	%Diff	26.2	9.46	13.6	-75.45	-75.7	16.67	0.0	-5	4.8
3	Mean	65.6	0.27	3.3	0.04	0.4	0.02	0.2	273	12.1
25	SD	15.7	0.15	1.7	0.01	0.1	0.01	0.0	78	3.7
mg/kg/dose	N	2	2	2	2	2	2	2	2	2
	%Diff	17.7	-28.38	0.0	-93.64	-93.2	-50.00	-33.3	-23	44.0
4	Mean	57.5	0.18	1.9	0.08	0.8	0.03	0.3	305	8.6
100	SD	25.5	0.01	0.1	0.03	0.3	0.00	0.0	40	1.5
mg/kg/dose	N	2	2	2	2	2	2	2	2	2
	%Diff	3.1	-51.35	-42.4	-85.45	-84.5	0.00	0.0	-14	1.8

Table 6 Summary of Coagulation

Table 6 Summary of Coagulation

Key Page

Measurement Descriptions

Headings Used	Description
PT	Prothrombin Time
APTT	Activated Partial Thromboplastin Time
FIB	Fibrinogen

Table 6 Summary of Coagulation-Pretest and Dosing Phase

Day(s) Relative to Start Date

Sex: Male		PT (Sec) [a] -11	PT (Sec) [a] -6	PT (Sec) [a] 53	PT (Sec) [a] 87	PT (Sec) [a] 123	PT (Sec) [a] 179	APTT (Sec) [a] -11	APTT (Sec) [a1] -6	APTT (Sec) [a] 53	APTT (Sec) [a] 87	APTT (Sec) [a] 123	APTT (Sec) [a] 179
1	Mean	11.2	10.0	12.7	11.9	13.9	14.1	17.5	17.7	17.5	17.6	17.8	18.0
0	SD	0.4	0.8	2.9	1.2	2.0	1.9	0.6	1.0	0.9	1.3	1.4	1.4
mg/kg/dose	N	6	6	6	6	6	6	6	6	6	6	6	6
2	Mean	11.0	10.4	11.1	11.1	13.0	11.7*	17.9	18.0	18.3	18.3	18.9	18.7
5	SD	1.0	0.9	0.9	0.6	1.3	1.1	1.0	0.9	0.6	0.9	1.0	1.2
mg/kg/dose	N	6	6	6	6	6	6	6	6	6	6	6	6
	%Diff	-2.2	4.2	-12.0	-6.9	-6.8	-16.8	1.8	2.1	4.5	3.9	6.1	3.7
3	Mean	11.6	10.3	12.0	13.5	14.0	12.7	18.2	18.4	18.0	18.3	19.6	18.2
25	SD	1.3	0.6	1.7	3.5	1.7	1.1	1.3	1.4	1.1	1.3	2.3	1.6
mg/kg/dose	N	6	6	6	6	6	6	6	6	6	6	6	6
	%Diff	3.0	2.7	-5.1	13.7	0.5	-9.6	3.9	4.2	3.0	4.0	10.3	1.1
4	Mean	11.2	10.3	11.7	11.7	15.1	12.2	17.4	17.4	17.2	17.5	18.0	17.4
100	SD	0.4	0.5	1.6	1.2	3.0	1.3	1.6	2.2	1.7	1.6	1.6	1.7
mg/kg/dose	N	6	6	6	6	6	6	6	6	6	6	6	6
	%Diff	0.1	3.3	-7.9	-1.3	8.5	-13.6	-1.0	-1.2	-1.6	-0.3	1.4	-3.2

[a] - Anova & Dunnett: * = $p \leq 0.05$
[a1] - Kruskal-Wallis & Dunnett on Ranks

Table 6 Summary of Coagulation-Pretest and Dosing Phase (Cont'd)

Day(s) Relative to Start Date

Sex: Male		FIB (g/L) [a] -11	FIB (g/L) [a] -6	FIB (g/L) [a] 53	FIB (g/L) [a1] 87	FIB (g/L) [a1] 123	FIB (g/L) [a2] 179
1	Mean	2.20	2.64	2.35	2.30	1.97	2.64
0	SD	0.45	0.49	0.59	0.52	0.45	0.64
mg/kg/dose	N	6	6	6	6	6	6
2	Mean	2.32	2.53	2.24	2.35	1.94	2.30
5	SD	0.55	0.50	0.30	0.60	0.39	0.38
mg/kg/dose	N	6	6	6	6	6	6
	%Diff	5.47	-4.05	-4.47	2.03	-1.78	-12.82
3	Mean	2.53	3.08	2.51	2.47	3.18	2.66
25	SD	0.18	0.62	0.35	0.31	2.47	0.15
mg/kg/dose	N	6	6	6	6	6	6
	%Diff	15.34	16.95	6.82	7.02	61.25	0.88
4	Mean	2.61	2.63	2.45	2.40	1.90	2.24
100	SD	0.62	0.30	0.51	0.92	0.53	0.34
mg/kg/dose	N	6	6	6	6	6	6
	%Diff	18.68	-0.32	4.40	4.20	-3.72	-15.28

[a] - Anova & Dunnett
[a1] - Anova & Dunnett(Log)
[a2] - Kruskal-Wallis & Dunnett on Ranks

Table 6 Summary of Coagulation-Pretest and Dosing Phase (Cont'd)

Day(s) Relative to Start Date

Sex: Female		PT (Sec) [a] -12	PT (Sec) [a1] -7	PT (Sec) [a] 52	PT (Sec) [a1] 86	PT (Sec) [a1] 122	PT (Sec) [a1] 178	APTT (Sec) [a1] -12	APTT (Sec) [a1] -7	APTT (Sec) [a1] 52	APTT (Sec) [a1] 86	APTT (Sec) [a1] 122	APTT (Sec) [a1] 178
1	Mean	11.0	10.0	10.3	11.9	13.2	12.5	18.2	17.9	17.8	18.2	18.3	17.9
0	SD	0.8	0.3	0.6	1.5	1.0	1.6	1.3	1.3	1.0	1.5	1.1	0.9
mg/kg/dose	N	6	6	6	6	6	6	6	6	6	6	6	6
2	Mean	11.2	10.1	10.3	11.7	12.5	11.0	18.9	18.5	18.0	18.7	19.0	18.7
5	SD	1.4	0.9	1.5	1.1	2.2	1.1	2.0	2.1	2.1	1.8	1.9	2.2
mg/kg/dose	N	6	6	6	6	6	6	6	6	6	6	6	6
	%Diff	2.1	0.8	0.3	-2.4	-4.8	-12.2	4.2	3.4	1.1	2.7	3.5	4.4
3	Mean	11.2	10.0	10.7	12.2	14.0	12.7	17.7	16.8	16.7	17.3	17.7	17.1
25	SD	1.2	0.7	0.4	1.3	1.4	2.5	1.1	1.3	1.2	1.5	1.2	1.3
mg/kg/dose	N	6	6	6	6	6	6	6	6	6	6	6	6
	%Diff	2.3	0.2	3.6	1.8	6.1	2.1	-2.8	-6.4	-6.2	-5.2	-3.5	-4.4
4	Mean	12.6	10.4	10.9	12.3	16.2	14.4	18.0	17.7	17.4	18.1	18.7	17.5
100	SD	2.3	1.0	0.6	0.8	5.7	3.1	1.7	1.9	1.8	2.0	1.9	2.0
mg/kg/dose	N	6	6	6	6	6	6	6	6	6	6	6	6
	%Diff	14.4	4.0	5.5	2.9	22.7	15.2	-1.1	-1.4	-2.4	-0.5	1.9	-2.4

[a] - Kruskal-Wallis & Dunnett on Ranks
[a1] - Anova & Dunnett

Table 6 Summary of Coagulation-Pretest and Dosing Phase (Cont'd)

Day(s) Relative to Start Date

Sex: Female		FIB (g/L) [a]	FIB (g/L) [a]	FIB (g/L) [a]	FIB (g/L) [a]	FIB (g/L) [a]	FIB (g/L) [a]
		-12	-7	52	86	122	178
1	Mean	2.35	2.38	2.59	2.28	2.03	2.28
0	SD	0.31	0.46	0.56	0.48	0.43	0.50
mg/kg/dose	N	6	6	6	6	6	6
2	Mean	2.41	2.35	2.46	2.31	2.10	2.25
5	SD	0.58	0.46	0.30	0.60	0.57	0.46
mg/kg/dose	N	6	6	6	6	6	6
	%Diff	2.63	-1.26	-4.96	1.39	3.70	-1.39
3	Mean	2.39	2.12	2.25	1.93	1.87	2.14
25	SD	0.97	0.34	0.55	0.59	0.49	0.43
mg/kg/dose	N	6	6	6	6	6	6
	%Diff	1.85	-10.93	-13.07	-15.37	-7.90	-6.22
4	Mean	2.10	2.33	2.18	1.97	1.90	2.14
100	SD	0.54	0.55	0.31	0.24	0.26	0.42
mg/kg/dose	N	6	6	6	6	6	6
	%Diff	-10.37	-2.24	-15.65	-13.40	-6.01	-5.93

[a] - Anova & Dunnett

Table 6 Summary of Coagulation-Recovery Phase

Day: 209 Relative to Start Date

Sex: Female		PT (Sec)	APTT (Sec)	FIB (g/L)
1	Mean	16.9	17.0	2.21
0	SD	0.6	0.1	0.21
mg/kg/dose	N	2	2	2
2	Mean	13.9	19.0	1.84
5	SD	0.8	0.9	0.32
mg/kg/dose	N	2	2	2
	%Diff	-17.8	11.5	-16.78
3	Mean	18.0	18.2	2.10
25	SD	1.0	1.3	0.91
mg/kg/dose	N	2	2	2
	%Diff	6.5	6.8	-4.99
4	Mean	14.0	20.1	2.39
100	SD	0.6	1.3	0.24
mg/kg/dose	N	2	2	2
	%Diff	-17.5	17.9	8.39

Table 6 Summary of Coagulation-Recovery Phase (Cont'd)

Day: 210 Relative to Start Date

Sex: Male		PT (Sec)	APTT (Sec)	FIB (g/L)
1	Mean	19.4	18.3	2.53
0	SD	1.3	0.1	0.18
mg/kg/dose	N	2	2	2
2	Mean	14.3	19.6	2.84
5	SD	1.3	0.1	0.30
mg/kg/dose	N	2	2	2
	%Diff	-26.4	7.1	12.28
3	Mean	18.1	18.1	2.68
25	SD	1.6	1.0	0.32
mg/kg/dose	N	2	2	2
	%Diff	-6.5	-0.8	5.94
4	Mean	12.4	18.3	2.58
100	SD	0.8	3.3	0.62
mg/kg/dose	N	2	2	2
	%Diff	-35.9	0.0	2.18

Table 7 Summary of Chemistry

Table 7 Summary of Chemistry

Key Page

Measurement Descriptions

Headings Used	Description
ALT	Alanine Aminotransferase
AST	Aspartate Aminotransferase
TP	Total Protein
ALB	Albumin
TBIL	Total Bilirubin
ALP	Alkaline Phosphatase
GGT	Gamma-Glutamyltransferase
sGLU	Glucose
UREA	Urea
CRE	Creatinine
Ca	Calcium
P	Inorganic Phosphorus
TCHO	Total Cholesterol
TG	Triglyceride
K	Potassium
Na	Sodium
Cl	Chloride
GLB	Globulin
A/G	A/G Ratio
CK	Creatine Kinase

Table 7 Summary of Chemistry-Pretest and Dosing Phase

Day(s) Relative to Start Date

Sex: Male		ALT (U/L) [a] -11	ALT (U/L) [a] -6	ALT (U/L) [a1] 53	ALT (U/L) [a] 87	ALT (U/L) [a] 123	ALT (U/L) [a] 179	AST (U/L) [a] -11	AST (U/L) [a1] -6	AST (U/L) [a] 53	AST (U/L) [a] 87	AST (U/L) [a] 123	AST (U/L) [a] 179
1	Mean	50	58	44	57	39	43	60	70	51	70	45	56
0	SD	9	13	7	16	6	9	14	24	10	33	6	11
mg/kg/dose	N	6	6	6	6	6	6	6	6	6	6	6	6
2	Mean	53	56	53	70	47	53	62	70	55	74	51	58
5	SD	19	17	21	26	12	13	15	15	15	30	18	21
mg/kg/dose	N	6	6	6	6	6	6	6	6	6	6	6	6
	%Diff	5	-4	21	24	21	22	3	1	7	6	12	3
3	Mean	51	48	47	58	42	46	49	50	42	62	44	46
25	SD	7	6	10	13	6	11	12	11	8	26	6	12
mg/kg/dose	N	6	6	6	6	6	6	6	6	6	6	6	6
	%Diff	2	-17	6	2	7	6	-18	-29	-18	-11	-4	-18
4	Mean	56	52	51	70	47	61	55	53	42	74	46	72
100	SD	18	11	11	25	9	20	8	10	9	40	13	54
mg/kg/dose	N	6	6	6	6	6	6	6	6	6	6	6	6
	%Diff	12	-10	17	23	21	41	-9	-24	-19	5	1	28

[a] - Anova & Dunnett
[a1] - Anova & Dunnett(Log)

Table 7 Summary of Chemistry-Pretest and Dosing Phase (Cont'd)

Day(s) Relative to Start Date

Sex: Male		TP (g/L) [a]	TP (g/L) [a]	TP (g/L) [a]	TP (g/L) [a]	TP (g/L) [a]	TP (g/L) [a]	ALB (g/L) [a]	ALB (g/L) [a]	ALB (g/L) [a]	ALB (g/L) [a]	ALB (g/L) [a]	ALB (g/L) [a]
		-11	-6	53	87	123	179	-11	-6	53	87	123	179
1	Mean	80.4	79.5	76.5	74.0	72.7	73.3	44.9	44.5	44.1	42.9	42.7	42.9
0	SD	5.6	2.3	6.2	2.8	1.8	2.8	3.0	2.2	2.9	2.1	2.1	1.8
mg/kg/dose	N	6	6	6	6	6	6	6	6	6	6	6	6
2	Mean	76.3	76.1	73.7	74.7	72.6	71.1	43.7	44.0	43.3	43.3	43.0	42.7
5	SD	3.4	2.5	4.9	3.9	3.9	2.8	4.4	3.4	1.7	1.8	1.8	1.3
mg/kg/dose	N	6	6	6	6	6	6	6	6	6	6	6	6
	%Diff	-5.1	-4.3	-3.7	0.9	-0.1	-3.1	-2.7	-1.1	-1.9	0.8	0.8	-0.4
3	Mean	77.7	77.1	74.4	72.6	71.5	72.2	45.1	44.6	44.5	43.0	41.4	43.1
25	SD	2.9	3.0	3.0	4.5	4.4	2.9	1.5	1.8	1.8	2.8	3.1	1.4
mg/kg/dose	N	6	6	6	6	6	6	6	6	6	6	6	6
	%Diff	-3.3	-3.1	-2.8	-1.9	-1.7	-1.5	0.4	0.4	0.9	0.1	-3.2	0.6
4	Mean	77.4	79.8	78.6	77.0	76.7	75.7	42.6	44.4	43.7	41.2	42.6	43.2
100	SD	3.4	5.3	2.7	3.6	3.7	3.1	3.3	3.6	2.4	2.2	3.2	1.8
mg/kg/dose	N	6	6	6	6	6	6	6	6	6	6	6	6
	%Diff	-3.7	0.3	2.7	4.1	5.5	3.3	-5.2	-0.1	-0.9	-4.0	-0.2	0.7

[a] - Anova & Dunnett

Table 7 Summary of Chemistry-Pretest and Dosing Phase (Cont'd)

Day(s) Relative to Start Date

Sex: Male		TBIL (µmol/L) [a] -11	TBIL (µmol/L) [a] -6	TBIL (µmol/L) [a] 53	TBIL (µmol/L) [a] 87	TBIL (µmol/L) [a] 123	TBIL (µmol/L) [a] 179	ALP (U/L) [a] -11	ALP (U/L) [a] -6	ALP (U/L) [a] 53	ALP (U/L) [a] 87	ALP (U/L) [a] 123	ALP (U/L) [a] 179
1	Mean	7.93	4.35	4.41	5.18	5.27	4.98	627	564	744	869	853	838
0	SD	2.02	0.74	1.16	1.61	2.41	1.17	217	153	183	216	138	148
mg/kg/dose	N	6	6	6	6	6	6	6	6	6	6	6	6
2	Mean	10.67	6.99**	5.86	6.08	7.48	7.01	588	532	640	729	703	686
5	SD	2.69	1.99	0.72	0.74	1.78	1.47	206	185	204	208	144	165
mg/kg/dose	N	6	6	6	6	6	6	6	6	6	6	6	6
	%Diff	34.52	60.70	32.84	17.35	41.86	40.65	-6	-6	-14	-16	-18	-18
3	Mean	7.51	4.99	4.51	6.42	4.91	4.64	562	518	663	723	646	607
25	SD	2.11	1.09	0.57	2.66	0.58	1.59	128	120	140	170	127	70
mg/kg/dose	N	6	6	6	6	6	6	6	6	6	6	6	6
	%Diff	-5.32	14.59	2.23	23.88	-6.80	-6.79	-10	-8	-11	-17	-24	-28
4	Mean	7.70	4.77	5.51	6.46	6.67	7.32	481	456	540	667	677	649
100	SD	1.15	0.66	1.60	1.93	2.03	2.54	126	95	151	198	214	172
mg/kg/dose	N	6	6	6	6	6	6	6	6	6	6	6	6
	%Diff	-2.94	9.61	24.94	24.75	26.46	46.87	-23	-19	-27	-23	-21	-23

[a] - Anova & Dunnett: ** = $p \leq 0.01$

Table 7 Summary of Chemistry-Pretest and Dosing Phase (Cont'd)

Day(s) Relative to Start Date

Sex: Male		GGT (U/L) [a] -11	GGT (U/L) [a] -6	GGT (U/L) [a] 53	GGT (U/L) [a] 87	GGT (U/L) [a] 123	GGT (U/L) [a] 179	sGLU (mmol/L) [a] -11	sGLU (mmol/L) [a] -6	sGLU (mmol/L) [a] 53	sGLU (mmol/L) [a] 87	sGLU (mmol/L) [a] 123	sGLU (mmol/L) [a] 179
1	Mean	75	70	88	94	105	96	3.08	4.74	3.72	3.33	3.35	3.43
0	SD	22	19	30	31	32	35	0.48	0.81	0.39	0.66	0.45	0.62
mg/kg/dose	N	6	6	6	6	6	6	6	6	6	6	6	6
2	Mean	76	70	80	87	96	87	2.81	4.07	3.59	3.40	3.20	3.34
5	SD	26	23	31	35	37	33	0.39	0.87	0.37	0.48	0.25	0.28
mg/kg/dose	N	6	6	6	6	6	6	6	6	6	6	6	6
	%Diff	2	0	-9	-7	-9	-10	-8.66	-14.03	-3.41	2.21	-4.33	-2.72
3	Mean	78	71	83	85	85	77	2.88	4.09	3.64	3.10	3.21	3.38
25	SD	34	30	31	34	28	21	0.35	0.64	0.36	0.65	0.44	0.68
mg/kg/dose	N	6	6	6	6	6	6	6	6	6	6	6	6
	%Diff	5	0	-5	-9	-19	-20	-6.44	-13.72	-2.11	-6.67	-4.03	-1.56
4	Mean	55	55	62	61	70	64	2.70	4.01	3.43	2.88	3.05	3.51
100	SD	12	11	18	16	15	14	0.32	0.61	0.35	0.18	0.20	0.32
mg/kg/dose	N	6	6	6	6	6	6	6	6	6	6	6	6
	%Diff	-26	-22	-29	-35	-34	-34	-12.45	-15.41	-7.89	-13.43	-8.76	2.48

[a] - Anova & Dunnett

Table 7 Summary of Chemistry-Pretest and Dosing Phase (Cont'd)

Day(s) Relative to Start Date

Sex: Male		UREA (mmol/L) [a] -11	UREA (mmol/L) [a1] -6	UREA (mmol/L) [a1] 53	UREA (mmol/L) [a] 87	UREA (mmol/L) [a1] 123	UREA (mmol/L) [a1] 179	CRE (µmol/L) [a1] -11	CRE (µmol/L) [a1] -6	CRE (µmol/L) [a1] 53	CRE (µmol/L) [a1] 87	CRE (µmol/L) [a1] 123	CRE (µmol/L) [a1] 179
1	Mean	7.42	5.96	6.65	6.91	7.32	6.99	62	57	60	60	61	62
0	SD	0.73	0.99	0.98	0.57	1.55	0.93	6	5	6	7	9	5
mg/kg/dose	N	6	6	6	6	6	6	6	6	6	6	6	6
2	Mean	8.03	6.14	7.49	7.35	8.19	8.01	66	62	64	64	69	68
5	SD	2.09	1.19	1.48	1.13	0.96	1.40	16	15	16	12	16	15
mg/kg/dose	N	6	6	6	6	6	6	6	6	6	6	6	6
	%Diff	8.32	3.11	12.69	6.39	11.93	14.59	6	8	6	6	13	9
3	Mean	6.96	5.45	6.19	6.22	6.75	6.06	63	59	61	59	64	64
25	SD	0.52	0.87	0.95	1.01	0.64	1.14	8	7	6	5	6	8
mg/kg/dose	N	6	6	6	6	6	6	6	6	6	6	6	6
	%Diff	-6.20	-8.54	-6.90	-9.96	-7.79	-13.42	1	4	2	-2	6	3
4	Mean	7.25	5.15	7.05	7.16	7.95	7.54	61	59	61	61	61	61
100	SD	0.85	0.67	0.95	0.31	1.03	1.21	9	9	10	12	11	12
mg/kg/dose	N	6	6	6	6	6	6	6	6	6	6	6	6
	%Diff	-2.25	-13.49	6.07	3.62	8.56	7.77	-1	3	2	1	1	-2

[a] - Kruskal-Wallis & Dunnett on Ranks
[a1] - Anova & Dunnett

Table 7 Summary of Chemistry-Pretest and Dosing Phase (Cont'd)

Day(s) Relative to Start Date

Sex: Male		Ca (mmol/L) [a] -11	Ca (mmol/L) [a1] -6	Ca (mmol/L) [a] 53	Ca (mmol/L) [a1] 87	Ca (mmol/L) [a1] 123	Ca (mmol/L) [a] 179	P (mmol/L) [a1] -11	P (mmol/L) [a1] -6	P (mmol/L) [a1] 53	P (mmol/L) [a1] 87	P (mmol/L) [a1] 123	P (mmol/L) [a1] 179
1	Mean	2.43	2.57	2.64	2.51	2.46	2.43	2.11	1.89	2.24	2.24	2.19	2.23
0	SD	0.06	0.11	0.15	0.10	0.07	0.06	0.30	0.21	0.36	0.31	0.40	0.13
mg/kg/dose	N	6	6	6	6	6	6	6	6	6	6	6	6
2	Mean	2.41	2.64	2.60	2.55	2.43	2.40	2.15	2.15	2.23	2.31	2.23	2.26
5	SD	0.10	0.13	0.09	0.10	0.10	0.11	0.19	0.24	0.18	0.35	0.20	0.21
mg/kg/dose	N	6	6	6	6	6	6	6	6	6	6	6	6
	%Diff	-0.69	2.66	-1.64	1.80	-1.42	-1.58	2.22	13.73	-0.37	3.20	1.98	1.57
3	Mean	2.41	2.58	2.62	2.49	2.46	2.45	2.23	2.00	2.12	2.25	2.13	2.25
25	SD	0.06	0.05	0.05	0.07	0.08	0.05	0.49	0.37	0.44	0.34	0.35	0.34
mg/kg/dose	N	6	6	6	6	6	6	6	6	6	6	6	6
	%Diff	-0.48	0.13	-0.63	-0.60	-0.07	0.48	6.01	5.37	-5.36	0.30	-2.52	0.97
4	Mean	2.35	2.60	2.53	2.42	2.37	2.36	2.10	1.99	2.17	2.29	2.25	2.34
100	SD	0.13	0.13	0.10	0.06	0.13	0.03	0.32	0.31	0.45	0.43	0.30	0.29
mg/kg/dose	N	6	6	6	6	6	6	6	6	6	6	6	6
	%Diff	-3.23	1.23	-4.17	-3.39	-3.65	-3.22	-0.24	5.02	-3.05	2.16	3.05	5.32

[a] - Kruskal-Wallis & Dunnett on Ranks
[a1] - Anova & Dunnett

Table 7 Summary of Chemistry-Pretest and Dosing Phase (Cont'd)

Day(s) Relative to Start Date

Sex: Male		TCHO (mmol/L) [a] -11	TCHO (mmol/L) [a] -6	TCHO (mmol/L) [a] 53	TCHO (mmol/L) [a] 87	TCHO (mmol/L) [a] 123	TCHO (mmol/L) [a] 179	TG (mmol/L) [a] -11	TG (mmol/L) [a] -6	TG (mmol/L) [a1] 53	TG (mmol/L) [a] 87	TG (mmol/L) [a] 123	TG (mmol/L) [a] 179
1	Mean	3.41	2.98	3.60	3.41	3.55	3.41	0.45	0.30	0.46	0.48	0.46	0.41
0	SD	0.68	0.39	0.87	0.77	0.66	0.63	0.09	0.05	0.13	0.08	0.15	0.10
mg/kg/dose	N	6	6	6	6	6	6	6	6	6	6	6	6
2	Mean	3.87	3.84	3.97	3.98	4.07	3.76	0.61	0.36	0.54	0.47	0.62*	0.47
5	SD	0.88	0.70	0.62	0.50	0.78	0.90	0.21	0.08	0.12	0.12	0.09	0.08
mg/kg/dose	N	6	6	6	6	6	6	6	6	6	6	6	6
	%Diff	13.38	28.66	10.43	16.66	14.50	10.31	37.31	17.58	18.98	-1.39	34.55	14.98
3	Mean	3.77	3.66	3.79	3.49	3.36	3.39	0.52	0.31	0.45	0.46	0.56	0.45
25	SD	0.71	0.79	0.47	0.57	0.51	0.35	0.13	0.06	0.05	0.07	0.08	0.12
mg/kg/dose	N	6	6	6	6	6	6	6	6	6	6	6	6
	%Diff	10.45	22.63	5.38	2.39	-5.54	-0.68	16.42	1.10	-0.73	-4.86	22.55	8.50
4	Mean	3.45	3.26	3.60	3.38	3.34	3.13	0.54	0.28	0.49	0.42	0.46	0.42
100	SD	0.90	0.74	0.49	0.55	0.40	0.34	0.09	0.03	0.03	0.08	0.06	0.06
mg/kg/dose	N	6	6	6	6	6	6	6	6	6	6	6	6
	%Diff	1.07	9.22	0.14	-0.83	-5.96	-8.11	20.90	-8.79	7.30	-12.85	-0.73	2.02

[a] - Anova & Dunnett: * = p ≤ 0.05
[a1] - Kruskal-Wallis & Dunnett on Ranks

Table 7 Summary of Chemistry-Pretest and Dosing Phase (Cont'd)

Day(s) Relative to Start Date

Sex: Male		K (mmol/L) [a] -11	K (mmol/L) [a] -6	K (mmol/L) [a] 53	K (mmol/L) [a] 87	K (mmol/L) [a] 123	K (mmol/L) [a] 179	Na (mmol/L) [a1] -11	Na (mmol/L) [a] -6	Na (mmol/L) [a] 53	Na (mmol/L) [a1] 87	Na (mmol/L) [a] 123	Na (mmol/L) [a] 179
1	Mean	4.3	4.7	4.9	4.6	4.9	4.6	149	151	146	147	145	145
0	SD	0.4	0.7	0.8	0.6	0.7	0.8	3	5	4	3	3	2
mg/kg/dose	N	6	6	6	6	6	6	6	6	6	6	6	6
2	Mean	4.4	5.0	4.8	5.2	4.4	4.4	148	150	145	148	145	144
5	SD	0.4	0.4	0.5	0.7	0.4	0.4	1	2	3	1	2	1
mg/kg/dose	N	6	6	6	6	6	6	6	6	6	6	6	6
	%Diff	2.3	5.3	-0.3	12.8	-11.2	-5.1	-1	-1	-1	0	0	-1
3	Mean	4.5	4.7	4.9	4.8	4.6	4.6	148	151	146	147	145	145
25	SD	0.5	0.5	0.5	0.8	0.5	0.7	1	2	3	1	3	1
mg/kg/dose	N	6	6	6	6	6	6	6	6	6	6	6	6
	%Diff	4.7	-0.4	0.7	4.7	-5.4	0.0	-1	0	0	0	0	0
4	Mean	4.4	5.1	4.7	5.1	4.7	4.8	146 *	151	146	148	144	144
100	SD	0.7	0.8	0.6	1.2	0.4	1.0	1	4	1	2	1	2
mg/kg/dose	N	6	6	6	6	6	6	6	6	6	6	6	6
	%Diff	2.7	8.1	-3.4	10.9	-4.8	5.5	-2	0	0	0	0	-1

[a] - Anova & Dunnett
[a1] - Kruskal-Wallis & Dunnett on Ranks: * = p ≤ 0.05

Table 7 Summary of Chemistry-Pretest and Dosing Phase (Cont'd)

Day(s) Relative to Start Date

Sex: Male		Cl (mmol/L) [a] -11	Cl (mmol/L) [a] -6	Cl (mmol/L) [a] 53	Cl (mmol/L) [a] 87	Cl (mmol/L) [a] 123	Cl (mmol/L) [a] 179	GLB (g/L) [a] -11	GLB (g/L) [a] -6	GLB (g/L) [a] 53	GLB (g/L) [a] 87	GLB (g/L) [a] 123	GLB (g/L) [a] 179
1	Mean	104	106	103	103	104	104	35.5	35.1	32.4	31.1	30.0	30.4
0	SD	1	3	2	1	2	3	3.1	2.6	3.8	1.7	1.7	1.7
mg/kg/dose	N	6	6	6	6	6	6	6	6	6	6	6	6
2	Mean	104	106	102	103	101 *	103	32.6	32.2	30.5	31.5	29.6	28.4
5	SD	1	1	2	2	2	1	3.4	2.7	4.0	3.7	3.6	2.7
mg/kg/dose	N	6	6	6	6	6	6	6	6	6	6	6	6
	%Diff	0	0	-1	0	-3	-1	-8.1	-8.2	-6.1	1.2	-1.4	-6.8
3	Mean	104	107	104	104	102	103	32.6	32.4	29.9	29.7	30.1	29.1
25	SD	2	2	1	2	2	1	2.2	2.3	2.0	2.0	3.9	1.8
mg/kg/dose	N	6	6	6	6	6	6	6	6	6	6	6	6
	%Diff	0	0	1	1	-2	0	-8.0	-7.5	-7.8	-4.6	0.5	-4.4
4	Mean	103	106	101	105	101 *	103	34.8	35.4	34.9	35.8*	34.1	32.5
100	SD	1	3	1	2	1	2	2.6	3.1	1.4	4.0	2.1	1.5
mg/kg/dose	N	6	6	6	6	6	6	6	6	6	6	6	6
	%Diff	-1	-1	-2	1	-3	-1	-1.7	0.9	7.5	15.2	13.6	6.8

[a] - Anova & Dunnett: * = $p \leq 0.05$

Table 7 Summary of Chemistry-Pretest and Dosing Phase (Cont'd)

Day(s) Relative to Start Date

Sex: Male		A/G [a]	A/G [a]	A/G [a]	A/G [a]	A/G [a]	A/G [a1]	CK (U/L) [a]	CK (U/L) [a2]	CK (U/L) [a2]	CK (U/L) [a]	CK (U/L) [a2]	CK (U/L) [a2]
		-11	-6	53	87	123	179	-11	-6	53	87	123	179
1	Mean	1.27	1.28	1.37	1.38	1.43	1.41	499	386	378	992	223	653
0	SD	0.08	0.13	0.11	0.10	0.14	0.09	475	294	173	1541	52	454
mg/kg/dose	N	6	6	6	6	6	6	6	6	6	6	6	6
2	Mean	1.36	1.38	1.44	1.39	1.48	1.52	481	302	509	1240	369	308
5	SD	0.23	0.20	0.18	0.19	0.19	0.17	478	66	648	1677	190	198
mg/kg/dose	N	6	6	6	6	6	6	6	6	6	6	6	6
	%Diff	7.11	8.14	5.25	0.68	3.13	7.55	-4	-22	35	25	65	-53
3	Mean	1.39	1.38	1.49	1.45	1.39	1.49*	288	200	218	844	249	223
25	SD	0.10	0.11	0.11	0.06	0.20	0.07	202	52	73	1278	154	86
mg/kg/dose	N	6	6	6	6	6	6	6	6	6	6	6	6
	%Diff	9.12	8.33	9.18	4.75	-2.65	5.18	-42	-48	-42	-15	12	-66
4	Mean	1.23	1.26	1.26	1.16*	1.26	1.33	349	268	253	1830	560	1035
100	SD	0.16	0.14	0.09	0.16	0.13	0.05	160	121	40	3490	637	1903
mg/kg/dose	N	6	6	6	6	6	6	6	6	6	6	6	6
	%Diff	-3.16	-1.06	-8.18	-15.81	-12.23	-5.81	-30	-31	-33	84	151	58

[a] - Anova & Dunnett: * = p ≤ 0.05
[a1] - Kruskal-Wallis & Dunnett on Ranks: * = p ≤ 0.05
[a2] - Anova & Dunnett(Log)

Table 7 Summary of Chemistry-Pretest and Dosing Phase (Cont'd)

Day(s) Relative to Start Date

Sex: Female		ALT (U/L) [a] -12	ALT (U/L) [a] -7	ALT (U/L) [a] 52	ALT (U/L) [a] 86	ALT (U/L) [a1] 122	ALT (U/L) [a] 178	AST (U/L) [a] -12	AST (U/L) [a] -7	AST (U/L) [a] 52	AST (U/L) [a] 86	AST (U/L) [a] 122	AST (U/L) [a1] 178
1	Mean	54	57	52	57	50	48	57	61	50	90	51	56
0	SD	18	15	6	9	15	8	15	21	9	18	13	19
mg/kg/dose	N	6	6	6	6	6	6	6	6	6	6	6	6
2	Mean	50	57	49	64	75	48	52	57	44	92	55	65
5	SD	11	14	10	17	72	11	16	21	8	37	31	33
mg/kg/dose	N	6	6	6	6	6	6	6	6	6	6	6	6
	%Diff	-7	0	-5	12	51	1	-9	-6	-13	2	7	15
3	Mean	48	57	43	50	37	45	62	66	58	75	41	50
25	SD	20	23	10	17	12	13	18	22	32	31	7	17
mg/kg/dose	N	6	6	6	6	6	6	6	6	6	6	6	6
	%Diff	-10	0	-17	-12	-26	-5	8	9	15	-17	-19	-11
4	Mean	58	54	60	60	57	61	45	48	41	59	38	48
100	SD	16	9	15	14	19	19	11	21	12	12	9	12
mg/kg/dose	N	6	6	6	6	6	6	6	6	6	6	6	6
	%Diff	9	-4	16	5	14	28	-22	-20	-18	-34	-25	-15

[a] - Anova & Dunnett
[a1] - Anova & Dunnett(Log)

Table 7 Summary of Chemistry-Pretest and Dosing Phase (Cont'd)

Day(s) Relative to Start Date

Sex: Female		TP (g/L) [a] -12	TP (g/L) [a] -7	TP (g/L) [a] 52	TP (g/L) [a] 86	TP (g/L) [a] 122	TP (g/L) [a] 178	ALB (g/L) [a] -12	ALB (g/L) [a] -7	ALB (g/L) [a] 52	ALB (g/L) [a] 86	ALB (g/L) [a] 122	ALB (g/L) [a] 178
1	Mean	80.6	80.5	78.5	76.8	76.5	75.9	43.8	44.5	44.4	43.3	43.0	43.2
0	SD	2.7	2.5	4.8	3.9	2.4	5.8	2.5	2.5	2.3	2.0	1.9	2.1
mg/kg/dose	N	6	6	6	6	6	6	6	6	6	6	6	6
2	Mean	80.9	80.8	77.9	76.0	76.7	75.2	41.7	42.8	42.8	42.0	40.0	42.1
5	SD	4.1	1.8	5.0	1.8	5.5	3.1	2.2	1.7	2.7	1.7	4.6	1.4
mg/kg/dose	N	6	6	6	6	6	6	6	6	6	6	6	6
	%Diff	0.4	0.4	-0.7	-1.0	0.3	-0.9	-4.8	-3.9	-3.7	-3.1	-6.8	-2.5
3	Mean	79.3	79.4	77.1	73.9	76.4	75.1	43.0	44.1	43.4	41.2	42.2	42.3
25	SD	3.5	3.7	2.3	3.7	2.3	3.4	2.2	2.7	2.3	1.5	2.1	1.4
mg/kg/dose	N	6	6	6	6	6	6	6	6	6	6	6	6
	%Diff	-1.6	-1.3	-1.7	-3.8	-0.2	-1.1	-1.9	-0.9	-2.2	-4.9	-1.7	-2.1
4	Mean	74.6*	76.2	75.4	75.1	76.8	74.8	42.1	42.9	42.7	41.5	42.9	42.9
100	SD	4.3	4.3	3.5	3.7	3.3	6.0	2.5	2.2	1.5	1.1	1.8	2.1
mg/kg/dose	N	6	6	6	6	6	6	6	6	6	6	6	6
	%Diff	-7.4	-5.2	-3.9	-2.1	0.4	-1.5	-4.0	-3.6	-3.8	-4.1	-0.3	-0.8

[a] - Anova & Dunnett: * = $p \leq 0.05$

Table 7 Summary of Chemistry-Pretest and Dosing Phase (Cont'd)

Day(s) Relative to Start Date

Sex: Female		TBIL (µmol/L) [a] -12	TBIL (µmol/L) [a] -7	TBIL (µmol/L) [a] 52	TBIL (µmol/L) [a] 86	TBIL (µmol/L) [a] 122	TBIL (µmol/L) [a] 178	ALP (U/L) [a] -12	ALP (U/L) [a] -7	ALP (U/L) [a] 52	ALP (U/L) [a] 86	ALP (U/L) [a] 122	ALP (U/L) [a] 178
1	Mean	4.61	5.07	4.72	5.01	4.61	5.08	423	436	450	488	434	438
0	SD	1.50	1.85	2.18	2.19	1.93	2.49	122	129	109	116	126	139
mg/kg/dose	N	6	6	6	6	6	6	6	6	6	6	6	6
2	Mean	4.10	4.43	4.60	4.93	6.70	4.70	437	436	538	611	550	558
5	SD	0.46	0.56	1.28	1.28	4.55	1.10	61	47	64	50	44	37
mg/kg/dose	N	6	6	6	6	6	6	6	6	6	6	6	6
	%Diff	-11.06	-12.53	-2.61	-1.63	45.46	-7.49	3	0	20	25	27	27
3	Mean	4.97	5.33	4.26	5.55	5.28	4.51	474	468	517	553	488	494
25	SD	1.07	1.02	1.24	1.75	2.17	1.70	131	143	151	147	143	137
mg/kg/dose	N	6	6	6	6	6	6	6	6	6	6	6	6
	%Diff	7.84	5.16	-9.81	10.78	14.73	-11.07	12	7	15	13	13	13
4	Mean	4.16	4.89	4.55	4.86	5.16	5.12	454	461	419	467	455	446
100	SD	2.09	1.58	1.74	1.59	1.82	1.47	121	118	82	102	121	108
mg/kg/dose	N	6	6	6	6	6	6	6	6	6	6	6	6
	%Diff	-9.79	-3.62	-3.60	-2.93	12.12	0.85	7	6	-7	-4	5	2

[a] - Anova & Dunnett

Table 7 Summary of Chemistry-Pretest and Dosing Phase (Cont'd)

Day(s) Relative to Start Date

Sex: Female		GGT (U/L) [a] -12	GGT (U/L) [a] -7	GGT (U/L) [a1] 52	GGT (U/L) [a] 86	GGT (U/L) [a1] 122	GGT (U/L) [a1] 178	sGLU (mmol/L) [a2] -12	sGLU (mmol/L) [a2] -7	sGLU (mmol/L) [a2] 52	sGLU (mmol/L) [a2] 86	sGLU (mmol/L) [a2] 122	sGLU (mmol/L) [a2] 178
1	Mean	52	52	51	52	53	51	3.46	3.76	3.84	3.23	3.96	3.76
0	SD	5	5	7	7	6	7	0.97	0.49	0.95	0.67	0.79	0.94
mg/kg/dose	N	6	6	6	6	6	6	6	6	6	6	6	6
2	Mean	74	74	80	78	79	76	3.34	3.68	3.39	2.91	3.39	3.04
5	SD	31	31	35	31	22	27	0.34	0.44	0.45	0.31	0.42	0.64
mg/kg/dose	N	6	6	6	6	6	6	6	6	6	6	6	6
	%Diff	43	43	57	51	49	50	-3.33	-2.17	-11.60	-9.87	-14.52	-19.31
3	Mean	44	45	45	44	46	46	2.89	3.69	3.47	2.66	3.23	3.12
25	SD	9	11	11	9	10	10	0.76	0.58	0.26	0.21	0.46	0.54
mg/kg/dose	N	6	6	6	6	6	6	6	6	6	6	6	6
	%Diff	-14	-13	-11	-15	-13	-10	-16.40	-1.82	-9.51	-17.56	-18.52	-17.05
4	Mean	69	65	64	64	67	62	3.51	3.31	3.55	3.34	3.36	3.31
100	SD	29	28	23	23	21	21	0.98	0.66	0.73	1.06	0.67	1.17
mg/kg/dose	N	6	6	6	6	6	6	6	6	6	6	6	6
	%Diff	33	26	25	24	26	22	1.64	-11.96	-7.47	3.56	-15.11	-11.96

[a] - Kruskal-Wallis & Dunnett on Ranks
[a1] - Anova & Dunnett(Log)
[a2] - Anova & Dunnett

Table 7 Summary of Chemistry-Pretest and Dosing Phase (Cont'd)

Day(s) Relative to Start Date

Sex: Female		UREA (mmol/L) [a] -12	UREA (mmol/L) [a] -7	UREA (mmol/L) [a] 52	UREA (mmol/L) [a] 86	UREA (mmol/L) [a] 122	UREA (mmol/L) [a] 178	CRE (µmol/L) [a] -12	CRE (µmol/L) [a] -7	CRE (µmol/L) [a] 52	CRE (µmol/L) [a] 86	CRE (µmol/L) [a] 122	CRE (µmol/L) [a] 178
1	Mean	6.18	5.65	6.32	7.09	6.85	6.90	54	58	58	59	61	60
0	SD	1.06	0.79	0.46	1.33	0.87	1.64	6	6	10	9	6	8
mg/kg/dose	N	6	6	6	6	6	6	6	6	6	6	6	6
2	Mean	5.90	5.10	6.64	7.01	7.29	7.17	56	59	62	63	69	66
5	SD	1.21	1.13	0.57	1.78	0.57	1.03	7	8	7	5	10	12
mg/kg/dose	N	6	6	6	6	6	6	6	6	6	6	6	6
	%Diff	-4.56	-9.68	5.14	-1.06	6.52	3.99	5	1	7	7	12	10
3	Mean	6.68	5.28	6.28	6.65	6.47	6.62	56	61	60	59	60	59
25	SD	1.65	0.74	0.75	1.00	0.65	1.13	5	4	8	7	6	5
mg/kg/dose	N	6	6	6	6	6	6	6	6	6	6	6	6
	%Diff	8.09	-6.61	-0.55	-6.19	-5.55	-4.04	5	5	3	0	-2	-1
4	Mean	6.08	5.64	6.63	6.56	7.81	7.30	54	58	59	59	65	62
100	SD	2.08	1.42	1.12	1.50	1.74	1.37	7	8	7	8	11	10
mg/kg/dose	N	6	6	6	6	6	6	6	6	6	6	6	6
	%Diff	-1.62	-0.21	4.93	-7.48	14.00	5.85	0	-1	2	0	5	4

[a] - Anova & Dunnett

Table 7 Summary of Chemistry-Pretest and Dosing Phase (Cont'd)

Day(s) Relative to Start Date

Sex: Female		Ca (mmol/L) [a] -12	Ca (mmol/L) [a] -7	Ca (mmol/L) [a] 52	Ca (mmol/L) [a] 86	Ca (mmol/L) [a] 122	Ca (mmol/L) [a] 178	P (mmol/L) [a] -12	P (mmol/L) [a] -7	P (mmol/L) [a] 52	P (mmol/L) [a1] 86	P (mmol/L) [a1] 122	P (mmol/L) [a] 178
1	Mean	2.56	2.75	2.65	2.52	2.53	2.51	1.83	1.95	1.93	1.82	1.94	1.91
0	SD	0.10	0.30	0.08	0.10	0.10	0.16	0.26	0.17	0.10	0.11	0.07	0.18
mg/kg/dose	N	6	6	6	6	6	6	6	6	6	6	6	6
2	Mean	2.51	2.62	2.55	2.48	2.44	2.44	1.73	1.82	1.96	1.68	1.85	2.03
5	SD	0.09	0.06	0.11	0.06	0.11	0.06	0.23	0.12	0.18	0.20	0.26	0.24
mg/kg/dose	N	6	6	6	6	6	6	6	6	6	6	6	6
	%Diff	-2.02	-4.79	-3.84	-1.59	-3.50	-2.92	-5.29	-6.68	1.38	-7.61	-4.72	6.27
3	Mean	2.49	2.77	2.59	2.45	2.45	2.43	1.79	2.02	2.03	1.82	1.97	2.04
25	SD	0.08	0.26	0.13	0.08	0.12	0.10	0.18	0.09	0.32	0.17	0.15	0.11
mg/kg/dose	N	6	6	6	6	6	6	6	6	6	6	6	6
	%Diff	-2.87	0.85	-2.20	-2.91	-3.23	-3.12	-2.28	3.85	5.35	0.00	1.54	6.45
4	Mean	2.48	2.62	2.57	2.48	2.47	2.44	1.71	1.90	1.79	1.75	1.90	1.98
100	SD	0.13	0.12	0.09	0.13	0.13	0.20	0.24	0.23	0.22	0.31	0.36	0.30
mg/kg/dose	N	6	6	6	6	6	6	6	6	6	6	6	6
	%Diff	-2.93	-4.85	-3.14	-1.59	-2.18	-2.92	-6.75	-2.57	-7.25	-3.49	-2.49	3.66

[a] - Anova & Dunnett
[a1] - Kruskal-Wallis & Dunnett on Ranks

Table 7 Summary of Chemistry-Pretest and Dosing Phase (Cont'd)

Day(s) Relative to Start Date

Sex: Female		TCHO (mmol/L) [a] -12	TCHO (mmol/L) [a] -7	TCHO (mmol/L) [a] 52	TCHO (mmol/L) [a] 86	TCHO (mmol/L) [a] 122	TCHO (mmol/L) [a] 178	TG (mmol/L) [a] -12	TG (mmol/L) [a] -7	TG (mmol/L) [a] 52	TG (mmol/L) [a] 86	TG (mmol/L) [a] 122	TG (mmol/L) [a] 178
1	Mean	3.66	3.43	4.01	3.81	4.12	3.89	0.43	0.39	0.59	0.62	0.62	0.52
0	SD	0.92	0.80	0.65	0.83	0.70	0.78	0.06	0.08	0.15	0.16	0.12	0.15
mg/kg/dose	N	6	6	6	6	6	6	6	6	6	6	6	6
2	Mean	3.90	3.49	3.86	3.60	4.48	3.69	0.39	0.32	0.46	0.45	0.63	0.40
5	SD	0.54	0.82	0.73	0.69	1.41	0.64	0.10	0.05	0.14	0.08	0.37	0.15
mg/kg/dose	N	6	6	6	6	6	6	6	6	6	6	6	6
	%Diff	6.70	1.60	-3.66	-5.56	8.86	-5.31	-9.30	-19.49	-22.82	-26.83	0.80	-23.00
3	Mean	3.38	3.32	3.59	3.52	3.85	3.62	0.41	0.34	0.46	0.40*	0.45	0.37
25	SD	0.97	0.92	0.99	1.09	1.22	1.17	0.15	0.19	0.13	0.09	0.17	0.11
mg/kg/dose	N	6	6	6	6	6	6	6	6	6	6	6	6
	%Diff	-7.66	-3.30	-10.56	-7.57	-6.52	-6.98	-4.65	-12.71	-23.10	-34.96	-27.35	-29.71
4	Mean	3.42	3.31	3.57	3.38	3.59	3.41	0.38	0.39	0.48	0.42*	0.52	0.49
100	SD	0.68	0.85	0.59	0.55	0.51	0.63	0.11	0.09	0.12	0.15	0.14	0.12
mg/kg/dose	N	6	6	6	6	6	6	6	6	6	6	6	6
	%Diff	-6.34	-3.69	-10.93	-11.24	-12.95	-12.37	-10.85	-1.69	-18.59	-31.71	-16.35	-6.71

[a] - Anova & Dunnett: * = $p \leq 0.05$

Table 7 Summary of Chemistry-Pretest and Dosing Phase (Cont'd)

Day(s) Relative to Start Date

Sex: Female		K (mmol/L) [a] -12	K (mmol/L) [a1] -7	K (mmol/L) [a] 52	K (mmol/L) [a] 86	K (mmol/L) [a] 122	K (mmol/L) [a] 178	Na (mmol/L) [a] -12	Na (mmol/L) [a] -7	Na (mmol/L) [a] 52	Na (mmol/L) [a] 86	Na (mmol/L) [a] 122	Na (mmol/L) [a] 178
1	Mean	5.0	5.0	5.1	4.9	4.9	5.0	149	152	147	149	145	147
0	SD	0.7	0.3	0.5	0.5	0.3	0.8	3	1	4	1	1	4
mg/kg/dose	N	6	6	6	6	6	6	6	6	6	6	6	6
2	Mean	4.6	4.7	4.7	4.7	4.4	4.7	149	152	148	149	146	146
5	SD	0.5	0.2	0.5	0.4	0.6	0.4	2	2	4	1	2	2
mg/kg/dose	N	6	6	6	6	6	6	6	6	6	6	6	6
	%Diff	-9.3	-6.0	-8.8	-4.1	-10.2	-6.0	0	0	0	0	1	-1
3	Mean	4.3	5.3	5.1	4.6	4.9	4.6	149	154	147	149	146	146
25	SD	0.3	0.8	0.4	0.5	0.4	0.4	1	3	4	3	3	1
mg/kg/dose	N	6	6	6	6	6	6	6	6	6	6	6	6
	%Diff	-14.0	5.6	-1.0	-5.4	-0.3	-7.3	0	1	0	0	1	0
4	Mean	4.8	4.8	4.7	4.7	4.5	4.7	151	152	147	147	147	147
100	SD	0.4	0.6	0.3	0.6	0.4	0.6	3	3	3	4	3	5
mg/kg/dose	N	6	6	6	6	6	6	6	6	6	6	6	6
	%Diff	-5.3	-4.0	-7.5	-5.1	-8.5	-7.0	1	0	0	-1	1	0

[a] - Anova & Dunnett
[a1] - Kruskal-Wallis & Dunnett on Ranks

Table 7 Summary of Chemistry-Pretest and Dosing Phase (Cont'd)

Day(s) Relative to Start Date

Sex: Female		Cl (mmol/L) [a] -12	Cl (mmol/L) [a] -7	Cl (mmol/L) [a] 52	Cl (mmol/L) [a] 86	Cl (mmol/L) [a] 122	Cl (mmol/L) [a] 178	GLB (g/L) [a] -12	GLB (g/L) [a] -7	GLB (g/L) [a] 52	GLB (g/L) [a] 86	GLB (g/L) [a] 122	GLB (g/L) [a] 178
1	Mean	104	107	104	106	102	104	36.8	36.0	34.1	33.5	33.6	32.7
0	SD	1	2	3	2	3	2	2.8	2.7	4.3	3.9	3.9	5.7
mg/kg/dose	N	6	6	6	6	6	6	6	6	6	6	6	6
2	Mean	104	107	104	106	102	103	39.2	38.0	35.2	34.1	36.7	33.1
5	SD	3	2	1	2	2	0	3.4	2.3	2.6	1.2	1.3	2.0
mg/kg/dose	N	6	6	6	6	6	6	6	6	6	6	6	6
	%Diff	1	0	0	0	0	-1	6.6	5.7	3.2	1.8	9.3	1.2
3	Mean	104	108	104	107	103	104	36.3	35.3	33.7	32.7	34.2	32.8
25	SD	2	2	2	2	2	2	3.4	2.7	2.4	4.1	3.0	3.4
mg/kg/dose	N	6	6	6	6	6	6	6	6	6	6	6	6
	%Diff	0	1	0	1	0	0	-1.2	-1.8	-1.2	-2.2	1.9	0.2
4	Mean	106	108	104	105	103	104	32.5	33.4	32.7	33.6	34.0	31.9
100	SD	2	2	2	1	2	2	2.8	2.8	2.6	2.6	2.0	4.4
mg/kg/dose	N	6	6	6	6	6	6	6	6	6	6	6	6
	%Diff	3	0	0	-1	0	0	-11.5	-7.2	-4.1	0.3	1.2	-2.5

[a] - Anova & Dunnett

Table 7 Summary of Chemistry-Pretest and Dosing Phase (Cont'd)

Day(s) Relative to Start Date

Sex: Female		A/G [a]	A/G [a]	A/G [a]	A/G [a1]	A/G [a]	A/G [a1]	CK (U/L) [a]	CK (U/L) [a]	CK (U/L) [a2]	CK (U/L) [a]	CK (U/L) [a]	CK (U/L) [a1]
		-12	-7	52	86	122	178	-12	-7	52	86	122	178
1	Mean	1.20	1.24	1.32	1.31	1.30	1.35	318	259	382	568	401	215
0	SD	0.13	0.14	0.17	0.16	0.19	0.22	135	100	311	187	445	51
mg/kg/dose	N	6	6	6	6	6	6	6	6	6	6	6	6
2	Mean	1.07	1.13	1.22	1.23	1.09	1.28	403	362	263	973	406	1602
5	SD	0.10	0.10	0.06	0.07	0.11	0.06	230	174	83	486	159	2067
mg/kg/dose	N	6	6	6	6	6	6	6	6	6	6	6	6
	%Diff	-10.77	-9.31	-7.72	-5.80	-16.05	-5.71	27	40	-31	71	1	645
3	Mean	1.20	1.25	1.30	1.28	1.25	1.30	301	418	1129	566	260	301
25	SD	0.15	0.13	0.14	0.18	0.15	0.15	138	271	2081	403	116	206
mg/kg/dose	N	6	6	6	6	6	6	6	6	6	6	6	6
	%Diff	-0.36	0.73	-1.71	-2.45	-4.08	-3.59	-5	62	195	0	-35	40
4	Mean	1.30	1.29	1.31	1.24	1.26	1.36	365	410	467	648	291	920
100	SD	0.10	0.09	0.10	0.06	0.06	0.15	80	250	366	200	100	1026
mg/kg/dose	N	6	6	6	6	6	6	6	6	6	6	6	6
	%Diff	8.26	3.62	-0.47	-5.22	-2.62	0.59	15	58	22	14	-28	328

[a] - Anova & Dunnett
[a1] - Kruskal-Wallis & Dunnett on Ranks
[a2] - Anova & Dunnett(Log)

Table 7 Summary of Chemistry-Recovery Phase

Day: 209 Relative to Start Date

Sex: Female		ALT (U/L)	AST (U/L)	TP (g/L)	ALB (g/L)	TBIL (µmol/L)	ALP (U/L)	GGT (U/L)	sGLU (mmol/L)	UREA (mmol/L)	CRE (µmol/L)	Ca (mmol/L)	P (mmol/L)
1	Mean	44	38	79.9	47.2	4.31	473	54	4.08	8.34	61	2.72	1.93
0	SD	6	8	1.6	4.3	0.76	21	8	0.39	2.97	6	0.24	0.26
mg/kg/dose	N	2	2	2	2	2	2	2	2	2	2	2	2
2	Mean	41	36	75.5	44.2	4.84	548	90	3.00	6.74	59	2.56	1.74
5	SD	17	11	1.5	1.1	1.39	51	40	0.25	1.15	6	0.04	0.08
mg/kg/dose	N	2	2	2	2	2	2	2	2	2	2	2	2
	%Diff	-6	-4	-5.6	-6.3	12.43	16	68	-26.50	-19.24	-4	-5.88	-9.61
3	Mean	51	40	74.8	43.1	5.48	572	42	2.99	6.80	62	2.54	1.89
25	SD	1	8	3.0	0.3	0.30	27	17	0.21	0.06	11	0.09	0.12
mg/kg/dose	N	2	2	2	2	2	2	2	2	2	2	2	2
	%Diff	16	5	-6.4	-8.6	27.18	21	-21	-26.75	-18.53	1	-6.80	-2.08
4	Mean	58	34	71.7	43.2	5.49	485	50	2.58	8.84	55	2.47	2.18
100	SD	8	5	3.5	1.5	0.47	59	24	0.62	0.64	6	0.22	0.12
mg/kg/dose	N	2	2	2	2	2	2	2	2	2	2	2	2
	%Diff	32	-11	-10.3	-8.5	27.53	3	-7	-36.69	5.94	-10	-9.38	12.99

Table 7 Summary of Chemistry-Recovery Phase (Cont'd)

Day: 209 Relative to Start Date

Sex: Female		TCHO (mmol/L)	TG (mmol/L)	K (mmol/L)	Na (mmol/L)	Cl (mmol/L)	GLB (g/L)	A/G	CK (U/L)
1	Mean	4.19	0.55	5.4	150	106	32.8	1.45	386
0	SD	0.43	0.18	1.0	7	3	2.8	0.25	163
mg/kg/dose	N	2	2	2	2	2	2	2	2
2	Mean	3.66	0.55	4.4	149	103	31.3	1.41	857
5	SD	0.62	0.04	0.2	4	0	0.4	0.02	824
mg/kg/dose	N	2	2	2	2	2	2	2	2
	%Diff	-12.66	0.92	-19.4	-1	-3	-4.6	-2.49	122
3	Mean	3.39	0.37	5.1	148	107	31.7	1.37	253
25	SD	1.69	0.00	0.6	1	1	3.3	0.15	7
mg/kg/dose	N	2	2	2	2	2	2	2	2
	%Diff	-19.12	-32.11	-5.6	-2	0	-3.4	-5.56	-34
4	Mean	3.80	0.68	4.4	147	105	28.6	1.51	320
100	SD	0.24	0.03	0.4	1	1	2.1	0.06	97
mg/kg/dose	N	2	2	2	2	2	2	2	2
	%Diff	-9.20	24.77	-18.5	-2	-1	-12.8	4.35	-17

Table 7 Summary of Chemistry-Recovery Phase (Cont'd)

Day: 210 Relative to Start Date

Sex: Male		ALT (U/L)	AST (U/L)	TP (g/L)	ALB (g/L)	TBIL (µmol/L)	ALP (U/L)	GGT (U/L)	sGLU (mmol/L)	UREA (mmol/L)	CRE (µmol/L)	Ca (mmol/L)	P (mmol/L)
1	Mean	45	51	70.7	42.7	3.84	788	87	3.53	6.90	55	2.49	2.15
0	SD	10	2	2.5	0.4	2.03	237	18	0.74	0.11	5	0.04	0.33
mg/kg/dose	N	2	2	2	2	2	2	2	2	2	2	2	2
2	Mean	52	72	69.7	42.8	6.24	909	65	3.65	6.79	59	2.52	2.30
5	SD	8	41	5.6	0.4	0.96	325	10	0.35	0.08	16	0.09	0.33
mg/kg/dose	N	2	2	2	2	2	2	2	2	2	2	2	2
	%Diff	14	43	-1.4	0.2	62.71	15	-25	3.55	-1.67	7	1.00	7.23
3	Mean	47	34	74.5	45.4	4.01	772	71	3.51	6.62	68	2.52	2.31
25	SD	17	7	5.3	4.9	0.13	7	17	1.17	0.44	11	0.05	0.01
mg/kg/dose	N	2	2	2	2	2	2	2	2	2	2	2	2
	%Diff	4	-33	5.4	6.2	4.56	-2	-18	-0.43	-4.06	25	1.00	7.69
4	Mean	56	50	76.6	46.0	6.55	911	79	3.27	7.75	56	2.46	2.14
100	SD	4	5	6.6	1.8	0.50	336	1	0.42	0.97	10	0.08	0.39
mg/kg/dose	N	2	2	2	2	2	2	2	2	2	2	2	2
	%Diff	23	-2	8.4	7.7	70.66	16	-9	-7.23	12.25	3	-1.20	-0.47

Table 7 Summary of Chemistry-Recovery Phase (Cont'd)

Day: 210 Relative to Start Date

Sex: Male		TCHO (mmol/L)	TG (mmol/L)	K (mmol/L)	Na (mmol/L)	Cl (mmol/L)	GLB (g/L)	A/G	CK (U/L)
1	Mean	2.89	0.25	4.5	149	107	28.0	1.53	1189
0	SD	0.26	0.07	0.1	3	1	2.1	0.10	469
mg/kg/dose	N	2	2	2	2	2	2	2	2
2	Mean	3.48	0.63	4.6	147	104	26.9	1.62	2535
5	SD	0.37	0.02	0.2	1	1	5.2	0.30	3260
mg/kg/dose	N	2	2	2	2	2	2	2	2
	%Diff	20.45	150.00	2.2	-1	-2	-3.9	5.96	113
3	Mean	3.67	0.48	5.2	147	105	29.1	1.56	627
25	SD	0.89	0.08	0.7	1	1	0.4	0.14	648
mg/kg/dose	N	2	2	2	2	2	2	2	2
	%Diff	27.21	90.00	16.9	-1	-2	4.1	1.70	-47
4	Mean	3.45	0.62	4.6	145	102	30.6	1.52	513
100	SD	0.49	0.18	0.3	1	1	4.8	0.18	180
mg/kg/dose	N	2	2	2	2	2	2	2	2
	%Diff	19.41	146.00	3.4	-3	-4	9.5	-0.92	-57

Table 8 Summary of Urinalysis

Table 8 Summary of Urinalysis

Key Page

Measurement Descriptions

Headings Used	Description
pH	Urine pH
SG	Urine Specific Gravity
Volume	Volume

Table 8 Summary of Urinalysis-Pretest and Dosing Phase

Day(s) Relative to Start Date

Sex: Male		pH	pH	pH	pH	pH	SG	SG	SG	SG	SG	Volume (mL) [a]	Volume (mL) [a]
		-11	53	87	123	179	-11	53	87	123	179	-11	53
1	Mean	6.2	7.2	7.1	6.8	6.9	1.013	1.014	1.015	1.012	1.015	19	17
0	SD	1.3	0.9	0.6	1.3	0.6	0.007	0.003	0.005	0.005	0.003	5	6
mg/kg/dose	N	6	6	6	6	6	6	6	6	6	6	6	6
2	Mean	6.5	7.6	7.3	6.8	7.1	1.018	1.021	1.019	1.015	1.016	14	17
5	SD	0.8	0.6	0.5	0.9	0.7	0.010	0.004	0.007	0.005	0.009	7	8
mg/kg/dose	N	6	6	6	6	6	6	6	6	6	6	6	6
	%Diff	5.4	5.8	3.5	0.0	2.4	0.510	0.674	0.427	0.280	0.016	-26	-3
3	Mean	5.8	7.0	6.6	7.2	7.0	1.019	1.015	1.016	1.013	1.012	16	19
25	SD	1.0	0.4	0.9	0.9	0.8	0.008	0.008	0.009	0.006	0.007	6	6
mg/kg/dose	N	6	6	6	6	6	6	6	6	6	6	6	6
	%Diff	-6.8	-2.3	-7.1	6.2	1.2	0.592	0.132	0.148	0.033	-0.312	-17	7
4	Mean	5.9	6.8	7.0	6.7	6.3	1.012	1.008	1.009	1.012	1.011	21	18
100	SD	0.9	0.4	0.9	0.7	0.6	0.009	0.006	0.007	0.005	0.007	10	11
mg/kg/dose	N	6	6	6	6	6	6	6	6	6	6	6	6
	%Diff	-4.1	-4.7	-1.2	-1.2	-8.4	-0.082	-0.559	-0.608	-0.033	-0.443	9	5

[a] - Anova & Dunnett

Table 8 Summary of Urinalysis-Pretest and Dosing Phase (Cont'd)

Day(s) Relative to Start Date

Sex: Male		Volume (mL) [a] 87	Volume (mL) [a] 123	Volume (mL) [a] 179
1	Mean	24	18	19
0	SD	9	3	8
mg/kg/dose	N	6	6	6
2	Mean	19	13	18
5	SD	3	6	7
mg/kg/dose	N	6	6	6
	%Diff	-19	-29	-5
3	Mean	20	13	16
25	SD	10	5	5
mg/kg/dose	N	6	6	6
	%Diff	-15	-29	-19
4	Mean	28	17	16
100	SD	12	7	7
mg/kg/dose	N	6	6	6
	%Diff	18	-6	-20

[a] - Anova & Dunnett

Table 8 Summary of Urinalysis-Pretest and Dosing Phase (Cont'd)

Day(s) Relative to Start Date

Sex: Female		pH	pH	pH	pH	pH	SG	SG	SG	SG	SG	Volume (mL) [a]	Volume (mL) [a1]
		-12	52	86	122	178	-12	52	86	122	178	-12	52
1	Mean	7.0	7.3	7.1	7.1	6.8	1.015	1.012	1.020	1.013	1.011	19	22
0	SD	0.7	0.6	1.0	0.7	1.0	0.008	0.004	0.006	0.006	0.005	12	5
mg/kg/dose	N	6	6	6	6	6	6	6	6	6	6	6	6
2	Mean	7.2	7.5	7.4	6.7	7.0	1.015	1.013	1.021	1.014	1.015	18	19
5	SD	0.7	0.6	0.7	0.4	0.8	0.007	0.008	0.006	0.006	0.004	6	11
mg/kg/dose	N	6	6	6	6	6	6	6	6	6	6	6	6
	%Diff	2.4	2.3	4.7	-5.9	3.7	0.033	0.082	0.131	0.033	0.379	-5	-15
3	Mean	6.9	7.1	6.8	6.9	6.9	1.018	1.014	1.016	1.013	1.015	24	18
25	SD	0.9	0.7	1.0	1.1	1.1	0.008	0.008	0.007	0.005	0.003	14	2
mg/kg/dose	N	6	6	6	6	6	6	6	6	6	6	6	6
	%Diff	-1.2	-3.4	-3.5	-2.4	2.5	0.263	0.132	-0.343	-0.066	0.379	25	-17
4	Mean	7.4	7.4	7.0	6.8	6.7	1.016	1.017	1.016	1.018	1.010	20	16
100	SD	0.4	0.6	1.1	0.9	0.9	0.010	0.008	0.008	0.010	0.007	5	6
mg/kg/dose	N	6	6	6	6	6	6	6	6	6	6	6	6
	%Diff	6.0	1.1	-1.2	-4.7	-1.2	0.115	0.428	-0.409	0.461	-0.082	5	-26

[a] - Anova & Dunnett
[a1] - Kruskal-Wallis & Dunnett on Ranks

Table 8 Summary of Urinalysis-Pretest and Dosing Phase (Cont'd)

Day(s) Relative to Start Date

Sex: Female		Volume (mL) [a] 86	Volume (mL) [a] 122	Volume (mL) [a1] 178
1	Mean	22	18	22
0	SD	2	5	11
mg/kg/dose	N	6	6	6
2	Mean	18	15	17
5	SD	4	6	5
mg/kg/dose	N	6	6	6
	%Diff	-16	-16	-23
3	Mean	20	16	20
25	SD	3	3	3
mg/kg/dose	N	6	6	6
	%Diff	-9	-13	-11
4	Mean	17	13	13
100	SD	6	8	5
mg/kg/dose	N	6	6	6
	%Diff	-19	-28	-42

[a] - Kruskal-Wallis & Dunnett on Ranks
[a1] - Anova & Dunnett

Table 8 Summary of Urinalysis-Recovery Phase

Day: 209 Relative to Start Date

Sex: Female		pH	SG	Volume (mL)
1	Mean	7.3	1.017	30
0	SD	0.4	0.013	11
mg/kg/dose	N	2	2	2
2	Mean	7.8	1.018	40
5	SD	0.4	0.004	1
mg/kg/dose	N	2	2	2
	%Diff	6.9	0.098	36
3	Mean	7.3	1.010	44
25	SD	0.4	0.014	12
mg/kg/dose	N	2	2	2
	%Diff	0.0	-0.639	47
4	Mean	7.0	1.012	55
100	SD	0.0	0.014	41
mg/kg/dose	N	2	2	2
	%Diff	-3.4	-0.443	86

Table 8 Summary of Urinalysis-Recovery Phase (Cont'd)

Day: 210 Relative to Start Date

Sex: Male		pH	SG	Volume (mL)
1	Mean	7.8	1.014	50
0	SD	0.4	0.005	23
mg/kg/dose	N	2	2	2
2	Mean	7.5	1.018	37
5	SD	1.4	0.008	5
mg/kg/dose	N	2	2	2
	%Diff	-3.2	0.444	-27
3	Mean	6.8	1.018	12
25	SD	1.8	0.004	11
mg/kg/dose	N	2	2	2
	%Diff	-12.9	0.444	-76
4	Mean	6.5	1.009	43
100	SD	0.7	0.011	41
mg/kg/dose	N	2	2	2
	%Diff	-16.1	-0.444	-14

Table 9 Summary of Immunoglobin and Complement

Table 9 Summary of Immunoglobin and Complement

Key Page

Measurement Descriptions

Headings Used	Description
C3c	Complement C3c
C4	Complement C4
IgG	Immunoglobulin G
IgA	Immunoglobulin A
IgM	Immunoglobulin M

Table 9 Summary of Immunoglobin and Complement-Pretest and Dosing Phase

Day(s) Relative to Start Date

Sex: Male		C3c (g/L) [a]	C3c (g/L) [a]	C3c (g/L) [a]	C3c (g/L) [a]	C4 (g/L) [a]	C4 (g/L) [a]	C4 (g/L) [a]	C4 (g/L) [a1]	IgG (g/L) [a]	IgG (g/L) [a]	IgG (g/L) [a]	IgG (g/L) [a]
		-6	1	88	178	-6	1	88	178	-6	1	88	178
1	Mean	1.32	1.21	1.17	1.06	0.18	0.18	0.18	0.18	9.69	9.28	8.31	9.57
0	SD	0.20	0.11	0.09	0.11	0.04	0.05	0.03	0.06	1.08	0.93	1.07	1.29
mg/kg/dose	N	6	6	6	6	6	6	6	6	6	6	6	6
2	Mean	1.23	1.17	1.16	0.97	0.17	0.16	0.20	0.16	8.90	8.07	8.23	9.14
5	SD	0.24	0.15	0.17	0.11	0.07	0.07	0.09	0.06	1.29	1.57	1.32	1.11
mg/kg/dose	N	6	6	6	6	6	6	6	6	6	6	6	6
	%Diff	-6.70	-3.57	-1.28	-8.68	-8.33	-9.43	10.38	-7.55	-8.07	-13.06	-0.98	-4.56
3	Mean	1.23	1.10	1.07	1.01	0.20	0.19	0.21	0.19	8.29	8.20	8.33	9.57
25	SD	0.16	0.09	0.10	0.11	0.08	0.07	0.10	0.09	1.35	1.20	0.88	0.91
mg/kg/dose	N	6	6	6	6	6	6	6	6	6	6	6	6
	%Diff	-7.08	-9.48	-8.53	-4.73	12.96	4.72	17.92	5.66	-14.40	-11.66	0.20	0.00
4	Mean	1.38	1.21	1.10	0.94	0.22	0.20	0.19	0.15	9.76	11.10	12.43***	13.13***
100	SD	0.26	0.15	0.08	0.08	0.06	0.06	0.06	0.04	1.50	1.64	1.53	1.50
mg/kg/dose	N	6	6	6	6	6	6	6	6	6	6	6	6
	%Diff	4.55	0.00	-6.40	-11.04	19.44	12.26	5.66	-13.21	0.77	19.61	49.46	37.19

[a] - Anova & Dunnett: *** = $p \leq 0.001$
[a1] - Kruskal-Wallis & Dunnett on Ranks

Table 9 Summary of Immunoglobin and Complement-Pretest and Dosing Phase (Cont'd)

Day(s) Relative to Start Date

Sex: Male		IgA (g/L) [a] -6	IgA (g/L) [a] 1	IgA (g/L) [a] 88	IgA (g/L) [a] 178	IgM (g/L) [a] -6	IgM (g/L) [a] 1	IgM (g/L) [a] 88	IgM (g/L) [a] 178
1	Mean	2.11	1.94	1.72	1.72	1.00	0.86	0.72	0.71
0	SD	0.79	0.77	0.58	0.57	0.41	0.32	0.31	0.34
mg/kg/dose	N	6	6	6	6	6	6	6	6
2	Mean	1.45	1.30	1.43	1.52	0.94	0.82	0.69	0.66
5	SD	0.60	0.54	0.57	0.58	0.49	0.37	0.33	0.31
mg/kg/dose	N	6	6	6	6	6	6	6	6
	%Diff	-31.39	-32.73	-17.04	-11.53	-5.19	-4.45	-3.93	-6.60
3	Mean	1.66	1.49	1.46	1.57	1.07	1.00	0.94	0.83
25	SD	0.52	0.52	0.49	0.31	0.62	0.61	0.61	0.50
mg/kg/dose	N	6	6	6	6	6	6	6	6
	%Diff	-21.69	-23.17	-15.49	-8.82	7.20	15.47	29.56	16.98
4	Mean	1.97	1.77	1.75	1.60	0.92	0.83	0.77	0.70
100	SD	0.63	0.56	0.55	0.45	0.14	0.20	0.26	0.20
mg/kg/dose	N	6	6	6	6	6	6	6	6
	%Diff	-6.86	-8.79	1.65	-6.88	-8.04	-3.48	7.16	-1.65

[a] - Anova & Dunnett

Table 9 Summary of Immunoglobin and Complement-Pretest and Dosing Phase (Cont'd)

Day(s) Relative to Start Date

Sex: Female		C3c (g/L) [a]	C3c (g/L) [a]	C3c (g/L) [a1]	C3c (g/L) [a]	C4 (g/L) [a]	C4 (g/L) [a]	C4 (g/L) [a]	C4 (g/L) [a]	IgG (g/L) [a]	IgG (g/L) [a]	IgG (g/L) [a]	IgG (g/L) [a]
		-7	1	87	177	-7	1	87	177	-7	1	87	177
1	Mean	1.24	1.21	1.11	0.98	0.20	0.20	0.19	0.15	10.66	9.72	9.62	11.54
0	SD	0.15	0.18	0.17	0.20	0.04	0.07	0.04	0.03	2.67	3.55	2.68	3.68
mg/kg/dose	N	6	6	6	6	6	6	6	6	6	6	6	6
2	Mean	1.30	1.20	1.06	0.96	0.20	0.20	0.19	0.17	12.47	12.45	11.19	13.44
5	SD	0.20	0.11	0.06	0.13	0.05	0.05	0.03	0.07	1.41	2.14	1.29	1.73
mg/kg/dose	N	6	6	6	6	6	6	6	6	6	6	6	6
	%Diff	4.70	-0.69	-4.07	-2.04	-0.84	-3.31	-1.75	12.22	16.91	27.99	16.25	16.45
3	Mean	1.18	1.06	1.01	0.95	0.20	0.19	0.20	0.19	10.63	10.39	9.97	12.36
25	SD	0.21	0.14	0.05	0.14	0.05	0.08	0.05	0.04	1.63	1.64	1.45	1.27
mg/kg/dose	N	6	6	6	6	6	6	6	6	6	6	6	6
	%Diff	-5.11	-12.28	-8.45	-2.90	0.84	-4.96	5.26	27.78	-0.31	6.89	3.64	7.11
4	Mean	1.20	1.08	0.99	0.89	0.24	0.22	0.19	0.17	8.82	10.18	10.59	12.88
100	SD	0.09	0.09	0.06	0.09	0.06	0.06	0.03	0.05	1.80	1.52	1.24	2.07
mg/kg/dose	N	6	6	6	6	6	6	6	6	6	6	6	6
	%Diff	-3.63	-10.90	-10.86	-8.86	20.17	9.92	-0.88	13.33	-17.24	4.66	10.08	11.61

[a] - Anova & Dunnett
[a1] - Kruskal-Wallis & Dunnett on Ranks

Table 9 Summary of Immunoglobin and Complement-Pretest and Dosing Phase (Cont'd)

Day(s) Relative to Start Date

Sex: Female		IgA (g/L) [a] -7	IgA (g/L) [a] 1	IgA (g/L) [a] 87	IgA (g/L) [a] 177	IgM (g/L) [a] -7	IgM (g/L) [a] 1	IgM (g/L) [a] 87	IgM (g/L) [a] 177
1	Mean	1.41	1.30	1.49	1.48	1.27	1.15	1.01	1.06
0	SD	0.62	0.55	0.57	0.53	0.54	0.47	0.42	0.40
mg/kg/dose	N	6	6	6	6	6	6	6	6
2	Mean	1.51	1.50	1.60	1.53	1.19	1.08	0.92	0.98
5	SD	0.62	0.57	0.63	0.72	0.58	0.58	0.41	0.35
mg/kg/dose	N	6	6	6	6	6	6	6	6
	%Diff	6.73	15.55	7.25	3.73	-6.41	-5.68	-8.62	-7.40
3	Mean	1.78	1.62	1.88	1.83	1.30	1.09	1.08	1.14
25	SD	0.62	0.53	1.04	0.92	0.39	0.33	0.35	0.34
mg/kg/dose	N	6	6	6	6	6	6	6	6
	%Diff	26.21	24.55	26.00	24.07	2.09	-5.24	6.97	7.40
4	Mean	1.46	1.40	1.47	1.36	1.02	0.88	0.91	0.86
100	SD	0.65	0.66	0.52	0.40	0.39	0.31	0.44	0.46
mg/kg/dose	N	6	6	6	6	6	6	6	6
	%Diff	3.54	7.97	-1.56	-8.14	-19.76	-23.14	-9.12	-19.21

[a] - Anova & Dunnett

Table 9 Summary of Immunoglobin and Complement-Recovery Phase

Day: 210 Relative to Start Date

Sex: Female		C3c (g/L)	C4 (g/L)	IgG (g/L)	IgA (g/L)	IgM (g/L)
1	Mean	0.96	0.12	12.60	1.34	0.92
0	SD	0.12	0.01	3.85	0.54	0.43
mg/kg/dose	N	2	2	2	2	2
2	Mean	0.88	0.17	14.13	1.14	0.83
5	SD	0.04	0.04	0.58	0.84	0.06
mg/kg/dose	N	2	2	2	2	2
	%Diff	-7.85	41.67	12.14	-14.98	-9.84
3	Mean	0.86	0.13	11.44	2.29	1.38
25	SD	0.13	0.00	1.97	1.20	0.14
mg/kg/dose	N	2	2	2	2	2
	%Diff	-9.95	8.33	-9.21	71.16	50.82
4	Mean	0.83	0.17	9.03	1.50	1.09
100	SD	0.01	0.02	0.06	0.08	0.59
mg/kg/dose	N	2	2	2	2	2
	%Diff	-13.61	37.50	-28.37	11.99	19.13

Table 9 Summary of Immunoglobin and Complement-Recovery Phase (Cont'd)

Day: 211 Relative to Start Date

Sex: Male		C3c (g/L)	C4 (g/L)	IgG (g/L)	IgA (g/L)	IgM (g/L)
1	Mean	0.99	0.16	9.06	1.69	0.85
0	SD	0.06	0.08	1.95	0.93	0.40
mg/kg/dose	N	2	2	2	2	2
2	Mean	0.85	0.13	9.65	1.22	0.47
5	SD	0.05	0.05	2.29	0.69	0.35
mg/kg/dose	N	2	2	2	2	2
	%Diff	-14.21	-21.88	6.51	-28.11	-45.29
3	Mean	0.95	0.25	8.88	1.38	0.64
25	SD	0.11	0.03	0.56	0.10	0.32
mg/kg/dose	N	2	2	2	2	2
	%Diff	-4.06	56.25	-2.04	-18.34	-25.29
4	Mean	0.90	0.14	10.42	1.77	0.53
100	SD	0.01	0.04	1.80	0.39	0.16
mg/kg/dose	N	2	2	2	2	2
	%Diff	-8.63	-12.50	14.96	4.44	-38.24

Table 1 Summary of Immunophenotyping Results-Monkey Blood (CD45+CD3+, Total T Cells)

Animal No.	Subpopulation percent (%) for T/B Panel (Male)				
	Pretest	Day 1 2~4h	Day 88	Day 178	Day 211
1001	65.86	54.33	63.33	68.76	NA
1002	62.88	56.50	64.79	66.92	NA
1003	63.69	62.57	55.87	63.28	NA
1004	63.57	59.57	66.70	74.52	NA
1005	63.37	59.09	61.06	67.57	66.49
1006	34.54	29.52	45.37	54.71	57.69
**Mean**	**58.99**	**53.60**	**59.52**	**65.96**	**62.09**
**SD**	**12.02**	**12.12**	**7.87**	**6.61**	**6.22**
2001	53.42	52.01	65.20	73.68	NA
2002	68.86	54.31	57.89	69.52	NA
2003	56.25	55.54	61.83	71.19	NA
2004	57.28	42.99	60.66	74.13	NA
2005	59.04	53.47	60.48	71.69	70.00
2006	54.76	51.27	68.28	76.05	79.22
**Mean**	**58.27**	**51.60**	**62.39**	**72.71**	**74.61**
**SD**	**5.54**	**4.49**	**3.74**	**2.35**	**6.52**
3001	60.71	55.96	56.68	66.83	NA
3002	56.79	55.23	58.29	66.37	NA
3003	71.53	65.61	72.66	74.19	NA
3004	74.36	71.01	74.71	82.21	NA
3005	55.79	48.15	52.26	55.75	58.11
3006	68.69	64.83	72.75	78.14	77.54
**Mean**	**64.65**	**60.13**	**64.56**	**70.58**	**67.83**
**SD**	**7.92**	**8.43**	**9.88**	**9.56**	**13.74**
4001	59.71	55.17	69.21	71.41	NA
4002	70.61	64.81	64.92	68.34	NA
4003	48.66	43.16	49.26	55.35	NA
4004	61.84	50.29	67.47	75.62	NA
4005	57.10	50.70	68.00	80.73	79.90
4006	60.83	57.38	72.62	79.00	72.86
**Mean**	**59.79**	**53.59**	**65.25**	**71.74**	**76.38**
**SD**	**7.12**	**7.36**	**8.23**	**9.26**	**4.98**

Table 2 Summary of Immunophenotyping Results-Monkey Blood (CD45+CD3+CD4+CD8-, Helper T Cells)

	Subpopulation percent (%) for T/B Panel (Male)				
Animal No.	Pretest	Day 1 2~4h	Day 88	Day 178	Day 211
1001	22.23	26.66	25.10	30.80	NA
1002	42.81	37.48	43.48	43.90	NA
1003	43.52	42.20	35.53	38.70	NA
1004	34.51	33.25	36.85	41.26	NA
1005	32.40	30.28	30.31	35.03	31.02
1006	20.80	18.35	28.84	35.53	37.71
**Mean**	**32.71**	**31.37**	**33.35**	**37.54**	**34.37**
**SD**	**9.74**	**8.38**	**6.59**	**4.72**	**4.73**
2001	25.68	27.17	38.40	43.80	NA
2002	43.02	32.03	31.96	41.47	NA
2003	26.97	25.48	26.10	33.24	NA
2004	32.55	24.46	33.87	44.37	NA
2005	26.96	26.05	29.67	35.08	34.06
2006	34.64	33.73	37.76	39.75	43.32
**Mean**	**31.64**	**28.15**	**32.96**	**39.62**	**38.69**
**SD**	**6.61**	**3.80**	**4.74**	**4.58**	**6.55**
3001	37.67	31.82	32.12	38.96	NA
3002	34.60	32.36	33.12	39.73	NA
3003	38.50	38.30	39.50	39.90	NA
3004	41.68	41.25	40.68	47.08	NA
3005	29.99	26.32	29.65	28.77	32.38
3006	42.52	39.27	43.82	47.90	46.90
**Mean**	**37.49**	**34.89**	**36.48**	**40.39**	**39.64**
**SD**	**4.66**	**5.67**	**5.61**	**6.91**	**10.27**
4001	29.70	30.26	36.32	33.20	NA
4002	36.43	33.76	31.05	32.49	NA
4003	19.57	18.44	21.68	21.91	NA
4004	31.09	26.19	30.12	31.96	NA
4005	32.84	28.17	42.42	55.07	54.86
4006	35.52	33.73	40.93	42.77	40.68
**Mean**	**30.86**	**28.43**	**33.75**	**36.23**	**47.77**
**SD**	**6.09**	**5.74**	**7.74**	**11.35**	**10.03**

Table 3 Summary of Immunophenotyping Results-Monkey Blood (CD45+CD3+CD4-CD8+, Cytotoxic T Cells)

	Subpopulation percent (%) for T/B Panel (Male)				
Animal No.	Pretest	Day 1 2~4h	Day 88	Day 178	Day 211
1001	39.95	23.50	33.66	33.56	NA
1002	15.87	14.75	16.50	17.67	NA
1003	16.16	16.93	15.79	20.52	NA
1004	24.84	22.38	24.26	28.18	NA
1005	26.82	25.18	26.35	27.75	30.94
1006	11.12	8.28	13.18	15.95	16.94
**Mean**	**22.46**	**18.50**	**21.62**	**23.94**	**23.94**
**SD**	**10.41**	**6.41**	**7.82**	**6.93**	**9.90**
2001	23.28	20.52	22.71	26.56	NA
2002	22.56	18.67	22.23	24.44	NA
2003	25.58	26.55	29.39	31.51	NA
2004	18.83	13.79	20.77	23.55	NA
2005	27.51	23.69	26.32	32.90	32.52
2006	15.87	13.55	24.91	30.00	29.12
**Mean**	**22.27**	**19.46**	**24.39**	**28.16**	**30.82**
**SD**	**4.30**	**5.23**	**3.15**	**3.87**	**2.40**
3001	18.56	19.21	19.38	22.55	NA
3002	18.85	19.87	21.24	22.63	NA
3003	29.23	23.72	29.40	30.75	NA
3004	28.41	24.63	30.02	30.46	NA
3005	20.91	17.36	18.67	22.32	22.03
3006	21.13	20.25	24.58	26.23	26.80
**Mean**	**22.85**	**20.84**	**23.88**	**25.82**	**24.42**
**SD**	**4.75**	**2.78**	**4.96**	**3.98**	**3.37**
4001	25.34	20.43	29.07	33.98	NA
4002	29.61	26.67	29.82	31.55	NA
4003	25.45	20.39	22.77	27.86	NA
4004	22.25	17.01	28.81	35.88	NA
4005	19.81	16.88	20.68	20.98	21.02
4006	22.13	20.10	28.84	33.55	29.44
**Mean**	**24.10**	**20.25**	**26.67**	**30.63**	**25.23**
**SD**	**3.45**	**3.55**	**3.90**	**5.46**	**5.95**

Table 4 Summary of Immunophenotyping Results-Monkey Blood (CD45+CD3-CD20+，B Cells)

	Subpopulation percent (%) for T/B Panel (Male)				
Animal No.	Pretest	Day 1 2~4h	Day 88	Day 178	Day 211
1001	20.76	34.61	21.87	16.39	NA
1002	22.23	29.73	24.97	21.66	NA
1003	25.90	31.49	34.03	28.21	NA
1004	31.24	33.25	23.10	18.92	NA
1005	24.31	25.98	27.79	23.54	24.27
1006	38.72	55.28	26.57	21.43	26.85
**Mean**	**27.19**	**35.06**	**26.39**	**21.69**	**25.56**
**SD**	**6.71**	**10.35**	**4.33**	**4.04**	**1.82**
2001	30.14	37.06	27.19	17.61	NA
2002	26.37	37.89	35.17	23.62	NA
2003	32.27	32.92	17.44	15.69	NA
2004	28.93	46.89	26.67	17.68	NA
2005	35.15	41.54	33.96	24.19	25.37
2006	37.80	46.04	26.39	16.77	14.18
**Mean**	**31.78**	**40.39**	**27.80**	**19.26**	**19.78**
**SD**	**4.19**	**5.45**	**6.38**	**3.67**	**7.91**
3001	26.64	30.38	31.92	21.30	NA
3002	20.81	27.90	17.81	18.43	NA
3003	19.41	30.20	19.34	19.09	NA
3004	15.74	20.65	15.59	10.86	NA
3005	22.38	33.28	32.31	21.69	25.74
3006	23.32	27.72	17.96	16.90	15.90
**Mean**	**21.38**	**28.36**	**22.49**	**18.05**	**20.82**
**SD**	**3.70**	**4.28**	**7.55**	**3.95**	**6.96**
4001	31.17	39.83	21.15	16.25	NA
4002	20.94	26.52	20.55	15.40	NA
4003	45.76	50.18	47.34	41.93	NA
4004	32.40	44.86	24.95	19.03	NA
4005	34.28	43.78	22.96	13.80	13.91
4006	33.94	38.14	23.09	15.73	23.30
**Mean**	**33.08**	**40.55**	**26.67**	**20.36**	**18.61**
**SD**	**7.93**	**8.06**	**10.24**	**10.71**	**6.64**

Table 5 Summary of Immunophenotyping Results-Monkey Blood (CD45+CD3+, Total T Cells)

	Subpopulation percent (%) for T/B Panel (Female)				
Animal No.	Pretest	Day 1 2~4h	Day 88	Day 178	Day 211
1501	58.85	46.12	67.53	63.79	NA
1502	70.24	61.75	71.01	74.49	NA
1503	71.36	69.94	72.24	77.39	NA
1504	64.03	53.01	61.77	63.32	NA
1505	55.22	50.61	55.89	62.53	58.22
1506	74.81	69.00	67.78	63.32	67.47
**Mean**	**65.75**	**58.41**	**66.04**	**67.47**	**62.85**
**SD**	**7.68**	**9.97**	**6.16**	**6.63**	**6.54**
2501	66.67	62.84	67.49	69.78	NA
2502	65.55	53.64	73.41	73.36	NA
2503	60.44	49.13	55.40	62.36	NA
2504	63.69	58.10	67.91	63.48	NA
2505	60.55	46.25	62.99	65.41	65.44
2506	56.24	43.88	52.99	50.78	59.40
**Mean**	**62.19**	**52.31**	**63.37**	**64.20**	**62.42**
**SD**	**3.87**	**7.27**	**7.87**	**7.75**	**4.27**
3501	59.94	54.33	51.08	56.24	NA
3502	71.57	67.43	68.57	64.95	NA
3503	68.78	65.62	68.47	72.46	NA
3504	54.63	53.36	61.10	66.15	NA
3505	57.65	54.83	67.89	73.86	58.06
3506	60.38	54.05	59.48	62.19	68.63
**Mean**	**62.16**	**58.27**	**62.77**	**65.98**	**63.35**
**SD**	**6.60**	**6.44**	**6.97**	**6.55**	**7.47**
4501	51.89	45.08	61.25	60.23	NA
4502	58.94	55.33	60.85	64.94	NA
4503	73.11	75.20	69.81	74.83	NA
4504	65.85	65.47	68.85	73.59	NA
4505	62.28	61.70	53.97	71.98	71.88
4506	73.01	67.96	77.52	82.82	84.25
**Mean**	**64.18**	**61.79**	**65.38**	**71.40**	**78.07**
**SD**	**8.28**	**10.51**	**8.33**	**7.92**	**8.75**

Table 6 Summary of Immunophenotyping Results-Monkey Blood (CD45+CD3+CD4+CD8-, Helper T Cells)

	Subpopulation percent (%) for T/B Panel (Female)				
Animal No.	Pretest	Day 1 2~4h	Day 88	Day 178	Day 211
1501	32.32	25.33	37.50	35.04	NA
1502	46.83	40.88	44.70	46.76	NA
1503	45.20	47.48	44.09	49.57	NA
1504	30.02	24.13	34.70	36.05	NA
1505	36.52	34.59	35.77	40.41	37.76
1506	39.88	42.25	26.86	28.15	31.40
**Mean**	**38.46**	**35.78**	**37.27**	**39.33**	**34.58**
**SD**	**6.79**	**9.50**	**6.62**	**7.94**	**4.50**
2501	31.42	31.24	31.61	32.94	NA
2502	36.64	33.27	33.04	31.33	NA
2503	36.92	29.90	31.64	37.75	NA
2504	38.63	35.88	41.09	39.00	NA
2505	39.25	30.30	39.10	39.01	39.54
2506	32.59	26.08	29.10	21.27	29.15
**Mean**	**35.91**	**31.11**	**34.26**	**33.55**	**34.35**
**SD**	**3.20**	**3.31**	**4.73**	**6.83**	**7.35**
3501	28.77	28.39	26.41	30.06	NA
3502	45.59	43.37	42.42	42.22	NA
3503	34.96	36.43	37.33	37.33	NA
3504	27.29	28.07	28.93	30.81	NA
3505	39.90	38.54	34.47	48.82	34.27
3506	38.77	35.39	37.98	36.23	40.31
**Mean**	**35.88**	**35.03**	**34.59**	**37.58**	**37.29**
**SD**	**6.99**	**5.94**	**5.99**	**7.10**	**4.27**
4501	30.76	27.58	38.54	35.99	NA
4502	26.28	26.97	24.03	24.47	NA
4503	45.32	49.01	42.13	44.51	NA
4504	37.46	38.58	40.41	41.03	NA
4505	34.16	33.08	33.50	38.17	36.80
4506	46.18	42.30	49.17	49.14	46.54
**Mean**	**36.69**	**36.25**	**37.96**	**38.89**	**41.67**
**SD**	**7.94**	**8.67**	**8.52**	**8.46**	**6.89**

Table 7 Summary of Immunophenotyping Results-Monkey Blood (CD45+CD3+CD4-CD8+, Cytotoxic T Cells)

	Subpopulation percent (%) for T/B Panel (Female)				
Animal No.	Pretest	Day 1 2~4h	Day 88	Day 178	Day 211
1501	22.57	16.51	25.89	24.33	NA
1502	18.84	15.70	20.60	21.17	NA
1503	23.59	19.63	25.52	24.79	NA
1504	30.27	24.86	23.37	23.84	NA
1505	15.38	12.53	16.39	18.52	16.75
1506	29.35	20.71	35.36	29.92	30.23
**Mean**	**23.33**	**18.32**	**24.52**	**23.76**	**23.49**
**SD**	**5.80**	**4.33**	**6.37**	**3.83**	**9.53**
2501	31.74	27.87	31.71	30.35	NA
2502	26.44	17.73	37.81	38.25	NA
2503	19.85	14.92	19.96	20.00	NA
2504	22.39	19.36	23.89	21.82	NA
2505	17.15	12.05	19.41	21.27	21.40
2506	20.93	15.04	19.17	26.11	26.44
**Mean**	**23.08**	**17.83**	**25.33**	**26.30**	**23.92**
**SD**	**5.23**	**5.53**	**7.75**	**6.99**	**3.56**
3501	26.35	21.11	20.15	21.24	NA
3502	22.02	19.86	22.04	18.75	NA
3503	30.52	25.78	27.47	31.25	NA
3504	24.38	22.28	29.33	32.76	NA
3505	14.77	12.76	30.37	22.47	21.11
3506	18.54	15.03	18.99	23.06	25.46
**Mean**	**22.76**	**19.47**	**24.73**	**24.92**	**23.29**
**SD**	**5.62**	**4.80**	**4.93**	**5.70**	**3.08**
4501	17.32	14.48	19.97	21.46	NA
4502	25.16	21.73	26.94	29.79	NA
4503	22.84	21.19	23.70	25.25	NA
4504	24.18	22.85	24.63	27.62	NA
4505	24.88	25.17	17.07	29.92	31.64
4506	19.59	18.31	22.23	25.08	28.67
**Mean**	**22.33**	**20.62**	**22.42**	**26.52**	**30.16**
**SD**	**3.19**	**3.75**	**3.51**	**3.25**	**2.10**

Table 8 Summary of Immunophenotyping Results-Monkey Blood (CD45+CD3-CD20+，B Cells)

	Subpopulation percent (%) for T/B Panel (Female)				
Animal No.	Pretest	Day 1 2~4h	Day 88	Day 178	Day 211
1501	28.96	39.90	18.35	16.22	NA
1502	18.34	24.24	15.33	15.66	NA
1503	20.73	19.61	19.41	16.45	NA
1504	22.97	29.85	18.54	14.17	NA
1505	26.42	32.66	29.61	17.99	19.77
1506	18.01	21.94	19.24	32.26	28.94
**Mean**	**22.57**	**28.03**	**20.08**	**18.79**	**24.36**
**SD**	**4.42**	**7.59**	**4.90**	**6.71**	**6.48**
2501	21.59	25.53	18.52	13.72	NA
2502	28.01	34.34	17.78	14.08	NA
2503	29.92	44.34	26.14	22.93	NA
2504	22.80	27.67	15.27	8.99	NA
2505	32.90	43.04	24.11	18.50	20.37
2506	26.88	35.81	28.57	32.76	26.70
**Mean**	**27.02**	**35.12**	**21.73**	**18.50**	**23.54**
**SD**	**4.27**	**7.70**	**5.28**	**8.44**	**4.48**
3501	28.12	37.16	45.26	37.45	NA
3502	18.93	27.91	22.71	32.23	NA
3503	23.20	28.48	21.80	17.35	NA
3504	33.48	37.07	26.55	24.17	NA
3505	32.78	33.26	27.16	11.89	12.77
3506	28.07	34.47	18.53	27.43	22.50
**Mean**	**27.43**	**33.06**	**27.00**	**25.09**	**17.64**
**SD**	**5.59**	**4.06**	**9.50**	**9.43**	**6.88**
4501	41.34	51.03	29.84	31.81	NA
4502	31.28	36.76	24.19	18.84	NA
4503	11.02	14.83	10.02	9.42	NA
4504	22.27	22.83	22.71	17.60	NA
4505	34.63	35.18	34.83	24.13	22.68
4506	24.34	29.97	20.11	13.31	13.33
**Mean**	**27.48**	**31.77**	**23.62**	**19.19**	**18.01**
**SD**	**10.64**	**12.47**	**8.52**	**7.95**	**6.61**

Table 25 Summary of cytokine results-monkey serum

	Cytokine Conc.(pg/mL), Pretest Male							
**Animal No.**	**IL-2**	**IL-5**	**IL-6**	**IL-10**	**IL-13**	**TNF-a**	**IFN-r**	**IL-4**
**1001**	<2.90	<2.70	8.80	<8.36	<2.63	<2.31	<2.67	<1.98
**1002**	49.99	<2.70	10.52	<8.36	<2.63	<2.31	<2.67	<1.98
**1003**	<2.90	<2.70	2.83	<8.36	<2.63	<2.31	<2.67	<1.98
**1004**	<2.90	<2.70	<2.61	<8.36	<2.63	<2.31	<2.67	<1.98
**1005**	<2.90	<2.70	<2.61	<8.36	<2.63	<2.31	<2.67	<1.98
**1006**	<2.90	<2.70	<2.61	<8.36	<2.63	<2.31	<2.67	<1.98
**Mean**	**Ncal**	**Ncal**	**Ncal**	**Ncal**	**Ncal**	**Ncal**	**Ncal**	**Ncal**
**SD**	**Ncal**	**Ncal**	**Ncal**	**Ncal**	**Ncal**	**Ncal**	**Ncal**	**Ncal**
**2001**	<2.90	<2.70	<2.61	<8.36	<2.63	<2.31	<2.67	<1.98
**2002**	14.20	<2.70	<2.61	48.43	3.07	3.26	50.09	12.47
**2003**	<2.90	<2.70	<2.61	79.31	<2.63	6.86	36.05	10.48
**2004**	<2.90	<2.70	23.86	13.12	<2.63	<2.31	<2.67	<1.98
**2005**	<2.90	<2.70	<2.61	<8.36	<2.63	<2.31	<2.67	<1.98
**2006**	<2.90	<2.70	<2.61	<8.36	<2.63	<2.31	<2.67	<1.98
**Mean**	**Ncal**	**Ncal**	**Ncal**	**Ncal**	**Ncal**	**Ncal**	**Ncal**	**Ncal**
**SD**	**Ncal**	**Ncal**	**Ncal**	**Ncal**	**Ncal**	**Ncal**	**Ncal**	**Ncal**
**3001**	<2.90	<2.70	10.66	<8.36	<2.63	<2.31	<2.67	<1.98
**3002**	<2.90	<2.70	<2.61	<8.36	<2.63	<2.31	<2.67	<1.98
**3003**	<2.90	<2.70	<2.61	<8.36	<2.63	<2.31	<2.67	<1.98
**3004**	<2.90	<2.70	<2.61	<8.36	<2.63	<2.31	<2.67	<1.98
**3005**	<2.90	<2.70	<2.61	<8.36	<2.63	<2.31	<2.67	<1.98
**3006**	<2.90	3.49	9.32	<8.36	<2.63	<2.31	<2.67	<1.98
**Mean**	**Ncal**	**Ncal**	**Ncal**	**Ncal**	**Ncal**	**Ncal**	**Ncal**	**Ncal**
**SD**	**Ncal**	**Ncal**	**Ncal**	**Ncal**	**Ncal**	**Ncal**	**Ncal**	**Ncal**
**4001**	<2.90	<2.70	<2.61	<8.36	<2.63	<2.31	<2.67	<1.98
**4002**	<2.90	<2.70	<2.61	<8.36	<2.63	<2.31	<2.67	<1.98
**4003**	<2.90	<2.70	<2.61	<8.36	<2.63	<2.31	<2.67	<1.98
**4004**	<2.90	<2.70	<2.61	<8.36	4.86	<2.31	<2.67	<1.98
**4005**	<2.90	<2.70	<2.61	<8.36	<2.63	<2.31	<2.67	<1.98
**4006**	<2.90	<2.70	<2.61	<8.36	<2.63	<2.31	<2.67	<1.98
**Mean**	**Ncal**	**Ncal**	**Ncal**	**Ncal**	**Ncal**	**Ncal**	**Ncal**	**Ncal**
**SD**	**Ncal**	**Ncal**	**Ncal**	**Ncal**	**Ncal**	**Ncal**	**Ncal**	**Ncal**

NcalN: Curve fitting failed; N/A: Data marked out; ND: Missing original data

Table 26 Summary of cytokine results-monkey serum

	Cytokine Conc.(pg/mL), Pretest Female							
**Animal No.**	**IL-2**	**IL-5**	**IL-6**	**IL-10**	**IL-13**	**TNF-a**	**IFN-r**	**IL-4**
**1501**	<2.90	<2.70	8.80	<8.36	<2.63	<2.31	<2.67	<1.98
**1502**	49.99	<2.70	10.52	<8.36	<2.63	<2.31	<2.67	<1.98
**1503**	<2.90	<2.70	2.83	<8.36	<2.63	<2.31	<2.67	<1.98
**1504**	<2.90	<2.70	<2.61	<8.36	<2.63	<2.31	<2.67	<1.98
**1505**	<2.90	<2.70	<2.61	<8.36	<2.63	<2.31	<2.67	<1.98
**1506**	<2.90	<2.70	<2.61	<8.36	<2.63	<2.31	<2.67	<1.98
**Mean**	**Ncal**	**Ncal**	**Ncal**	**Ncal**	**Ncal**	**Ncal**	**Ncal**	**Ncal**
**SD**	**Ncal**	**Ncal**	**Ncal**	**Ncal**	**Ncal**	**Ncal**	**Ncal**	**Ncal**
**2501**	<2.90	<2.70	<2.61	<8.36	<2.63	<2.31	<2.67	<1.98
**2502**	14.20	<2.70	<2.61	48.43	3.07	3.26	50.09	12.47
**2503**	<2.90	<2.70	<2.61	79.31	<2.63	6.86	36.05	10.48
**2504**	<2.90	<2.70	23.86	13.12	<2.63	<2.31	<2.67	<1.98
**2505**	<2.90	<2.70	<2.61	<8.36	<2.63	<2.31	<2.67	<1.98
**2506**	<2.90	<2.70	<2.61	<8.36	<2.63	<2.31	<2.67	<1.98
**Mean**	**Ncal**	**Ncal**	**Ncal**	**Ncal**	**Ncal**	**Ncal**	**Ncal**	**Ncal**
**SD**	**Ncal**	**Ncal**	**Ncal**	**Ncal**	**Ncal**	**Ncal**	**Ncal**	**Ncal**
**3501**	<2.90	<2.70	10.66	<8.36	<2.63	<2.31	<2.67	<1.98
**3502**	<2.90	<2.70	<2.61	<8.36	<2.63	<2.31	<2.67	<1.98
**3503**	<2.90	<2.70	<2.61	<8.36	<2.63	<2.31	<2.67	<1.98
**3504**	<2.90	<2.70	<2.61	<8.36	<2.63	<2.31	<2.67	<1.98
**3505**	<2.90	<2.70	<2.61	<8.36	<2.63	<2.31	<2.67	<1.98
**3506**	<2.90	3.49	9.32	<8.36	<2.63	<2.31	<2.67	<1.98
**Mean**	**Ncal**	**Ncal**	**Ncal**	**Ncal**	**Ncal**	**Ncal**	**Ncal**	**Ncal**
**SD**	**Ncal**	**Ncal**	**Ncal**	**Ncal**	**Ncal**	**Ncal**	**Ncal**	**Ncal**
**4501**	<2.90	<2.70	<2.61	<8.36	<2.63	<2.31	<2.67	<1.98
**4502**	<2.90	<2.70	<2.61	<8.36	<2.63	<2.31	<2.67	<1.98
**4503**	<2.90	<2.70	<2.61	<8.36	<2.63	<2.31	<2.67	<1.98
**4504**	<2.90	<2.70	<2.61	<8.36	4.86	<2.31	<2.67	<1.98
**4505**	<2.90	<2.70	<2.61	<8.36	<2.63	<2.31	<2.67	<1.98
**4506**	<2.90	<2.70	<2.61	<8.36	<2.63	<2.31	<2.67	<1.98
**Mean**	**Ncal**	**Ncal**	**Ncal**	**Ncal**	**Ncal**	**Ncal**	**Ncal**	**Ncal**
**SD**	**Ncal**	**Ncal**	**Ncal**	**Ncal**	**Ncal**	**Ncal**	**Ncal**	**Ncal**

NcalN: Curve fitting failed; N/A: Data marked out; ND: Missing original data
